# Silica Nanoparticles with Virus-Mimetic Spikes Enable Efficient siRNA Delivery In Vitro and In Vivo

**DOI:** 10.34133/research.0014

**Published:** 2022-12-21

**Authors:** Jianye Fu, Wenwei Han, Xue Zhang, Yutong Sun, Rajendra Bhadane, Bo Wei, Li Li, Liangmin Yu, Jinbo Yang, Jessica M. Rosenholm, Outi M. H. Salo-Ahen, Taojian Fan, Bin Zhang, Wageh Swelm, Ahmed A. Al-Ghamdi, Lin Xia, Han Zhang, Meng Qiu, Hongbo Zhang, Xin Wang

**Affiliations:** ^1^ Key Laboratory of Marine Drugs, Chinese Ministry of Education, School of Medicine and Pharmacy, Ocean University of China, Qingdao 266003, China.; ^2^ Collaborative Innovation Center for Optoelectronic Science & Technology, International Collaborative Laboratory of 2D Materials for Optoelectronics Science and Technology of Ministry of Education, Institute of Microscale Optoelectronics, College of Physics and Optoelectronic Engineering, Shenzhen University, Shenzhen 518060, China.; ^3^ Key Laboratory of Marine Chemistry Theory and Technology (Ocean University of China), Ministry of Education, Qingdao 266100, China.; ^4^ College of Chemistry and Chemical Engineering, China University of Petroleum, Qingdao 266555, China.; ^5^ Pharmaceutical Sciences Laboratory, Åbo Akademi University, 20520 Turku, Finland.; ^6^ Structural Bioinformatics Laboratory, Biochemistry, Åbo Akademi University, 20520 Turku, Finland.; ^7^ Center for Innovation Marine Drug Screening & Evaluation, Pilot National Laboratory for Marine Science and Technology (Qingdao), Qingdao 266237, China.; ^8^ Marine Biomedical Research Institute of Qingdao, Qingdao 266100, China.; ^9^ Department of Physics, Faculty of Science, King Abdulaziz University, Jeddah 21589, Saudi Arabia.; ^10^ Hangzhou No. 14 High School, Hangzhou 310000, China.; ^11^ Turku Bioscience Centre, University of Turku and Åbo Akademi University, 20520 Turku, Finland.

## Abstract

Oligonucleotide-based therapy has experienced remarkable development in the past 2 decades, but its broad applications are severely hampered by delivery vectors. Widely used viral vectors and lipid nanovectors are suffering from immune clearance after repeating usage or requiring refrigerated transportation and storage, respectively. In this work, amino-modified virus-mimetic spike silica nanoparticles (NH_2_-SSNs) were fabricated using a 1-pot surfactant-free approach with controlled spike lengths, which were demonstrated with excellent delivery performance and biosafety in nearly all cell types and mice. It indicated that NH_2_-SSNs entered cells by spike-dependent cell membrane docking and dynamin-dependent endocytosis. The positively charged spikes with proper length on the surface can facilitate the efficient encapsulation of RNAs, protect the loaded RNAs from degradation, and trigger an early endosome escape during intracellular trafficking, similarly to the cellular internalization mechanism of virions. Regarding the fantastic properties of NH_2_-SSNs in nucleic acid delivery, it revealed that nanoparticles with solid spikes on the surface would be excellent vehicles for gene therapy, presenting self-evident advantages in storage, transportation, modification, and quality control in large-scale production compared to lipid nanovectors.

## Introduction

Oligonucleotide-based therapy has experienced remarkable development in the past 2 decades, but its universal applications are severely hampered by delivery vectors. Viruses are native nanovectors with prominent advantages in gene delivery [1–6]. The evolutionary success gives viruses a set of excellent strategies to deliver DNA/RNA into cells. For example, the African swine fever virus uses a positively charged histone-like protein to encompass DNA, while severe acute respiratory syndrome coronavirus 2 uses N protein to protect RNA via a liquid–liquid phase separation strategy [7,8]. Usually, virions attach to the cell surface and activate endocytosis by hijacking cellular pathways. Interestingly, in a successful infection, viral protein may penetrate the endosomal membrane and escape from the intracellular traffic cargos. However, viral vectors will trigger immune clearance after repeated usage in the body [9]. It obviously limits the utilization of viral vectors in gene therapies, especially in small interfering RNA (siRNA)-based therapies [10,11]. Besides, the first Food and Drug Administration-approved RNA interference (RNAi) medicine (Onpattro, patisiran) was indeed a lipid-based nanoformulation, while the Moderna and Pfizer/BioNTech COVID-19 vaccines that have enjoyed great success globally are also based on lipid nanoparticles (LNP) formulations similar to that of Onpattro [12,13]. The commercial Lipofectamine 3000 (abbreviated as Lipo3000) and RNAiMAX are typical LNP-based gene delivery tools in the lab [14]. Nevertheless, LNPs are suffering from different types of obstacles that limit their broad application. The soft-state nature of LNPs is subject to chemical and physical instability for long-term use, which poses limitations both from a storage point of view as well as repeated administrations due to the immediate disintegration of the LNP upon entering the cell [15].

To overcome these issues, a virus-mimetic inorganic nanovector will take the advantage of viral vectors and avoid the above disadvantages [16]. In theory, the positively charged surface of nanovectors will efficiently encapsulate RNAs, protect loading RNAs against ribonucleases, and attach the negatively charged cell membrane tightly. Fantastically, it is possible to design an open virion-like nanovector instead of the closed native virion for gene delivery because previous studies have shown that the tough surface spike of nanovectors will facilitate their internalization. The tough spikes on the surface of nanovectors may penetrate through the cell membrane and endosomal membrane, which initiates the internalization of nanovectors and provide a convenience for their endosomal escape. Also, the densely arranged spikes on the surface may provide a solid–liquid phase separation microenvironment for RNAs as a shelter during gene delivery. A good virus-mimetic inorganic nanovector with not only the spike structure but also the positively charged surface chemistry and open porous structure can greatly take the advantage of viral vector and avoid its disadvantages. Amorphous colloidal silica are appealing materials and are widely used in biomedical applications due to their good biocompatibility, reflected by their approval by the United States Food and Drug Administration as GRAS (generally regarded as safe) materials [17]. The specific class of mesoporous silica nanoparticles (MSNs) has for instance been successfully applied as a drug delivery vector in a clinical trial [18]. Besides, researchers have found that synthetic nanoparticles with rambutan-like spike or virus-like surface nanotopography exhibit high cellular uptake and gene delivery efficacy [19–23].

It is hypothesized that the spike length plays an important role. Because of the lack of robust synthesis techniques to fabricate comparable nanoparticles with various spike lengths, it has not been possible to address this question. The surface spikes are regarded as the first touch toward cell membranes, and the cellular internalization process facilitates the engulfment of spike particles. The spike mosaic proteins (fusion proteins) in the membrane of enveloped viruses (as well as the naked forms in nonenvelope viruses) facilitate viral invasion by attaching virions to their receptors and triggering the endocytosis process. The engulfment process generates a force on the surface spikes and induces cell membrane penetration [24–26]. In this work, we invented a 1-pot synthetic method to produce silica-based nanoparticles with precisely tunable surface spike structures and spike lengths. Notably, the spikes are also silica-based, which is mechanically strong. Here, a series of virus-mimetic spiky silica nanoparticles (SSNs) with different spike lengths were successfully fabricated through a competitive epitaxial growth approach in a surfactant-free aqueous reaction system. The novel technique allows the formation of monodisperse SSNs with tunable surface spike length, as well as narrow size distribution, fine controllable spike length, and inner core diameter. It was demonstrated that the SSNs with optimized surface spike lengths showed extraordinary siRNA delivery efficacy in cells [27,28]. In vivo, the SSNs also exhibited low toxicity, suggesting the potential of SSNs as next-generation robust siRNA delivery vectors.

## Results

### Synthesis of SSNs

**Fig. 1.**
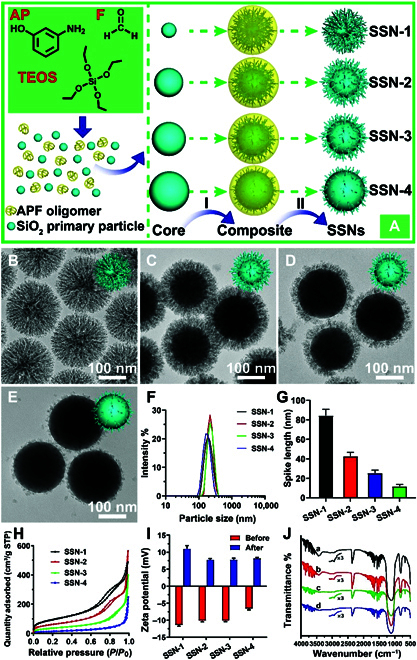
Fabrication process and structural characterizations of SSNs. Competitive self-assemble synthesis strategy and formation mechanism of SSNs (A). Self-assembly approaches employing controlled polymerization of TEOS and AP/F generate controlled cores. After continuous condensation (I), and calcination of the composites in air (II). SSNs with controlled spike length can be obtained by adjusting the process of competitive condensation kinetics. Representative TEM images of SSN-1 (B), SSN-2 (C), SSN-3 (D) and SSN-4 (E). (F) Hydrodynamic size distribution as measured by dynamic light scattering (DLS). (G) Statistical results of the length of surface spikes. (H) Nitrogen adsorption-desorption isotherms. (I) Zeta potential at neutral pH before and after amino modifications. (J) Fourier transform infrared spectrum of amino-modified SSNs. SSN-1 (A, black), SSN-2 (B, red), SSN-3 (C, green), and SSN-4 (D, blue).

The SSNs with uniform morphology were synthesized in an ethanol–water reaction system without the participation of any surfactants. Ethylenediamine was used as a cationic linker for the 2 negative polymerization systems [tetraethyl orthosilicate (TEOS) and 3-aminophenol/formaldehyde (APF) polymer] and ammonia was employed as a catalyst. During the synthesis, TEOS first hydrolyzed and condensed to form silica primary particles, which further collided to form silica core particles (Fig. 1A). Afterwards, 3-aminophenol (AP) and formaldehyde (F) began to form APF oligomer/polymer, which condensed competitively with silica primary particles on the silica core particles. The fusion protein-like silica spikes were generated by the existence of the surrounding APF polymer matrix, and the silica spike length was controlled by the delayed addition of AP and F. The initial formation process was very similar to the classic Stöber method [29,30], and the following competitive condensation process of silica primary particles and APF oligomers was used to control the growth of the surface spikes. The competitive condensation process allowed the epitaxial growth of silica spikes and the formation of SSNs after calcination of the APF polymer in air. During the synthesis, it was observed that the core size gradually increased and the spike length gradually decreased under various synthetic temperatures, 60 °C (Fig. S1A to I) or 30 °C (Fig. S1J to R), respectively. Statistical results of the surface spikes’ length on SSNs are shown in Fig. S1S. It is seen that our synthetic strategy can be used to finely control the spike length and is capable of preparing SSNs with various desired spike lengths. Moreover, by further analyzing the reaction kinetics and spike formation process, the relationship between spike length and the delayed addition time interval was proposed as shown in Fig. S1T, which could instruct the fabrication of SSNs with desired spike lengths. Among all the prepared SSNs, 4 types of representative SSNs with comparable nanoparticles size but various spikes lengths were selected, and detailed characterizations were performed to demonstrate the influences of spike length as siRNA delivery vectors.

### Characterizations of SSNs

Transmission electron microscopy (TEM) images of the obtained 4 SSNs (SSN-1, SSN-2, SSN-3, and SSN-4) clearly showed that the particles have good dispersity and uniform and comparable nanoparticle sizes (Fig. 1B to F). It could be seen that these SSNs were composed of 2 parts: (i) solid silica cores with diameter increased gradually from ~50 nm (SSN-1, Fig. 1B) to ~200 nm (SSN-4, Fig. 1E); (ii) gradually decreased spike length (Fig. 1G) from ~80 nm (SSN-1) to ~10 nm (SSN-4). The size of the silica cores and the length of the surface spikes could be tuned by simply delaying the addition of APF polymer precursors (AP and F) (see the experimental section for more details), which would reduce the reaction time of the competitive condensation process. The delayed addition of AP and F allowed a continuous consumption of TEOS in the formation of silica cores, while less TEOS (or silica primary particles) remained for the following condensation to generate silica spikes.

In order to characterize the porous nature and texture properties of these SSNs, a nitrogen sorption analysis was performed. As shown in Fig. 1H, SSN-1, SSN-2, and SSN-3 exhibited typical type IV isotherms, indicating the abundant mesopores in these nanoparticles. The hysteresis loop for SSN-4 could be barely observed, which suggested the limited mesopores in SSN-4. The prepared SSNs exhibited high specific surface areas of ~222 to 346 m^2^/g (SSN-1 to SSN-3, Table S1), except SSN-4 (53 m^2^/g), where the reduced spike length provided only limited porosity, similar to the surface area of solid nanospheres [31]. The pore size distribution of SSNs calculated with the Barrett-Joyner-Halenda method from the adsorption branch showed that it gradually increased from 6.8 nm (SSN-1) to 12.7 nm (SSN-3) and then decreased to 5.8 nm (SSN-4) (Fig. S2A, Table S1). Afterwards, SSNs were modified with amino functional groups for future applications. As shown in Fig. 1I, all SSNs exhibited negative zeta potential values at neutral pH, which changed to positive after amino modification, consistent with literature reports [32,33]. Moreover, as shown in the Fourier transform infrared spectrum (Fig. 1J and Fig. S2B), the bands observed at around 2929 and 2883 cm^−1^ further supported the successful amino group grafting on SSNs.

### siRNA delivery performance between various silica nanoparticles

**Fig. 2.**
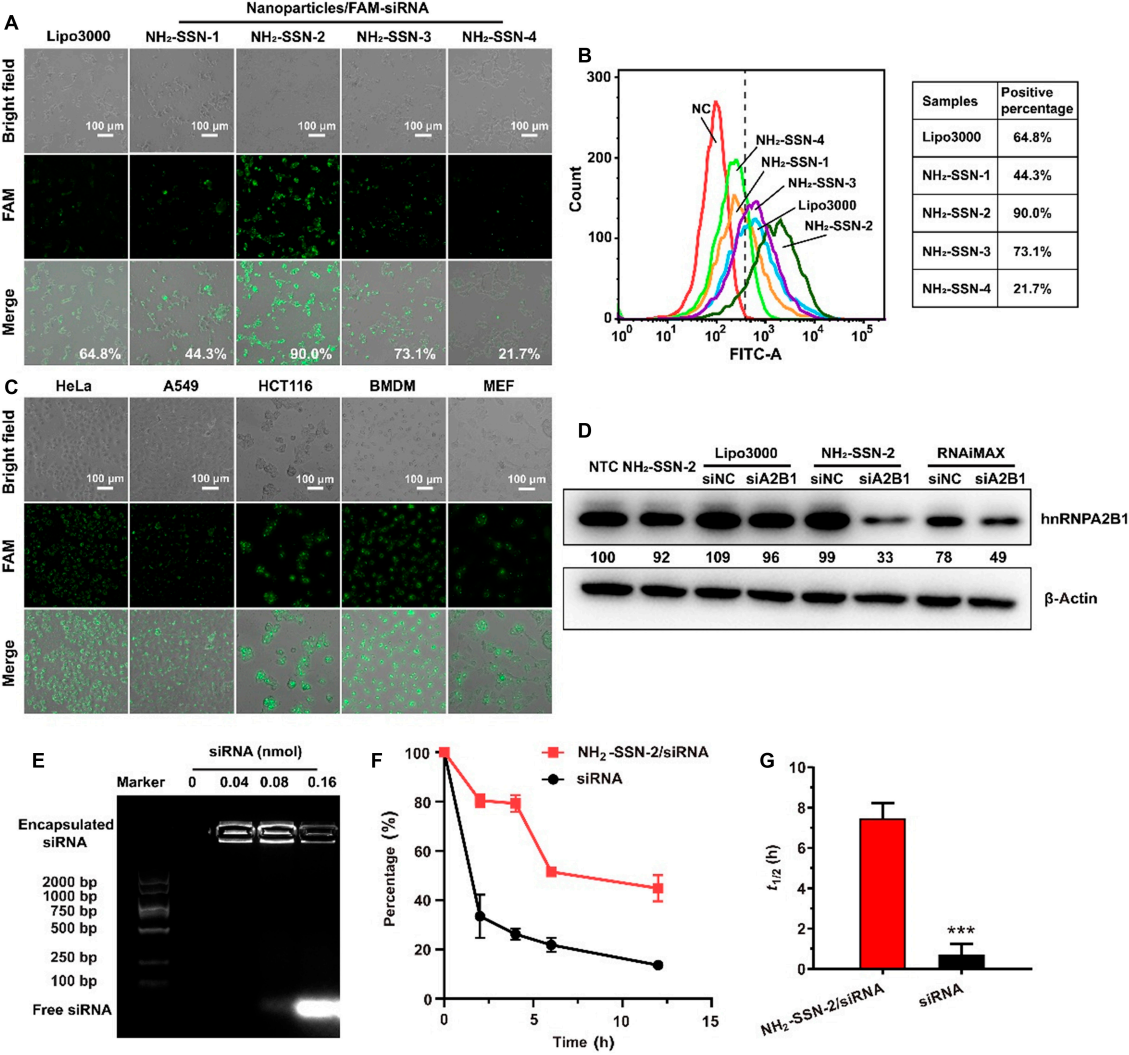
Cellular internalization of siRNA-loaded nanoparticles. SiRNA loading and protection performances. Green fluorescence-labeled siRNA (FAM-siRNA) was loaded on the amino-modified silica nanoparticles and directly added into the culture medium. (A) Fluorescence images of RNA delivery by various silica nanoparticles loaded with FAM-siRNA in HEK-293T after incubation for 6 h. (B) Flow cytometry evaluation of the delivery efficacy of NH_2_-SSNs in HEK-293T cells. (C) Fluorescence images of RNA delivery by NH_2_-SSN-2 in various cell lines after incubation for 6 h. (D) Gene knockdown by NH_2_-SSN-2-loaded siRNA. MEF cells were transfected with indicated formulations for 48 h, and 20 μg of cellular lysates was assayed by Western blotting. NTC (lane 1), bare NH_2_-SSN-2 (lane 2), Lipo3000 loaded with scramble siRNA (siNC) (lane 3) and hnRNPA2B1 siRNA (lane 4), NH_2_-SSN-2 loaded with siNC (lane 5) and hnRNPA2B1 siRNA (lane 6), RNAiMAX loaded with siNC (lane 7) and hnRNPA2B1 siRNA (lane 8). The gray intensities of hnRNPA2B1 were estimated using ImageJ and normalized to β-actin. The relative gene expression was labeled. (E) Gel-retardation assay of NH_2_-SSN-2 (100 μg) with various siRNA amounts. A constant amount of NH_2_-SSN-2 (100 μg) was mixed with various amounts (0 to 0.16 nmol) of siRNA, respectively. (F) The protection ability of NH_2_-SSN-2 on siRNA in plasma. The siRNA or NH_2_-SSN-2-loaded siRNA was added to mouse plasma at room temperature, and the siRNA degradation results were determined by gel shift assay and analyzed by ImageJ. (G) Calculated half-lives (*t*_1/2_) of the 2 formulations were analyzed through the siRNA degradation results and calculated by GraphPad Prism 8 software. All experiments were performed in triplicate. Results were analyzed by a 2-tailed *t* test and presented as means ± SD, *n* = 3. ***, *P* < 0.001.

The ultimate goal of a robust siRNA gene vector is to deliver siRNA intracellularly in various types of cells and release siRNA to initiate RNAi [34]. To highlight the critical influence of the surface spikes in siRNA delivery, conventional dendritic MSNs (DMSNs) with similar size were synthesized according to the literature and the characterizations of which were shown in Fig. S2C to F. We first screened RNA delivery abilities and cellular cytotoxicity of various amino-modified SSNs (NH_2_-SSNs) and NH_2_-DMSN (data not shown). It was found that NH_2_-SSN-2 had the best performance with the used amount of 200 μg/ml (Fig. S3A). Cells maintained high viability even with a high amount of NH_2_-SSN-2 (1 mg/ml) in primary mouse embryonic fibroblast (MEF) cells (Fig. S3B) and human embryonic kidney-293T (HEK-293T) cells (Fig. S3C), indicating its low cytotoxicity. As shown in Fig. 2A and B, NH_2_-SSN-2/FAM-siRNA-treated cells exhibited the strongest green fluorescence among all the formulations, indicating an efficient (~90.0% in HEK-293T) and successful delivery of siRNA inside the cells. Compared with Lipo3000/FAM-siRNA (~64.8%) and NH_2_-DMSN-based formulations (Fig. S3D), the surface spikes enable silica nanoparticles with strongly improved delivery performance. Importantly, higher FAM-siRNA delivery efficacy was observed for NH_2_-SSN-2 in bone marrow-derived macrophage (BMDM) cells, MEF cells, and HEK-293T cells (Fig. S3E to G) when compared with other commercial reagents (Lipofectamine RNAiMAX).

### NH_2_-SSN-2 targeted a broad range of cell types

The length of surface spikes obviously influenced the delivery performance, since low FAM-siRNA delivery efficiency was observed for longer surface spikes (NH_2_-SSN-1, 44.3%) or shorter surface spikes (73.1% for NH_2_-SSN-3 and 21.7% for NH_2_-SSN-4) other than the moderate one (NH_2_-SSN-2) in HEK-293T cells. Fluorescence intensity analysis (Fig. S4A to F) further confirmed the above observation where NH_2_-SSN-2 exhibited stronger fluorescence and higher delivery efficacy compared with other SSNs. To explore its universal nucleic acid delivery ability, NH_2_-SSN-2 was tested in all types of cells available in hand (Table S2), showing an excellent delivery capability. As shown in Fig. 2C, NH_2_-SSN-2 exhibited an extraordinary delivery performance in cultured cell lines, such as HeLa, A549, as well as the hard-to-transfect HCT116. Gene delivery to primary cells is always a challenge for any reagent-based transfection or electroporation. Thus, it was interesting to notice that NH_2_-SSN-2 allowed highly efficient delivery in murine BMDMs (nearly 100%) and primary MEF (more than 70%), which were otherwise particularly challenging for all nucleic acid delivery approaches in all labs (Fig. 2C). It indicated that the SSN with appropriate spike length (NH_2_-SSN-2) could be employed as delivery vectors to target a broad range of cell types.

Western blotting assay was conducted to semiquantitatively analyze the heterogeneous nuclear ribonucleoprotein A2/B1 (hnRNPA2B1) protein levels in the hard-to-transfected MEFs. As shown in Fig. 2D and Fig. S4G, it was not surprising to see a negligible reduction in lipofectamine-transfected MEFs because of the poor transfection efficiency. Reduced hnRNPA2B1 protein level was detected in NH_2_-SSN-2-transfected groups when compared with the nontreatment control (NTC) group, the bare nanoparticles group (NH_2_-SSN-2), and RNAiMAX-treated group. It indicated that NH_2_-SSN-2 could deliver siRNA inside the cytosol and release siRNA to realize its function. Moreover, the expression of hnRNPA2B1 protein when scramble siRNA was used and results show that there is no intrinsic effect of the formulation on hnRNPA2B1 expression. Similar results were found in other cell lines (such as A549 cells, Fig. S4H), revealing a broad target range of NH_2_-SSN-2.

### siRNA loading capability and protection performance of NH_2_-SSN-2

To explore the delivery capability of NH_2_-SSN-2 in detail, its binding affinity toward siRNA was estimated by gel retardation assay [19]. As shown in Fig. 2E, a complete electrophoretic shift can be observed for free siRNA. No release of siRNA molecules was observed even at the high amount of siRNA (0.08 nmol), indicating that NH_2_-SSN-2 effectively encapsulated siRNA. The encapsulation rate is 1.3 ± 0.1 μmol /g (siRNA per NH_2_-SSN-2). Furthermore, it demonstrated that the siRNA loading capability is highly related to the surface spike length, as shown in Fig. S5, where siRNA loading capability decreased with the decrease of surface spike length. To evaluate whether the surface spikes of NH_2_-SSN-2 can protect the loaded siRNA, fresh murine plasma was used to mimic physiological environments. As shown in Fig. 2F and G, it is interesting to observe that NH_2_-SSN-2 showed great protection of siRNA in plasma, and the elimination half-life (*t*_1/2_) of NH_2_-SSN-2 loaded siRNA (7.5 h) was significantly longer than naked siRNA (0.7 h). It is inferred that the pore size of NH_2_-SSN-2 (8.1 nm) is smaller than the size of ribonuclease (~10 nm) [35], and the siRNA molecules are hidden sterically behind the spikes, which eventually provide strong protection on the loaded siRNA molecules. It implied that NH_2_-SSN-2 could function as advanced siRNA delivery vectors with high loading capacity, robust delivery performance, and extraordinary protection performance.

### Repeated treatment of siRNA for long-term studies

Compared to shRNA, siRNA transfection is convenient but limited for long-term studies, because transfected siRNA will be diluted into daughter cells with cell proliferation. It has been demonstrated that repeated treatment of gene vectors can ensure the therapeutic effect over several entire cell proliferation cycles, especially the mitotic stage where the highest delivery efficacy can be obtained [36–38]. However, most commercial reagents would influence cell growth and proliferation during repeated treatment, even though their cytotoxicity is almost negligible in a single usage. Therefore, the advanced delivery property of NH_2_-SSN-2 was shown in a repeated treatment assay for up to 8 d in MEF cells, which are very sensitive to cytotoxicity.

**Fig. 3.**
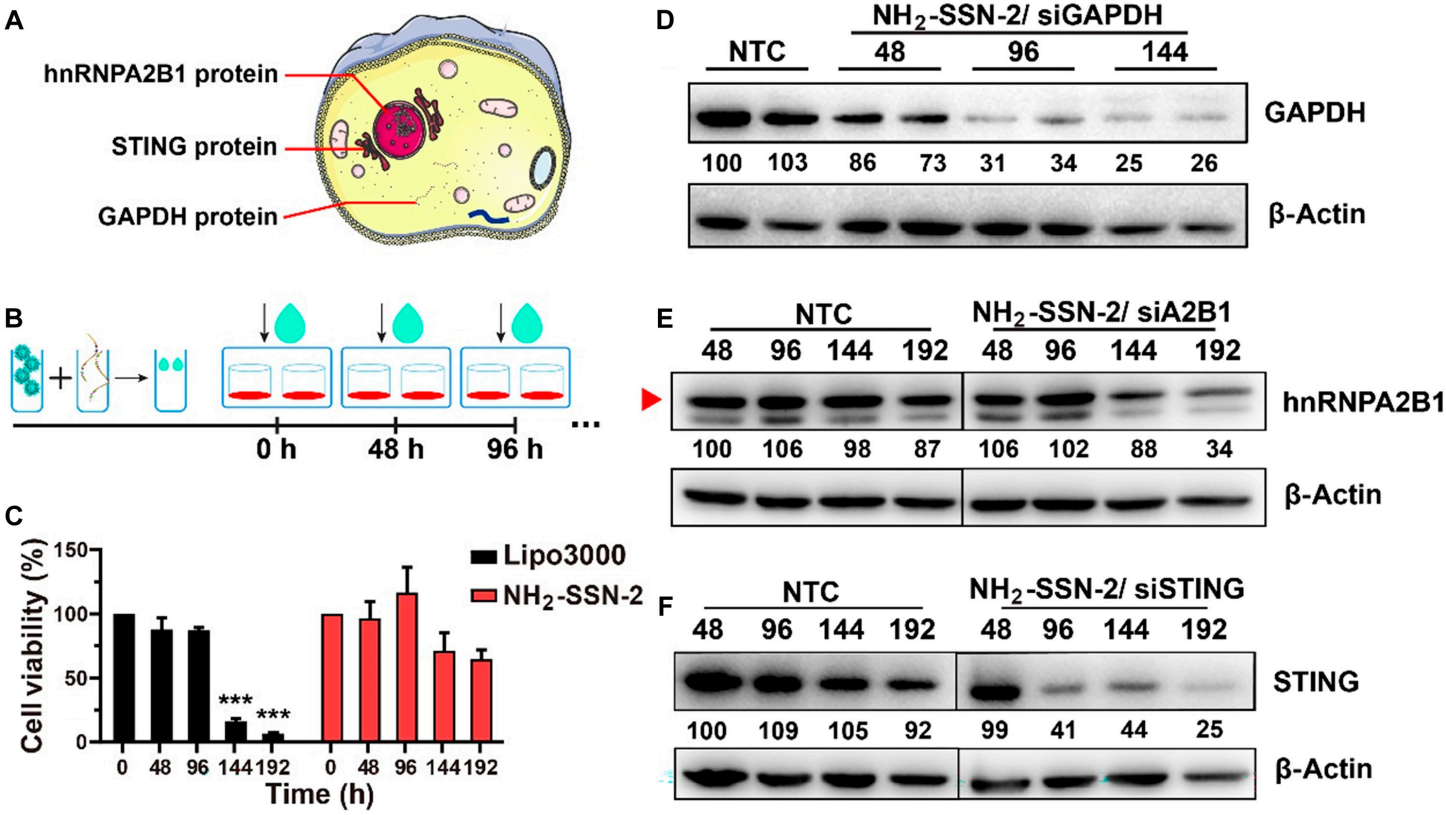
Repeated treatment of siRNA. (A) Subcellular locations of 3 indicated genes. (B) Schematic illustration of the repeated treatment. (C) Cell viability of MEF cells after repeated treatment. Results were presented as means ± SD and analyzed by 1-way ANOVA. ***, *P* < 0.001, *n* = 3. (D to F) Reduced protein levels in MEF cells after repeated treatment. Twenty micrograms of cellular lysates was assayed by Western blotting using indicated antibodies. Experiments were repeated at least 3 times. The gray intensities of GAPDH, hnRNPA2B1, and STING were estimated using ImageJ and normalized to β-actin. The relative gene expression was labeled.

As a demonstration, hnRNPA2B1 in the nucleus, stimulator of interferon genes (STING) on the endoplasmic reticulum, and the house-keeping gene glyceraldehyde-3-phosphate dehydrogenase (GAPDH) in the cytoplasm were selected as the targeting gene (Fig. 3A). Noticeably, GAPDH is an excellent candidate in Western blotting analyses as an internal loading control due to its stable expression in almost all tissues [39]. Transfection complexes were added into the cell culture medium every 48 h (Fig. 3B). Marked cell viability reduction appeared in Lipo3000 incubated groups after 144-h treatment (Fig. 3C), indicating the cytotoxicity of Lipo3000 during repeated treatment. As shown in Fig. 3D, a reduction of GAPDH protein was detected in both groups 48 h post-transfection. With repeated treatment, it could be seen that a gradual knockdown of GAPDH protein was achieved. Only a limited amount of GAPDH protein can be detected after 3 repeated transfections. It demonstrated the feasibility of NH_2_-SSN-2 as a repeated treatment agent to realize sustained gene knockdown. Similar observations were found for hnRNPA2B1 and STING genes (Fig. 3E and F). Moreover, the knockdown performance was statistically analyzed by measuring the gray values of the Western blotting results, which further supported the above observations (Fig. S6).

### Cellular internalization of NH_2_-SSN-2

**Fig. 4.**
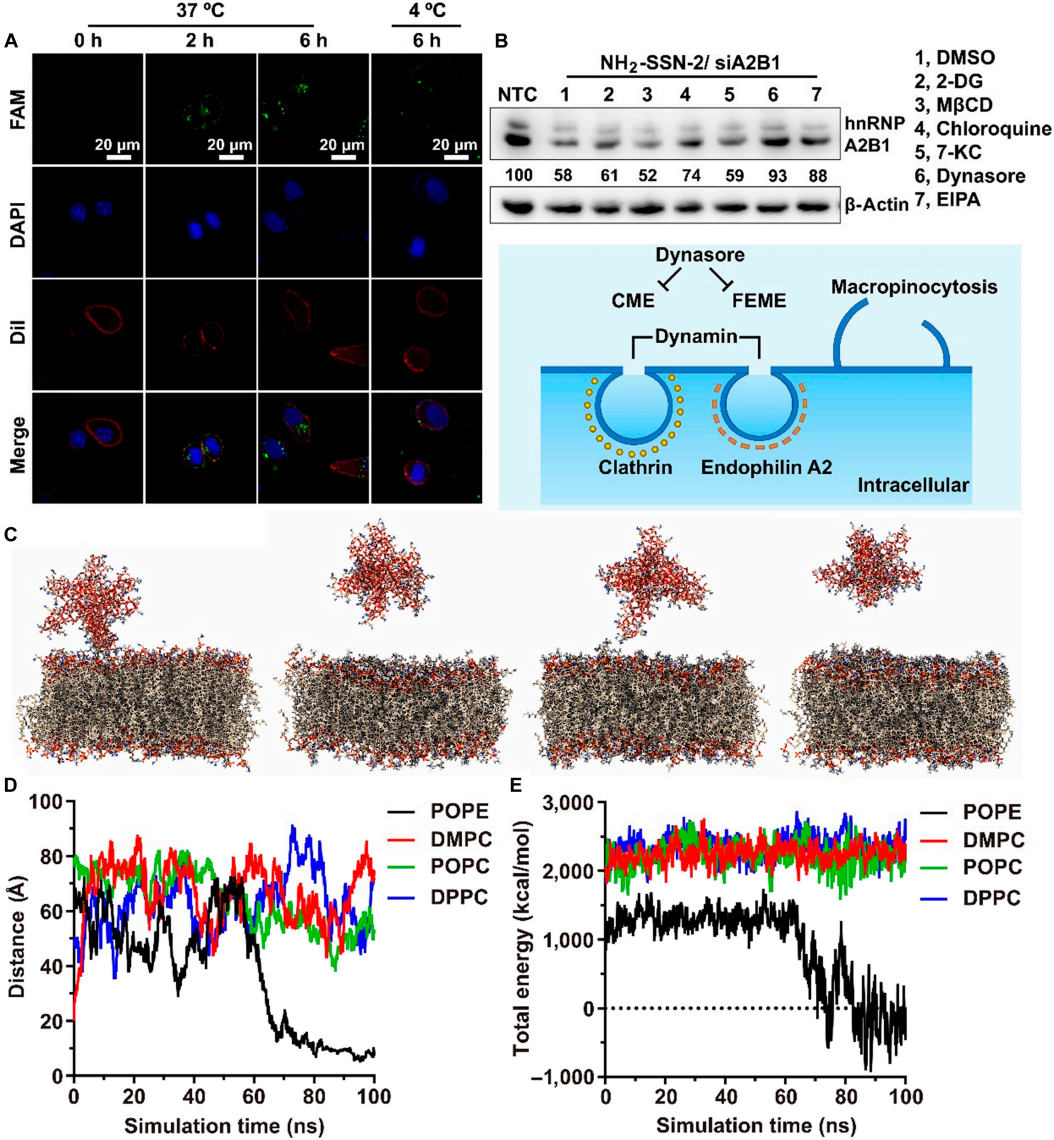
Cellular internalization behavior. (A) Time-dependent fluorescence images of FAM-siRNA delivery by NH_2_-SSN-2 in MEF cells after incubation at 37 °C/4 °C for 0 to 6 h. The cell membrane was stained with DiI (red). (B) In vitro determination of relative hnRNPA2B1 and β-actin protein levels in cells transfected with NH_2_-SSN-2 in the presence of various internalization inhibitors. Twenty micrograms of cellular lysates were assayed by Western blotting. The gray intensities of hnRNPA2B were estimated using ImageJ and normalized to β-actin. The relative gene expression was labeled. Experiments were repeated at least 3 times. 2-DG (pan-inhibitor of energy-dependent endocytosis), MβCD (inhibitor of lipid rafts/cholesterol-enriched microdomains/caveolae pathway), Chloroquine (CME inhibitor), 7-KC (CLIC/GEEC inhibitor), dynasore (CME and FEME inhibitor), EIPA (macropinocytosis inhibitor). (C) Interaction of spike silica nanoparticles (SSN-2) with different lipid bilayers; (from left) SSN-2-POPE, SSN-2-DMPC, SSN-2-POPC, SSN-2-DPPC after 100-ns MD simulations (SSN-2 and lipids are shown in elemental ball-and-stick representation, Na^+^ and Cl^−^ ions, and water molecules are hidden for clarity). (D) The distance between the silica atom (Si973) on the tip of the SSN-2 spike to the closest lipid C2 atom in the upper leaflet of the lipid bilayer during the 100-ns MD simulation. (E) The total interaction energy was calculated as the sum of electrostatic and van der Waals interactions between the SSN-2 and the studied lipid membranes during the 100-ns MD simulation.

To gain detailed cellular internalization information for NH_2_-SSN-2, a time-course analysis was performed to conduct the cellular uptake of nanoparticles. As shown in Fig. 4A, cells started to internalize NH_2_-SSN-2/FAM-siRNA complexes as early as 2 h post-incubation. In the meantime, the loaded FAM-siRNA started to release from NH_2_-SSN-2 as pointed out by arrows in the image. A large amount of FAM-siRNA could be successfully delivered inside the cytosol after ~4 to 6 h of incubation, demonstrating the efficient internalization performance of NH_2_-SSN-2 (other incubation time intervals in Fig. S7). Nearly no green fluorescence was found in cells at 4 °C even after incubation for ~6 h, indicating that the cellular uptake was an energy-dependent process, e.g., endocytosis, rather than passive diffusion [40–43].

A consensus is currently developing for 5 major types of endocytosis: clathrin-coated pit-mediated endocytosis (CME, clathrin and dynamin-dependent), fast endophilin-mediated endocytosis (FEME, clathrin-independent but dynamin-dependent), clathrin-independent carrier (CLIC)/glycosylphosphatidylinositol-anchored protein enriched early endocytic compartment (GEEC) endocytosis (clathrin and dynamin independent), micropinocytosis, and phagocytosis [44]. In the presence of various internalization inhibitors, representative fluorescence images (Fig. S8A) and the quantitative measurement of intracellular amounts of FAM-siRNA (Fig. S8B) demonstrated the cellular uptake of Rhodamine B isothiocyanate (RITC)-labeled NH_2_-SSN-2, and the successful delivery of FAM-siRNA, with no marked toxic effects, were observed using the inhibitors under selected treated amounts (Fig. S8C). The corresponding hnRNPA2B1 knockdown performance was shown in Fig. 4B. It was noted that hnRNPA2B1 protein was significantly reduced under the treatment by siRNA-loaded NH_2_-SSN-2, whereas it was rescued by dynasore/EIPA but not by other internalization inhibitor treatments, indicating a dynamin-dependent pathway [44].

**Fig. 5.**
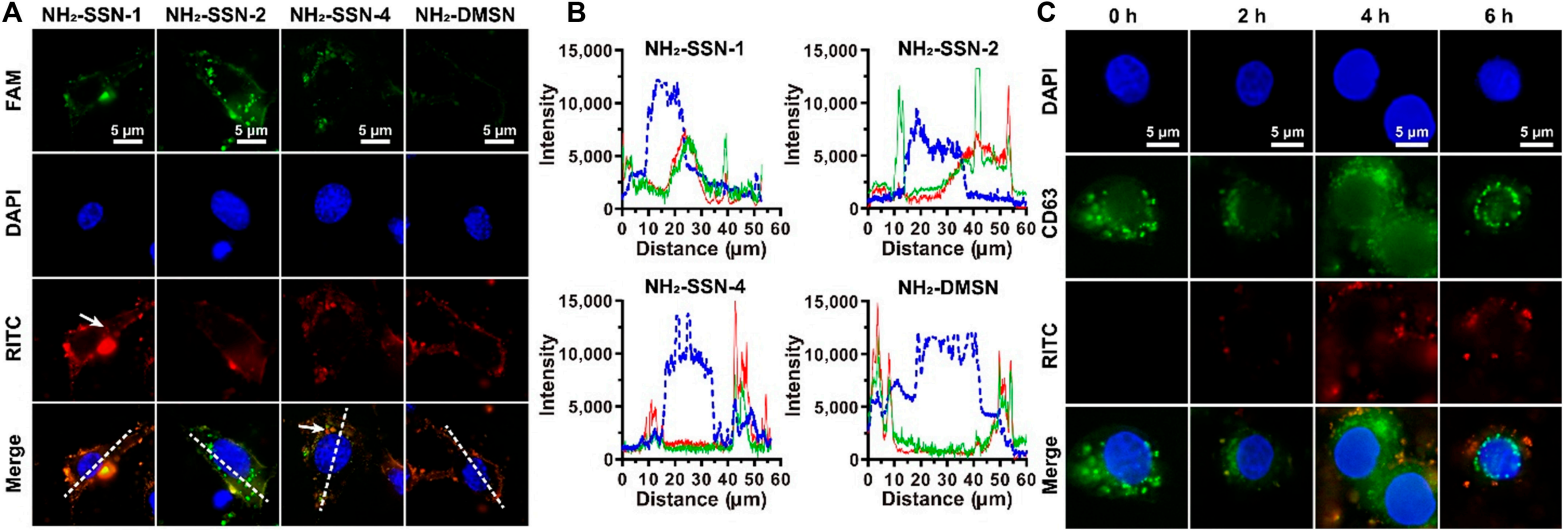
Endosomal escape. The nanoparticles and siRNA were labeled with RITC (red) and FAM (green), respectively. The nucleus was stained with DAPI (blue). (A) Fluorescence images of FAM-siRNA delivery by NH_2_-SSN-1 (long surface spikes), NH_2_-SSN-2 (moderate surface spikes), NH_2_-SSN-4 (short surface spikes), and no surface spike NH_2_-DMSN in MEF cells. (B) Distribution of the 3 fluorescence along the dashed lines in the merged images from (A). Nanoparticle (red), RNA (green), and nucleus (blue). (C) Cellular internalization of NH_2_-SSN-2 in MEFs.CD63 is a biomarker of late endosomes/lysosomes.

To study how the amino-modified SSN-2 interacts with lipid membranes, we carried out molecular dynamics (MD) simulations of 4 NH_2_-SSN-2-membrane systems using predefined single-lipid-component membranes, i.e., 1-palmitoyl-2-oleoyl-sn-glycero-3-phosphatidylethanolamine (POPE), 1,2-dioleoyl-sn-glycero-3-phosphatidylcholine (DMPC), 1-palmitoyl-2-oleoyl-sn-glycero-3-phosphatidylcholine (POPC), and 1,2-dipalmitoyl-sn-glycero-3-phosphocholine (DPPC) (Fig. 4C to E). It was shown that, even with POPE lipid bilayer, there was no apparent fusion of the SSN-2 with membranes, suggesting that an active transport mechanism would be needed to facilitate the entry of the nanoparticle (more details could be found in Fig. S9). Together with the unsuccessful internalization at 4 °C (Fig. 5A) and the successful inhibition by internalization inhibitors, it clearly indicated that NH_2_-SSN-2 entered cells via energy- and dynamin-dependent manner.

### Cytosol release of NH_2_-SSN-2-loaded siRNA

To further explain the high nucleic acid delivery performance of NH_2_-SSN-2 in cells, the subcellular localization of FITC-labeled nanoparticles and FAM-siRNA were examined in MEF cells (Fig. 5A and B). After 6-h incubation, some of the FAM-siRNA/nanoparticles formed a cycle around the cell surface, suggesting these formulations could first adhere to the cell surface, while some of the delivery vectors were observed internalized into the cytosol for SSNs-based delivery vectors. However, no green signals were found in NH_2_-DMSN-treated cells, implying the failure of the cellular uptake of DMSN (Fig. 5A, lane 4). Merged peaks in the curve and colocalized green and red dots were found in NH_2_-SSN-1-treated cells, suggesting that FAM-siRNA failed to dissociate from NH_2_-SSN-1 (Fig. 5A, lane 1). Surprisingly, abundant green dots and diffused red fluorescence were found in NH_2_-SSN-2-treated cells, indicating a successful siRNA release from nanocarriers (Fig. 5A, lane 2). Although siRNAs could easily be released from NH_2_-SSN-4 (Fig. 5A, lane 3), the nucleic acid delivery efficiency was poor because it could not load enough siRNAs (Fig. S5). It suggested that the surface spike length was related to siRNA loading abilities and siRNA release. It demonstrated that the high nucleic acid delivery performance of NH_2_-SSN-2 was related to its high cellular internalization capability and siRNA release. The separated subcellular localization of NH_2_-SSN-2 and late endosomes/lysosomes marker (CD63) indicated successful endosomal escape (Fig. 5C), which avoided the rapid degradation of RNAs in cells. The release of siRNA from NH_2_-SSN-2 is further semiquantitatively analyzed by measuring the percentage of colocalization/ separated red and green signals in over 20 cells (Fig. S10). It is shown that FAM-siRNA starts to release from the late endosomes/lysosomes after 2 h of incubation and has almost totally released after 6 h of incubation.

### Gene delivery via NH_2_-SSN-2 in vivo

**Fig. 6.**
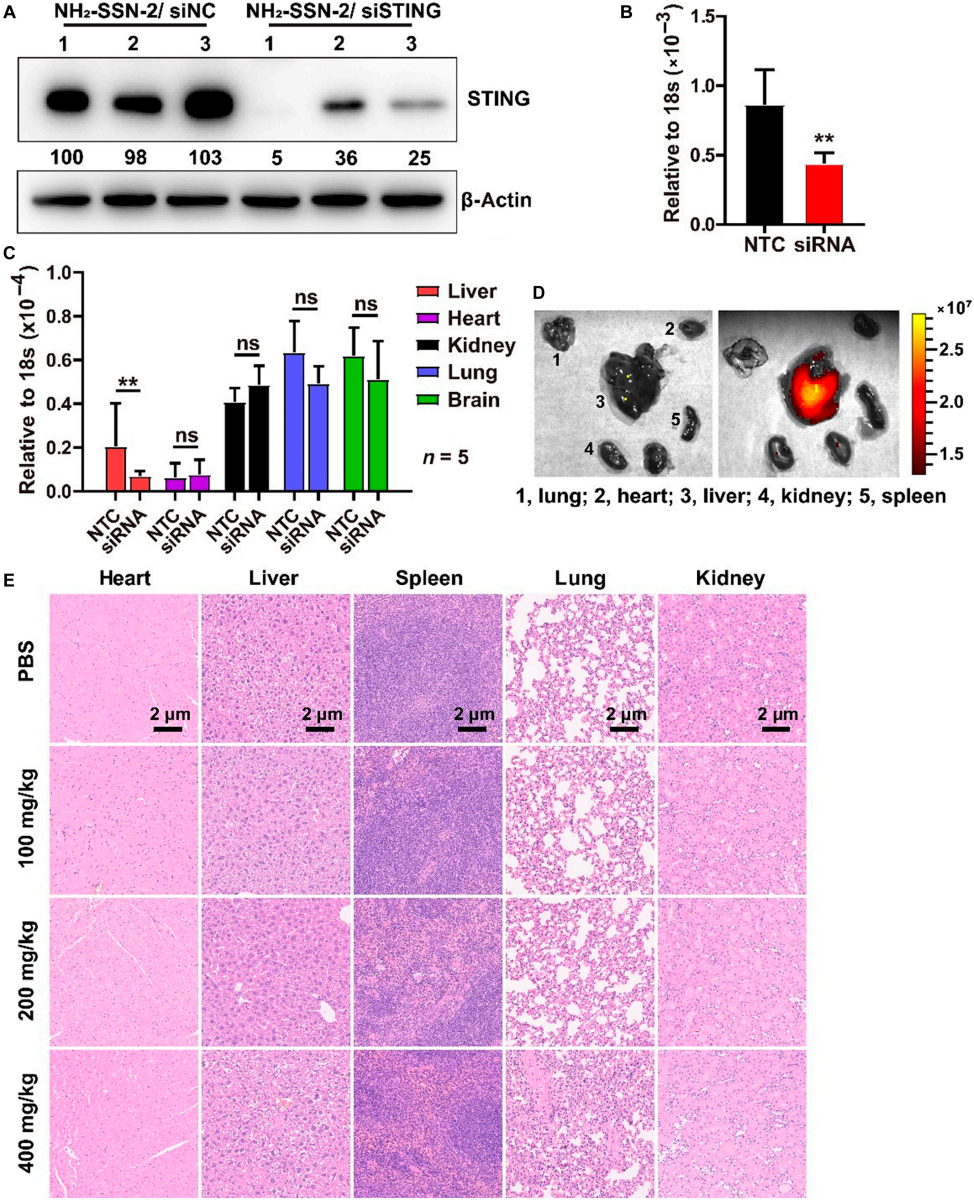
In vivo siRNA delivery performances. (A) STING and β-actin protein levels in white blood cells from 3 individuals. NH_2_-SSN-2 loaded siRNA formulation was intravenously injected. White blood cells were collected from each mouse at 6 h post-injection. Forty micrograms of cellular lysates was assayed by Western blotting. siNC, nontarget control siRNA. The gray intensities of GAPDH, hnRNPA2B1, and STING were estimated using ImageJ and normalized to β-actin. The relative gene expression was labeled. (B) In vivo knockdown efficacy of STING gene by NH_2_-SSN-2-based formulation with comparison to NTC. Results were presented as means ± SD and analyzed by a 2-tailed *t* test. **, *P* < 0.01, *n* = 3. (C) In vivo knockdown efficacy of STING gene in various organs. Results were presented as means ± SD and analyzed by 2-tailed *t* test, **, *P* < 0.01; ns, not significant; *n* = 5. (D) Fluorescence images showing the targeting property of NH_2_-SSN-2 toward major organs in mice. (E) Representative results for H&E staining in major organs from mice treated with various doses of NH_2_-SSN-2.

Given the excellent protection ability in murine plasma, we believed that NH_2_-SSN-2 could silence gene expression in vivo. We tested NH_2_-SSN-2/FAM-siRNA complexes in mice. As shown in Fig. S11, a strong increase of the FAM green fluorescence was observed after the NH_2_-SSN-2/FAM-siRNA complexes injection. To further estimate the in vivo silencing efficiency of NH_2_-SSN-2 delivered siRNA, we evaluated the STING gene knockdown in mice. STING plays a vital role in immunity and inhibiting STING may be potentially therapeutic for inflammatory treatments [45,46]. As shown in Fig. 6A to C, STING protein and mRNA were reduced in white blood cells. Afterwards, the STING mRNA in the various organs was assayed after perfusing by phosphate-buffered saline (PBS). It is observed that the NH_2_-SSN-2/STING-siRNA complex exhibited a certain degree of accumulation in the liver. Fluorescence images (Fig. 6D) further confirmed the liver accumulation of NH_2_-SSN-2. We also estimated the toxicity of NH_2_-SSN-2 by assessing acute toxicity in vivo. It demonstrated no marked difference between PBS and NH_2_-SSN-2 treatment groups within 2 weeks after injection. No mice died and no obvious weight loss was observed even though the NH_2_-SSN-2 dose was at 400 mg/kg (Fig. S12), which was 20 times higher than its effective knock-down dose in vivo. The morphology and hematoxylin-eosin (H&E) staining showed that NH_2_-SSN-2 nanocarriers induced no necrosis or apoptosis in major organs under 200 mg/kg (Fig. 6E). However, marked tissue edema and infiltration of inflammatory cells appeared in the lung at a dose of 400 mg/kg. It suggested that NH_2_-SSN-2 was an efficient delivery agent and promising for future delivery of siRNA in vivo.

## Discussion

In summary, a series of SSNs have been successfully fabricated through a 1-pot surfactant-free competitive epitaxial growth approach. The obtained nanoparticles exhibit uniform particle size (~200 nm) and, most importantly, easily tunable spike lengths. It is first demonstrated that the length of surface spikes can greatly influence siRNA loading and release performance, and longer surface spikes have higher loading and less release of siRNA. Taking advantage of the tunable spike lengths of SSNs, we have demonstrated that amino-modified SSNs with appropriate spike lengths (~40 nm, NH_2_-SSN-2) exhibit an extraordinary siRNA protection capability and siRNA delivery performance both in vitro and in vivo.

Our studies provided new evidence that the proposed synthetic strategy can efficiently modulate the surface spike structure of silica nanoparticles. Though previous research reported the synthesis of silica nanoparticles with spikes on the surface [19,20,23], fine control of the spike length remains a challenge due to the lack of efficient strategies. Our proposed fabrication strategy can easily control the relative condensation kinetics of the reactants by controlling the precursors’ adding sequence and delay addition time interval, which eventually controls the length of surface spikes. For previous studies, the spikes are modulated by reaction time, where the spikes grow very fast at first and difficult to control their spike lengths. Moreover, benefiting from the developed new approaches, it is possible to prepare silica nanoparticles with the controlled length of surface spikes, and it is revealed that the length of surface spikes has a substantial impact on RNA delivery efficacy and surface spikes with certain lengths have the best RNA delivery efficacy. Besides, understanding how these nanoparticles are internalized by cells and then processed within the cells is critical for a delivery system. We explored the cellular uptake pathway of NH_2_-SSN-2 via computer simulation and biological approaches. It was found that NH_2_-SSN-2 was internalized through dynamin-dependent endocytosis (Fig. 5), which provides the theoretical basis for the subsequent development of new delivery systems. We believe that this study provides an important new step toward the application of siRNAs as therapeutic agents by providing a new delivery platform with enhanced delivery performance, especially in vivo. Our results indicate that NH_2_-SSN-2 loaded with siRNA has good clinical translation potential with good stability and biosafety.

## Materials and Methods

### Cell culture and reagents

HeLa, HepG2, and 293T cells were cultured in Dulbecco's modified Eagle medium (DMEM). B16F10 cells were cultured in RPMI 1640. All culture mediums were supplemented with 10% (v/v) fetal bovine serum (FBS), 5 μg/ml of penicillin and 10 μg/ml of streptomycin. BMDMs were generated as described [1]. Briefly, bone marrow from the tibia and femur was flushed out by PBS and cultured in 10 ml of complete medium (DMEM supplemented with 20% heat-inactivated FBS and 20 ng/ml granulocyte-macrophage colony-stimulating factor) at 37 °C for 7 d. MEFs were harvested according to the standard protocol [2]. Briefly, the mouse whole embryo (E13.5) was isolated. Heads and viscera were removed. The remaining bodies were washed in PBS and transferred to 35-mm petri dishes, minced with scissors, and digested with 0.25% trypsin/EDTA (1 mM) for 5 to 10 min at 37 °C. Following digestion, 1 to 3 ml of DMEM, supplemented with 10% FBS and 5 μg/ml of penicillin and 10 μg/ml of streptomycin, were added. The tissue was pipetted up and down to get a single-cell suspension. All cells were maintained in an incubator with a humidified atmosphere of 5% CO_2_ at 37 °C.

Primary antibody hnRNPA2B1 (sc-374053) was purchased from Santa Cruz Biotechnology. The STING (#13647), CD63 (#55051), β-actin (#4970), and GAPDH (#5174) antibodies were purchased from Cell Signaling Technology (Denver, MA, USA). The secondary antibody goat anti-rabbit (111-035-003) and goat anti-mouse (115-035-003) were purchased from Jackson ImmunoResearch (West Grove, PA, USA). The fluorescence secondary antibody, Alexa Fluor plus 488 (A-11008), the Lipofectamine 3000, and RNAiMAX were purchased from Thermo Fisher Scientific (Carlsbad, CA, USA). The 2-deoxy-d-glucose (2DG), chloroquine, dynasore, Methyl-β-cyclodextrin (MβCD), cytochalasin dand amiloride (EIPA) were purchased from Titan Scientific Co., Ltd (Shanghai, China). The 7-keto-cholesterol (7-KC) was purchased from Tsbiochem Scientific Co., Ltd (Shanghai, China). The Resazurin cell viability kit was purchased from Thermo Fisher Scientific (Carlsbad, CA, USA). VECTASHIELD antifade mounting medium with 4',6-diamidino-2-phenylindole (DAPI) was purchased from Vector Laboratories (Burlingame, CA, USA). The cell plasma membrane staining kit with DiI (1,1'-dioctadecyl-3,3,3',3'-tetramethylindocarbocyanine perchlorate) was purchased from Beyotime Biotechnology (Shanghai, China). All kits were used following the manufacturer's instructions.

The following primer pairs were used in real-time quantitative polymerase chain reaction (PCR) as listed. For mouse STING gene, 5′-TCAGTGGTGCAGGGAGCCGA-3′ (F) and 5′-CGCCTGCTGGCTGTCCGTTC-3′ (R); for mouse 18S ribosomal RNA gene, 5′-ATTGACGGAAGGGCACCACCAG-3′ (F) and 5′-CAAATCGCTCCACCAACTAAGAACG-3′ (R). The siRNA sequences used in this study are listed as follows. 5′-CCACAGAAGAAAGTTTGAGTT-3′ for mouse hnRNPA2B1. 5′-CTTTGGTGGTAGCAGGAAC-3′ for human hnRNPA2B1. 5′-CCAACAGCGUCUACGATT-3′ for mouse STING. 5′-UGACCUCAACUACAUGGUUTT-3′ for GAPDH. The nonsense sequence 5′-TTCTCCGAACGTGTCACGT-3′ was used as a nontarget siRNA. For FAM-labeled siRNA (FAM-siRNA), FAM was conjugated with nonsense RNA at the 3′ end. siRNA targeting enhanced green fluorescent protein is a kind gift from Dr. Lin.

### The synthesis of SSNs with various spike lengths

SSN-1 was synthesized via a facile 1-pot self-assembly co-condensation process. In a typical synthesis, 2 ml of TEOS was added to an aqueous solution composed of ethanol, distilled water, ammonium hydroxide, and ethylenediamine. Afterwards, AP and formaldehyde F solution were added to the above solution. After reaction for 5 h, the product was harvested by centrifugation. Finally, SSN-1 was obtained after calcination in air. For the synthesis of SSNs with various spike lengths, TEOS was firstly added to the aqueous solution. After various time intervals (5 min for SSN-2, 10 min for SSN-3, and 20 min for SSN-4), AP and F were then added to the reaction solution. After centrifugation, drying, and calcination in air, SSN-2, SSN-3, and SSN-4 could be prepared.

### The synthesis of large pore DMSNs

DMSNs were prepared according to the reported literature with minor modifications [3,4]. In a typical synthesis, 0.14 g of triethanolamine was added to 50 ml of water, which was stirred at 80 °C in an oil bath for 15 min. Afterwards, 336 mg of sodium salicylate (NaSal) and 760 mg of cetyltrimethylammonium bromide was added to the above solution. The solution was continuously stirred for another h. Then, 5 ml of TEOS was added to the above solution and DMSNs were harvested by centrifugation after 2 h of reaction. Finally, the DMSNs were calcined at 550 °C in air to remove the surfactants.

### Grafting of amino group on silica nanoparticles

Four milligrams of silica nanoparticles were dissolved in 1 ml of ethanol solution and sonicated for 15 min. Afterwards, (3-aminopropyl) triethoxysilane was added to the above ethanol solution and stirred for 24 h. The amino-modified silica nanoparticles were collected by centrifugation and washed with ethanol several times. The amino-modified silica nanoparticles were named as NH_2_-SSN-1, NH_2_-SSN-2, NH_2_-SSN-3, NH_2_-SSN-4, and NH_2_-DMSN for SSN-1, SSN-2, SSN-3, SSN-4, and DMSNs, respectively.

### Theoretical calculation of the surface spike length of SSNs

The relationship between the surface spike length and the delay addition time interval of AP and F solution were theoretically calculated for the synthesis. During the theoretical calculation, we made the following assumptions: (1) TEOS were added to the reaction solution at the time point of “0”, AP and F solution was added to the reaction solution at the time point of “t”. (2) In total, the number of nanoparticles yielded in the reaction solution was “N” regardless of the delay addition time interval and it kept consistent during the synthesis. (3) During the reaction, the silica spikes grow vertically on the surface of the in situ generated silica cores and the structure of the spikes was simplified as uniform cylinder structures. On the basis of the above assumptions, the relationship between the total amount of Si (M) and the consumed amount (m (t)) to generate silica cores are calculated by rate reaction equation, which can be written as:

$$\left\{\begin{array}{c} \frac{\mathrm{dm}\left(t\right)}{\mathrm{dt}}=k\left(M-m\left(t\right)\right)\\ m\left(0\right)=0 \end{array}\right.$$ (1)

*m* (*t*) can be derived from Eq. 1 as:

$$m\left(t\right)=M\left(1-e^{-\mathrm{kt}}\right)$$ (2)

where k is a constant.

The radius of the silica core particles are “R” with the material density of “*ρ*”, therefore,

$$m\left(t\right)=N\cdot \frac{4\pi }{3}R^{3}\rho$$ (4)

This yields for the average radius of the nanoparticles:

$$R={\left(\frac{3m\left(t\right)}{4\mathrm{\pi \rho N}}\right)}^{\frac{1}{3}}$$ (4)

Since the total Si amount in all the spikes is:

$$M\left(s\right)=M-m\left(t\right)$$ (5)

The number of spikes in each nanoparticle is calculated as:

$$N\left(s\right)=N\cdot \frac{4\pi R^{2}}{\mu }$$ (6)

Where *μ* is the distribution density of spikes on the core surface.

Furthermore, the mass of each spike is calculated as:

$$m\left(s\right)=\frac{M\left(s\right)}{N\left(s\right)}=\frac{M-m\left(t\right)}{\frac{N\cdot 4\pi R^{2}}{\mu }}$$ (7)

Afterwards, the length of spikes can be calculated from Eq. 7 as:

$$l\left(t\right)=\gamma \cdot \frac{M-m\left(t\right)}{\frac{N\cdot 4\pi R^{2}}{\mu }}=\frac{\gamma \mu \left(M-m\left(t\right)\right)}{N\cdot 4\pi R^{2}}$$ (8)

Where *γ* is the ratio between the mass and length of a spike.

Furthermore, the length of the surface spikes can be calculated as:

$$l\left(t\right)=C\cdot \frac{e^{-\mathrm{kt}}}{{\left(1-e^{-\mathrm{kt}}\right)}^{\frac{2}{3}}}$$ (9)

Where *C* is a constant, and $C=\frac{\gamma \mu }{4\mathrm{\pi N}{\left(\frac{3M}{4\mathrm{\pi \rho N}}\right)}^{\frac{2}{3}}}$,

With input the measured data from Fig. S1, *C* is calculated to be 1.522, and *k* is calculated to be 0.003157. Therefore, the length of the surface spikes can be calculated as:

$$l\left(t\right)=1.522\cdot \frac{e^{-0.003157t}}{{\left(1-e^{-0.003157t}\right)}^{\frac{2}{3}}},\;0\leq t\leq 60\;\min$$ (10)

### Characterizations of SSNs

TEM measurements were conducted on a JEOL-1010 (Tokyo, Japan) microscope. Micromeritics Tristar 3000 system was used to characterize the nitrogen adsorption/desorption experiments at 77 K. Before the nitrogen adsorption/desorption measurements, samples were pretreated under a vacuum line at 120 °C for 12 h. Specific surface areas were calculated by the Brunauer-Emmett-Teller method through the adsorption data at relative pressure (*P*/*P*_0_) range of 0.05 to 0.35. Pore-size distribution curves and the total pore volumes of the samples were obtained by the Barrett-Joyner-Halenda method through the adsorption branches of the isotherms. Furthermore, the total pore volume was calculated from the amount of nitrogen adsorbed at the relative pressure (*P*/*P*_0_) of 0.99.

### Cell transfection

NH_2_-SSNs were dissolved in diethylpyrocarbonate-treated water at the final concentration of 10 mg/ml. In separate tubes, 20 μl of dissolved NH_2_-SSNs and 100 pmol of siRNA were diluted in 100 μl of serum-free medium, respectively. The diluted siRNA was added to the diluted NH_2_-SSNs and incubated at room temperature for 15 min. Then, 200 μl of mixture was added to the culture medium, respectively. The commercial siRNA transfection reagent was used following the manufacturer’s instructions.

For repeated treatment, MEF and 293T cells were transfected with NH_2_-SSN-2/siRNA, Lipo3000/siRNA, and RNAiMAX/siRNA, respectively. Afterwards, transfected cells were cultured and divided into 3 equal parts every 48 h. One part was repeatedly transfected. The left 2 parts were used to evaluate gene knockdown efficiency and cell viability. For cell viability assay, cells were incubated for 4 h with 1 mg/ml resazurin solution (10 μl), and then the fluorescence intensity was measured using a SpectraMax M3 microplate reader (Molecular Devices, San Jose, CA, USA) at an excitation wavelength of 544 nm and emission wavelength of 595 nm [5].

To estimate gene delivery efficiency, transfected cells were evaluated with flow cytometry analysis performed by a BD FACSAria II flow cytometer (BD Biosciences, San Diego, CA, USA). A total of 10^4^ gated events were acquired per sample. Data were analyzed with FlowJo_V10 software (10.5.4).

### Microscopy assay

MEF, HeLa, HepG2, 293T, and BMDM cells were seeded in 6-well plates (1 × 10^5^ cells per well) for 12 h. A concentration of 100 μg/ml NH_2_-SSNs/FAM-siRNA was added and incubated with the cells for 0, 2, and 6 h, respectively. After washing 3 times to remove nonbinding particles, cells were fixed with 4% paraformaldehyde and visualized on ZEISS Vert.A1 fluorescence microscope (Carl Zeiss, Heidenheim, Germany).

### Immunofluorometric assay

MEF and HepG2 cells were grown on coverslips for 12 h. After treatment with NH_2_-SSNs, cells were washed with PBS and fixed with 4% paraformaldehyde. The cells were permeabilized in 0.5% v/v Triton X-100 in PBS and blocked by 5% bovine serum in PBS. Then, the cells were incubated with the primary antibodies (1:100) overnight and incubated with secondary antibodies (1:2000) for 60 min. After being sealed with the VECTASHIELD mounting medium with DAPI cells were visualized on the Leica TCS SP8 STED confocal microscope (Leica Microsystems, Weztlar, Germany). Images were analyzed by ImageJ and ZEN Imaging Software.

### RT-PCR and quantitative PCR analysis

Total RNA was isolated using TRIzol reagent (Takara, Japan) according to the manufacturer’s instructions. One microgram of total RNA was converted into complementary DNA with random primers and Superscript III reverse transcriptase (Takara, Japan). PCR was performed with gene-specific primer sets. Quantitative real-time PCR was performed with SYBR green (Roche, Switzerland) incorporation on the LightCycler 96 System (Roche, Switzerland). 18S ribosomal RNA was used as internal control, and the data were presented as accumulation index (2^−△△Ct^) [6].

### Gel shift assay

NH_2_-SSNs (100 μg) were mixed with the indicated amount of siRNA. The mixture was incubated for 30 min at room temperature, mixed with loading dye, and submitted to gel shift assay. The siRNA binding ability of NH_2_-SSNs was measured by gel shift assay as previously described. The polymer/siRNA ratios were electrophoresed in a 2% agarose gel containing GeneGreen at 35 V in the tris-acetate-ethylenediamine tetraacetic acid solution [7]. For the protection ability analysis, peripheral blood was isolated from healthy C57BL/6 mice and centrifuged at 12000×g for 5 min to collect plasma. The naked siRNA or siRNA/NH_2_-SSN-2 mixture was incubated with plasma at 37 °C and then submitted for gel shift assay. Data quantification was performed using ImageJ.

### Western blotting

Tissues or cells were homogenized in lysis buffer (BioRad, Hercules, CA, USA) with proteinase and phosphorylase inhibitor cocktail (Thermo Fisher Scientific, Carlsbad, CA, USA), centrifuged for 15 min (12,000 rpm, 4 °C). The protein concentration was determined using the BCA Protein Assay Kit (SolarBio, Beijing, China). Twenty micrograms of total protein was subjected to sodium dodecyl sulfate-polyacrylamide gel electrophoresis and transferred onto PVDF membranes. The primary antibodies were diluted in tris-buffered saline with Tween solution buffer following the manufacturer's instructions and incubated with membranes overnight at 4 °C. The horseradish peroxidase-conjugated secondary antibodies were diluted at 1:1,000. Membranes were visualized by using the ECL Western blotting reagent (Tanon, Shanghai, China). The gel was normalized by ImageJ software (National Institutes of Health, USA).

### Animal assay

All animal experiments were undertaken in accordance with the National Institute of Health Guide for the Care and Use of Laboratory Animals, and all procedures were approved by the Committee of Experimental Animals of the Ocean University of China. C57BL/6 mice were purchased from Beijing Vital River Laboratory Animal Technology Co (Beijing, China). To generate xenograft models, B16F10 cells (1 × 10^5^ cells/injection) were inoculated via subcutaneous injection into 6-week-old C57BL/6 male mice. On day 11 after injection, mice were given an intratumoral injection of PBS (control), FAM-siRNA, and FAM-siRNA-loaded NH_2_-SSNs, respectively. After 6 h, the mice were euthanized. Tumors were dissected, frozen sliced, and observed under a ZEISS Vert.A1 fluorescence microscope (Carl Zeiss, Heidenheim, Germany).

For STING knockdown in vivo, the 6-week-old C57BL/6 male mice were randomly divided into 2 groups and intravenously injected with 200 μl of PBS (control), and STING siRNA (4 μg) loaded NH_2_-SSNs, respectively. After 24 h, mice were euthanized and the blood was collected. Subsequently, the red blood cell was removed by Red Blood Cell Lysis Buffer (R1010, SolarBio, Beijing, China) for 15 min. After centrifugation at 2,000 rpm, the white blood cell was collected. The heart, liver, lung, kidney, and brain were collected after perfusing by PBS. Total RNA was isolated using TRIzol reagent and the STING RNA level was evaluated by qRT-PCR.

For biodistribution of NH_2_-SSN-2, Cy5-siRNA (100 pmol) loaded NH_2_-SSN-2 (200 μg) in 200 μl of PBS was administrated intravenously via the tail vein. After 1 h, the mice were sacrificed. The major organs including heart, liver, spleen, lung, and kidney were collected and visualized by using the Lumina IVIS III Imaging System (PerkinElmer, Waltham, MA, USA) at excitation = 620 nm and emission = 670 nm.

For cytotoxicity and biocompatibility of NH_2_-SSN-2 in vivo, the 6-week-old C57BL/6 male mice were intravenously injected with 200 μl of PBS (control) and NH_2_-SSN-2 (final concentration is 100, 200, and 400 mg/kg). The body weight was measured every day. After 14 d, the mice were euthanized and the major organs were collected in 4% Paraformaldehyde. The H&E staining was supported by Serivebio (Wuhan, China).

### Modeling the SSN structure

To prepare an atomistic miniature model of a SSN (diameter of 4 to 5 nm), the structures of the initiator and terminator end groups and the monomer unit were sketched using the structure of orthosilicic acid. The polymer chains of 1 to 5 nm were grown using the Polymer Builder tool of Schrödinger’s Materials Science Suite (Schrödinger, LLC, New York, NY, 2021). The repeating units of this polymer structure were created along the X, Y, and Z axis using the 3D Builder Panel. Using the Nanoparticle Builder Panel, a 1- to 2-nm spherical nanoparticle was created. The silica atoms of the orthosilicic acid molecules were bridged with each other via the oxygen atoms of the hydroxyl groups (Si–O–Si). The terminal hydroxyl groups (Si–OH, silanol groups) were left on the surface of the nanoparticles. At specified distances (1.1 to 1.2 nm), 8 spike-like projections of orthosilicic acid were created by sketching. To create the amino-functionalized SSN-2, 2-aminoethyl silane groups were grown over the surface of the nonfunctionalized SSN-2 by covalent linking to silanol groups on the outer surface. The clashes between Si–O–Si and Si–OH atoms were removed by energy minimization of the structure using the 3D Builder Panel at each step.

### MD simulations

To prepare a simulation system of an SSN-2 with our different types of lipid bilayers, i.e., POPE, POPC, DMPC, DPPC, the System Builder panel of the Desmond module was used (Schrödinger Release 2021-4: Desmond Molecular Dynamics System, D. E. Shaw Research, New York, NY, USA, 2021. Maestro-Desmond Interoperability Tools, Schrödinger, New York, NY, USA, 2021) [8]. The position of the membrane was adjusted, keeping a distance of 20 Å from the apex of the SSN-2 to the C2 atoms of the membrane. For each system, an orthorhombic unit cell of 20x20x20 Å^3^ box size and periodic boundary conditions were used.

Each SSN-2-membrane containing system was submitted to a 100-ns MD simulation. The simulations were performed in the OPLS4e force field using the Molecular Dynamics Panel of Desmond [9]. The simulation systems were relaxed using a 6-stage membrane relaxation protocol. Briefly, the relaxation protocol involved 100 ps of Brownian dynamics (BD) at 10 K to remove steric clashes, followed by a short 20-ps BD simulation at 100 K and 1000 bar pressure in the NPT ensemble with a water barrier and the membrane restrained in the Z direction. In the third stage, a 100-ns MD simulation at 100 K was carried out using a water barrier and the NPγT ensemble with restrains on the membrane. In the fourth stage, the system was heated from 100 to 300 K in the NPγT ensemble with a gradual release of restraints for 150 ps. In the fifth stage, a 50-ns MD simulation in the NVT ensemble was performed with restrained heavy atoms at 300 K, followed by another 50-ps simulation in the final stage without any restraints. The production simulations were then performed for 100 ns at 300 K and 1.01325 bar with the Nosé-Hoover chain thermostat [10–12] and barostat using the Martyna-Tobias-Klein method with isotropic coupling in the NPγT ensemble [13]. The Coulombic method used for long-range interactions was U-series while the cut-off radius for short-range interactions was set to 9.0 Å [14]. Lipid density analysis was carried out using the trajectory density analysis tool, and the orientation and alignment of lipid molecules in the bilayer were determined by calculating the tilt angle with the surfactant tilt angle calculation tool of Schrödinger’s Materials Science Suite (release 2021-4). The total energy and distance calculations were carried out by using Schrödinger’s Materials Science workspace tools. The results were further analyzed using Microsoft Office 365 tools.

### Statistical analysis

All experiments were performed in triplicate, and results were presented as means ± SD, and the *P* value was calculated using GraphPad Prism 8.0 software (GraphPad Software Inc., San Diego, CA, USA) by the Student unpaired 2-tailed *t* test and 1-way ANOVA analysis. A *P* value less than 0.05 was considered statistically significant.

## Data Availability

Data supporting the findings of this study are available in the main text or the supplementary information.

## References

[1] Robbins PD, Ghivizzani SC. Viral vectors for gene therapy. Pharmacol Ther. 1998;80:35–47. 9804053

[2] Angel J, Franco MA, Greenberg HB. Rotaviruses. In: Mahy BWJ, Van Regenmortel MHV, editors. *Encyclopedia of virology.* 3rd ed. Oxford: Academic Press; 2008. pp 507–513.

[3] Kay MA, Glorioso JC, Naldini L. Viral vectors for gene therapy: The art of turning infectious agents into vehicles of therapeutics. Nat Med. 2001;7(1):33–40. 11135613 10.1038/83324

[4] Schiedner G, Morral N, Parks RJ, Wu Y, Koopmans SC, Langston C, Graham FL, Beaudet AL, Kochanek S. Genomic DNA transfer with a high-capacity adenovirus vector results in improved in vivo gene expression and decreased toxicity. Nat Genet. 1998;18(2):180–183. 9462752 10.1038/ng0298-180

[5] Shen C, Bradford SA, Imperiale MJ. Why are viruses spiked? mSphere. 2021;6(1):e01339-20. 33597174 10.1128/mSphere.01339-20 PMC8544902

[6] Yang J, Zhang X, Liu C, Wang Z, Deng L, Feng C, Tao W, Xu X, Cui W. Biologically modified nanoparticles as theranostic bionanomaterials. Prog Mater Sci. 2021;118:100768.

[7] Frouco G, Freitas FB, Coelho J, Leitão A, Martins C, Ferreira F. DNA-binding properties of african swine fever virus pA104R, a histone-like protein involved in viral replication and transcription. J Virol. 2017;91(12):e02498-16. 28381576 10.1128/JVI.02498-16 PMC5446646

[8] Cubuk J, Alston JJ, Incicco JJ, Singh S, Stuchell-Brereton MD, Ward MD, Zimmerman MI, Vithani N, Griffith D, Wagoner JA, et al. The SARS-CoV-2 nucleocapsid protein is dynamic, disordered, and phase separates with RNA. Nat Commun. 2021;12(1):1936. 33782395 10.1038/s41467-021-21953-3 PMC8007728

[9] Nayak S, Herzog RW. Progress and prospects: Immune responses to viral vectors. Gene Ther. 2010;17(3):295–304. 19907498 10.1038/gt.2009.148 PMC3044498

[10] Whitehead KA, Langer R, Anderson DG. Knocking down barriers: Advances in siRNA delivery. Nat Rev Drug Discov. 2009;8(2):129–138. 19180106 10.1038/nrd2742 PMC7097568

[11] Tomari Y. Perspective: Machines for RNAi. Genes Dev. 2005;19(5):517–529. 15741316 10.1101/gad.1284105

[12] Tang Z, Kong N, Zhang X, Liu Y, Hu P, Mou S, Liljeström P, Shi J, Tan W, Kim JS, et al. A materials-science perspective on tackling COVID-19. Nat Rev Mater. 2020;5(11):847–860. 33078077 10.1038/s41578-020-00247-y PMC7556605

[13] Tang Z, Zhang X, Shu Y, Guo M, Zhang H, Tao W. Insights from nanotechnology in COVID-19 treatment. Nano Today. 2021;36:101019. 33178330 10.1016/j.nantod.2020.101019 PMC7640897

[14] Akinc A, Maier MA, Manoharan M, Fitzgerald K, Jayaraman M, Barros S, Ansell S, Du X, Hope MJ, Madden TD, et al. The onpattro story and the clinical translation of nanomedicines containing nucleic acid-based drugs. Nat Nanotechnol. 2019;14(12):1084–1087. 31802031 10.1038/s41565-019-0591-y

[15] Mulligan MJ, Lyke KE, Kitchin N, Absalon J, Gurtman A, Lockhart S, Neuzil K, Raabe V, Bailey R, Swanson KA, et al. Phase I/II study of COVID-19 RNA vaccine BNT162b1 in adults. Nature. 2020;586(7830):589–593. 32785213 10.1038/s41586-020-2639-4

[16] Zhu Y, Xu P, Zhang X, Wu D. Emerging porous organic polymers for biomedical applications. Chem Soc Rev. 2022;51(4):1377–1414. 35043817 10.1039/d1cs00871d

[17] Croissant JG, Fatieiev Y, Khashab NM. Degradability and clearance of silicon, organosilica, silsesquioxane, silica mixed oxide, and mesoporous silica nanoparticles. Adv Mater. 2017;29(9):1604634. 10.1002/adma.201604634 28084658

[18] Bukara K, Schueller L, Rosier J, Martens MA, Daems T, Verheyden L, Eelen S, Van Speybroeck M, Libanati C, Martens JA, et al. Ordered mesoporous silica to enhance the bioavailability of poorly water-soluble drugs: Proof of concept in man. Eur J Pharm Biopharm. 2016;108: 220–225. 27648957 10.1016/j.ejpb.2016.08.020

[19] Song H, Yu M, Lu Y, Gu Z, Yang Y, Zhang M, Fu J, Yu C. Plasmid DNA delivery: Nanotopography matters. J Am Chem Soc. 2017;139(50):18247–18254. 29151352 10.1021/jacs.7b08974

[20] Wang W, Wang P, Tang X, Elzatahry AA, Wang S, Al-Dahyan D, Zhao M, Yao C, Hung CT, Zhu X, et al. Facile synthesis of uniform virus-like mesoporous silica nanoparticles for enhanced cellular internalization. ACS Cent Sci. 2017;3(8):839–846. 28852697 10.1021/acscentsci.7b00257 PMC5571464

[21] Niu Y, Yu M, Hartono SB, Yang J, Xu H, Zhang H, Zhang J, Zou J, Dexter A, et al. Nanoparticles mimicking viral surface topography for enhanced cellular delivery. Adv Mater. 2013;25(43):6233–6237. 23946251 10.1002/adma.201302737

[22] Lee C, Hwang HS, Lee S, Kim B, Kim JO, Oh KT, Lee ES, Choi H-G, Youn YS. Rabies virus-inspired silica-coated gold nanorods as a photothermal therapeutic platform for treating brain tumors. Adv Mater. 2017;29(13):1605563. 10.1002/adma.201605563 28134459

[23] Häffner SM, Parra-Ortiz E, Browning KL, Jørgensen E, Skoda MWA, Montis C, Li X, Berti D, Zhao D, Malmsten M. Membrane interactions of virus-like mesoporous silica nanoparticles. ACS Nano. 2021;15(4):6787–6800. 33724786 10.1021/acsnano.0c10378

[24] Chen HJ, Hang T, Yang C, Liu D, Su C, Xiao S, Liu C, Lin D-a, Zhang T, Jin Q, et al. Functionalized spiky particles for intracellular biomolecular delivery. ACS Cent Sci. 2019;5(6):960–969. 31263755 10.1021/acscentsci.8b00749 PMC6598163

[25] Kuo CW, Lai JJ, Wei KH, Chen P. Studies of surface-modified gold nanowires inside living cells. Adv Funct Mater. 2007;17(18):3707–3714.

[26] Gratton SEA, Ropp PA, Pohlhaus PD, Luft JC, Madden VJ, Napier ME, DeSimone JM. The effect of particle design on cellular internalization pathways. Proc Natl Acad Sci USA. 2008;105(33):11613–11618. 18697944 10.1073/pnas.0801763105 PMC2575324

[27] Wang Y, Tang J, Yang Y, Song H, Fu J, Gu Z, Yu C. Functional nanoparticles with a reducible tetrasulfide motif to upregulate mRNA translation and enhance transfection in hard-to-transfect cells. Angew Chem Int Ed. 2020;59(7):2695–2699. 10.1002/anie.201914264 31820548

[28] Wang Y, Du X, Liu Z, Shi S, Lv H. Dendritic fibrous nano-particles (DFNPs): Rising stars of mesoporous materials. J Mater Chem A. 2019;7(10):5111–5152.

[29] Stöber W, Fink A, Bohn E. Controlled growth of monodisperse silica spheres in the micron size range. J Colloid Interface Sci. 1968;26(1):62–69.

[30] Carcouët CC, van de Put MW, Mezari B, Magusin PC, Laven J, Bomans PH, Friedrich H, Esteves AC, Sommerdijk NA, van Benthem RA, et al. Nucleation and growth of monodisperse silica nanoparticles. Nano Lett. 2014;14(3):1433–1438. 24499132 10.1021/nl404550d

[31] Li S, Wan Q, Qin Z, Fu Y, Gu Y. Understanding Stӧber silica's pore characteristics measured by gas adsorption. Langmuir. 2015;31(2);824–832. 10.1021/la5042103 25514625

[32] Patwardhan SV, Emami FS, Berry RJ, Jones SE, Naik RR, Deschaume O, Heinz H, Perry CC. Chemistry of aqueous silica nanoparticle surfaces and the mechanism of selective peptide adsorption. J Am Chem Soc. 2012;134(14):6244–6256. 22435500 10.1021/ja211307u

[33] Mousavi M, Fini E. Silanization mechanism of silica nanoparticles in bitumen using 3-aminopropyl triethoxysilane (APTES) and 3-glycidyloxypropyl trimethoxysilane (GPTMS). ACS Sustain Chem Eng. 2020;8(8):3231–3240.

[34] Babaei M, Eshghi H, Abnous K, Rahimizadeh M, Ramezani M. Promising gene delivery system based on polyethylenimine-modified silica nanoparticles. Cancer Gene Ther. 2017;24(4):156–164. 28128214 10.1038/cgt.2016.73

[35] Nguyen TN, Angkawidjaja C, Kanaya E, Koga Y, Takano K, Kanaya S. Activity, stability, and structure of metagenome-derived LC11-RNase H1, a homolog of Sulfolobus tokodaii RNase H1. Protein Sci. 2012;21(4):553–561. 22389131 10.1002/pro.2043 PMC3375755

[36] Agarwal A, Mallapragada SK. Synthetic sustained gene delivery systems. Curr Top Med Chem. 2008;8(4):311–330. 18393894

[37] Brunner S, Sauer T, Carotta S, Cotten M, Saltik M, Wagner E. Cell cycle dependence of gene transfer by lipoplex, polyplex and recombinant adenovirus. Gene Ther. 2000;7(5):401–407. 10694822 10.1038/sj.gt.3301102

[38] Lim SH, Liao IC, Leong KW. Nonviral gene delivery from nonwoven fibrous scaffolds fabricated by interfacial complexation of polyelectrolytes. Mol Ther. 2006;13(6):1163–1172. 16497560 10.1016/j.ymthe.2005.12.016 PMC2409000

[39] Bhatia P, Taylor WR, Greenberg AH, Wright JA. Comparison of glyceraldehyde-3-phosphate dehydrogenase and 28S-ribosomal RNA gene expression as RNA loading controls for northern blot analysis of cell lines of varying malignant potential. Anal Biochem. 1994;216(1):223–226. 8135355 10.1006/abio.1994.1028

[40] Behzadi S, Serpooshan V, Tao W, Hamaly MA, Alkawareek MY, Dreaden EC, Brown D, Alkilany AM, Farokhzad OC, Mahmoudi M. Cellular uptake of nanoparticles: Journey inside the cell. Chem Soc Rev. 2017;46(14):4218–4244. 28585944 10.1039/c6cs00636a PMC5593313

[41] Kapara A, Brunton V, Graham D, Faulds K. Investigation of cellular uptake mechanism of functionalised gold nanoparticles into breast cancer using SERS. Chem Sci. 2020;11(22):5819–5829. 34094083 10.1039/d0sc01255f PMC8159335

[42] Sun H, Wong EHH, Yan Y, Cui J, Dai Q, Guo J, Qiao GG, Caruso F. The role of capsule stiffness on cellular processing. Chem Sci. 2015;6(6):3505–3514. 28706710 10.1039/c5sc00416k PMC5492901

[43] Teng Z, Wang C, Tang Y, Li W, Bao L, Zhang X, Su X, Zhang F, Zhang J, Wang S, et al. Deformable hollow periodic mesoporous organosilica nanocapsules for significantly improved cellular uptake. J Am Chem Soc. 2018;140(4):1385–1393. 29281272 10.1021/jacs.7b10694

[44] Rennick JJ, Johnston APR, Parton RG. Key principles and methods for studying the endocytosis of biological and nanoparticle therapeutics. Nat Nanotechnol. 2021;16(3):266–276. 33712737 10.1038/s41565-021-00858-8

[45] Hernández NE, Hansen WA, Zhu D, Shea ME, Khalid M, Manichev V, Putnins M, Chen M, Dodge AG, Yang L, et al. Stimulus-responsive self-assembly of protein-based fractals by computational design. Nat Chem. 2019;11(7):605–614. 31209296 10.1038/s41557-019-0277-y

[46] Haag SM, Gulen MF, Reymond L, Gibelin A, Abrami L, Decout A, Heymann M, van der Goot FG, Turcatti G, Behrendt R, et al. Targeting STING with covalent small-molecule inhibitors. Nature. 2018;559(7713):269–273. 29973723 10.1038/s41586-018-0287-8

