# Supplementary material for: Silica Nanoparticles with Virus-Mimetic Spikes Enable Efficient siRNA Delivery In Vitro and In Vivo
Source: Research (Wash D C). 2022 Dec 21;2022:0014. doi: 10.34133/research.0014 (PMC11407519; doi:10.34133/research.0014)
Supplement: Supplementary Materials — Figs. S1 to S12. Tables S1 and S2. [file research.0014.f1.docx]

Title

Silica Nanoparticles with Virus-Mimetic Spikes Enable Efficient siRNA Delivery in vitro and in vivo

**Authors**

Jianye Fu^1,2,3,4,†^, Wenwei Han^1,3,†^, Xue Zhang^1,†^, Yutong Sun^1,†^, Rajendra Bhadane^5,6^, Bo Wei^1^, Li Li^1,7,8^, Liangmin Yu^3^, Jinbo Yang^1,7,8^, Jessica M. Rosenholm^5^, Outi M. H. Salo-Ahen^5,6^, Taojian Fan^2^, Bin Zhang^2^, Wageh Swelm^9^, Ahmed A. Al-Ghamdi^9^, Lin Xia^10^, Han Zhang^2,*^, Meng Qiu^3,*^, Hongbo Zhang^6,11,*^, Xin Wang^1,7,8,*^

**Affiliations**

^1^ Key Laboratory of Marine Drugs, Chinese Ministry of Education, School of Medicine and Pharmacy, Ocean University of China, Qingdao 266003, China

^2^ Collaborative Innovation Center for Optoelectronic Science & Technology, International Collaborative Laboratory of 2D Materials for Optoelectronics Science and Technology of Ministry of Education, Institute of Microscale Optoelectronics, College of Physics and Optoelectronic Engineering, Shenzhen University, Shenzhen 518060, P. R. China

^3^ Key Laboratory of Marine Chemistry Theory and Technology (Ocean University of China), Ministry of Education, Qingdao 266100, China.

^4^ College of Chemistry and Chemical Engineering, China University of Petroleum, Qingdao 266555, China

^5^ Pharmaceutical Sciences Laboratory, Åbo Akademi University, 20520, Turku, Finland

^6^ Structural Bioinformatics Laboratory, Biochemistry, Åbo Akademi University, 20520, Turku, Finland

^7^ Center for Innovation Marine Drug Screening & Evaluation, Pilot National Laboratory for Marine Science and Technology (Qingdao), Qingdao, 266237, China.

^8^ Marine Biomedical Research Institute of Qingdao, Qingdao 266100, China.

^9^ Department of Physics, Faculty of Science, King Abdulaziz University, Jeddah 21589, Saudi Arabia.

^10^ Hangzhou No. 14 High School, Hangzhou, 310000, China.

^11^ Turku Bioscience Centre, University of Turku and Åbo Akademi University, 20520 Turku, Finland

† These authors contributed equally to this work

Corresponding Authors:

Meng Qiu, Email: mengqiu@ouc.edu.cn

Han Zhang, Email: hzhang@szu.edu.cn

Hongbo Zhang, Email: hongbo.zhang@abo.fi

Xin Wang, Email: wx8399@ouc.edu.cn

**Abstract**

Oligonucleotide-based therapy has experienced remarkable development in the past two decades, but its broad applications are severely hampered by delivery vectors. Widely used viral vectors and lipid nanovectors are suffering from immune clearance after repeating usage, or requiring refrigerated transportation and storage, respectively. In this work, amino-modified virus-mimetic spike silica nanoparticles (NH_2_-SSNs) were fabricated using a one-pot surfactant-free approach with controlled spike lengths, which were demonstrated with excellent delivery performance and biosafety in nearly all cell types and mice. It indicated that NH_2_-SSNs entered cells by spike-dependent cell membrane docking and dynamin-dependent endocytosis. The positively charged spikes with proper length on the surface can facilitate the efficient encapsulation of RNAs, protect the loaded RNAs from degradation, and trigger an early endosome escape during intracellular trafficking, similarly to the cellular internalization mechanism of virions. Regarding the fantastic properties of NH_2_-SSNs in nucleic acid delivery, it revealed that nanoparticles with solid spikes on the surface would be excellent vehicles for gene therapy, presenting self-evident advantages in storage, transportation, modification, and quality control in large-scale production compared to lipid nanovectors.

**Keywords**

silica nanoparticles, virus-mimetic spike, intracellular delivery, siRNA, gene therapy

1. **Introduction**

Oligonucleotide-based therapy has experienced remarkable development in the past two decades, but its universal applications are severely hampered by delivery vectors. Viruses are native nanovectors with prominent advantages in gene delivery.^[1, 2, 3, 4, 5, 6]^ The evolutionary success gives viruses a set of excellent strategies to deliver DNA/RNA into cells. For example, the African swine fever virus uses a positively charged histone-like protein to encompass DNA, while SARS-CoV-2 uses N protein to protect RNA via a liquid-liquid phase separation strategy.^[7, 8]^ Usually, Virions attach to the cell surface and activate endocytosis by hijacking cellular pathways. Interestingly, in a successful infection, viral protein may penetrate the endosomal membrane and escape from the intracellular traffic cargos. However, viral vectors will trigger immune clearance after repeated usage in the body.^[9]^ It obviously limits the utilization of viral vectors in gene therapies, especially in siRNA-based therapies.^[10, 11]^ Besides, the first FDA-approved RNA interference (RNAi) medicine (Onpattro®, patisiran) was indeed a lipid-based nano-formulation, while the Moderna and Pfizer/BioNTech COVID-19 vaccines that have enjoyed great success globally are also based on LNP formulations similar to that of Onpattro®.^[12, 13]^ The commercial Lipofectamine 3000 (abbreviated as Lipo3000) and RNAiMAX are typical LNP-based gene delivery tools in the lab.^[14]^ Nevertheless, LNPs are suffering from different types of obstacles that limit their broad application. The soft-state nature of LNPs is subject to chemical and physical instability for long-term use, which poses limitations both from a storage point of view as well as repeated administrations due to the immediate disintegration of the LNP upon entering the cell.^[15]^

To overcome these issues, a virus-mimetic inorganic nanovector will take the advantage of viral vectors and avoid the above disadvantages.^[16]^ In theory, the positively charged surface of nanovectors will efficiently encapsulate RNAs, protect loading RNAs against RNases, and attach the negatively charged cell membrane tightly. Fantastically, it is possible to design an open virion-like nanovector instead of the closed native virion for gene delivery because previous studies have shown that the tough surface spike of nanovectors will facilitate their internalization. The tough spikes on the surface of nanovectors may penetrate through the cell membrane and endosomal membrane, which initiates the internalization of nanovectors and provide a convenience for their endosomal escape. Also, the densely arranged spikes on the surface may provide a solid-liquid phase separation microenvironment for RNAs as a shelter during gene delivery. A good virus-mimetic inorganic nanovector with not only the spike structure but also the positively charged surface chemistry and open porous structure can greatly take the advantage of viral vector and avoid its disadvantages. Amorphous colloidal silica are appealing materials and are widely used in biomedical applications due to their good biocompatibility, reflected by their approval by the United States Food and Drug Administration (FDA) as GRAS (generally regarded as safe) materials.^[17]^ The specific class of mesoporous silica nanoparticles (MSNs) has for instance been successfully applied as a drug delivery vector in a clinical trial.^[18]^ Besides, researchers have found that synthetic nanoparticles with rambutan-like spike or virus-like surface nano-topography exhibit high cellular uptake and gene delivery efficacy.^[19, 20, 21, 22, 23]^

It is hypothesized that the spike length plays an important role. Due to the lack of robust synthesis techniques to fabricate comparable nanoparticles with various spike lengths, it has not been possible to address this question. The surface spikes are regarded as the first touch toward cell membranes, and the cellular internalization process facilitates the engulfment of spike particles. The spike mosaic proteins (fusion proteins, FPs) in the membrane of enveloped viruses (as well as the naked forms in non-envelope viruses) facilitate viral invasion by attaching virions to their receptors and triggering the endocytosis process. The engulfment process generates a force on the surface spikes and induces cell membrane penetration.^[24, 25, 26]^ In this work, we invented a one-pot synthetic method to produce silica-based nanoparticles with precisely tunable surface spike structures and spike lengths. Notably, the spikes are also silica-based, which is mechanically strong. Here, a series of virus-mimetic spiky silica nanoparticles (SSNs) with different spike lengths were successfully fabricated through a competitive epitaxial growth approach in a surfactant-free aqueous reaction system. The novel technique allows the formation of monodisperse SSNs with tunable surface spike length, as well as narrow size distribution, fine controllable spike length, and inner core diameter. It was demonstrated that the SSNs with optimized surface spike lengths showed extraordinary siRNA delivery efficacy in cells.^[27, 28]^ In vivo, the SSNs also exhibited low toxicity, suggesting the potential of SSNs as next-generation robust siRNA delivery vectors.

1. **Results**

**2.1. Synthesis of SSNs**

The SSNs with uniform morphology were synthesized in an ethanol-water reaction system without the participation of any surfactants. Ethylenediamine (EDA) was used as a cationic linker for the two negative polymerization systems (TEOS and APF polymer) and ammonia was employed as a catalyst. During the synthesis, TEOS first hydrolyzed and condensed to form silica primary particles, which further collided to form silica core particles (Figure 1a). Afterwards, AP and F began to form APF oligomer/polymer, which condensed competitively with silica primary particles on the silica core particles. The FP-like silica spikes were generated by the existence of the surrounding APF polymer matrix, and the silica spike length was controlled by the delayed addition of AP and F. The initial formation process was very similar to the classic Stöber method,^[29, 30]^ and the following competitive condensation process of silica primary particles and APF oligomers was used to control the growth of the surface spikes. The competitive condensation process allowed the epitaxial growth of silica spikes and the formation of SSNs after calcination of the APF polymer in air. During the synthesis, it was observed that the core size gradually increased and the spike length gradually decreased under various synthetic temperatures, 60 °C (Figure S1a-i) or 30 °C (Figure S1j-r), respectively. Statistical results of the surface spikes’ length on SSNs are shown in Figure S1s. It is seen that our synthetic strategy can be used to finely control the spike length and is capable of preparing SSNs with various desired spike lengths. Moreover, by further analyzing the reaction kinetics and spike formation process, the relationship between spike length and the delayed addition time interval was proposed as shown in Figure S1t, which could instruct the fabrication of SSNs with desired spike lengths. Among all the prepared SSNs, four types of representative SSNs with comparable nanoparticles size but various spikes lengths were selected, and detailed characterizations were performed to demonstrate the influences of spike length as siRNA delivery vectors.

**2.2. Characterizations of SSNs**

Transmission electron microscopy (TEM) images of the obtained four SSNs (SSN-1, SSN-2, SSN-3, and SSN-4) clearly showed that the particles have good dispersity, uniform and comparable nanoparticle sizes (Figure 1b-f). It could be seen that these SSNs were composed of two parts: (i) solid silica cores with diameter increased gradually from ~50 nm (SSN-1, Figure 1b) to ~200 nm (SSN-4, Figure 1e); (ii) gradually decreased spike length (Figure 1g) from ~80 nm (SSN-1) to ~10 nm (SSN-4). The size of the silica cores and the length of the surface spikes could be tuned by simply delaying the addition of APF polymer precursors (AP and F) (see the experimental section for more details), which would reduce the reaction time of the competitive condensation process. The delayed addition of AP and F allowed a continuous consumption of TEOS in the formation of silica cores, while less TEOS (or silica primary particles) remained for the following condensation to generate silica spikes.

In order to characterize the porous nature and texture properties of these SSNs, a nitrogen sorption analysis was performed. As shown in Figure 1h, SSN-1, SSN-2, and SSN-3 exhibited typical type IV isotherms, indicating the abundant mesopores in these nanoparticles. The hysteresis loop for SSN-4 could be barely observed, which suggested the limited mesopores in SSN-4. The prepared SSNs exhibited high specific surface areas of ~222-346 m^2^/g (SSN-1 to SSN-3, Table S1), except SSN-4 (53 m^2^/g), where the reduced spike length provided only limited porosity, similar to the surface area of solid nanospheres.^[31]^ The pore size distribution of SSNs calculated with the Barrett-Joyner-Halenda (BJH) method from the adsorption branch showed that it gradually increased from 6.8 nm (SSN-1) to 12.7 nm (SSN-3), then decreased to 5.8 nm (SSN-4) (Figure S2a, Table S1). Afterwards, SSNs were modified with amino functional groups for future applications. As shown in Figure 1i, all SSNs exhibited negative zeta potential values at neutral pH, which changed to positive after amino modification, consistent with literature reports.^[32, 33]^ Moreover, as shown in the FTIR spectrum (Figure 1j and Figure S2b), the bands observed at around 2929 cm^-1^ and 2883 cm^-1^ further supported the successful amino group grafting on SSNs.


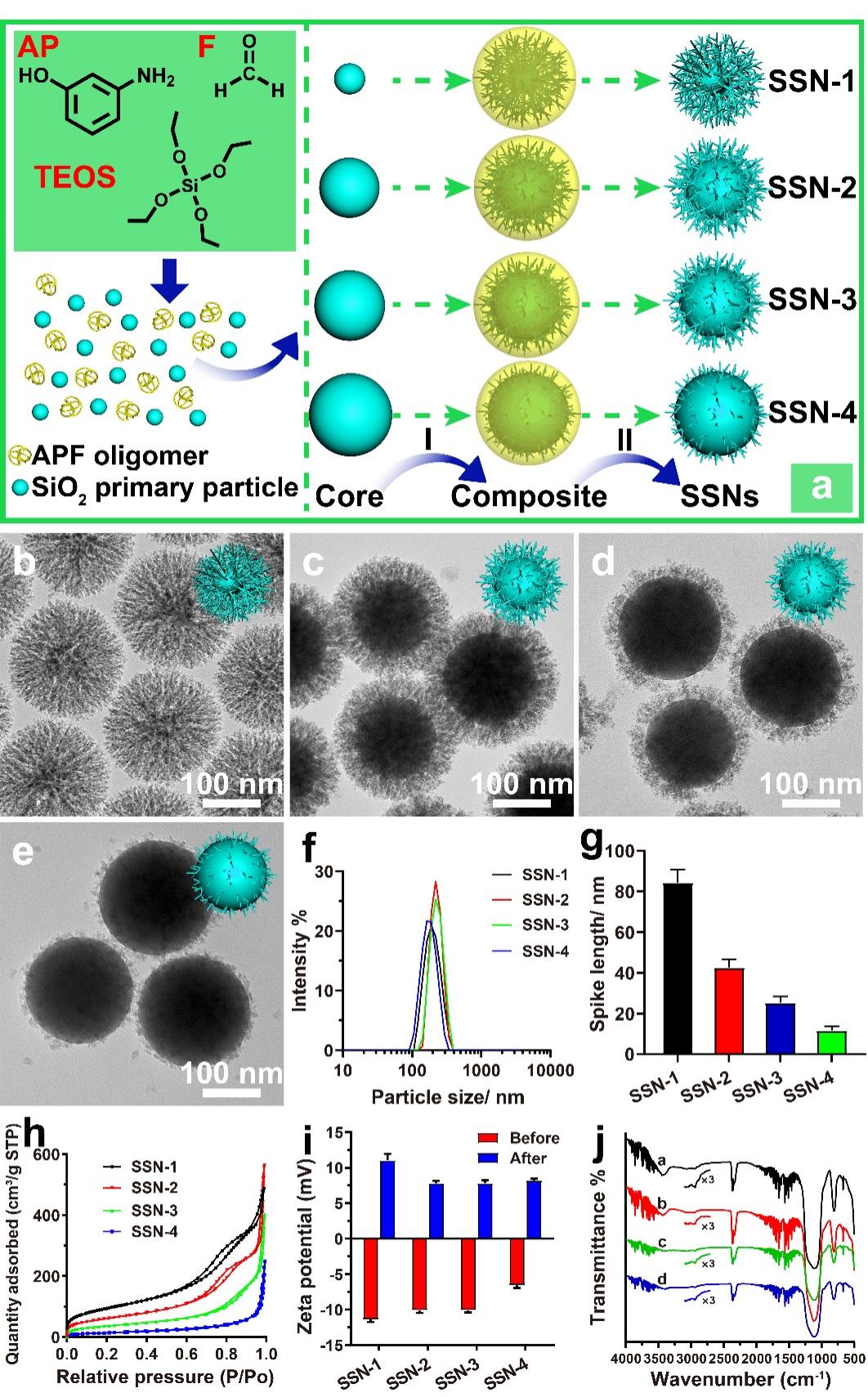


***Figure 1. Fabrication process and structural characterizations of SSNs.*** *Competitive self-assemble synthesis strategy and formation mechanism of SSNs (a). Self-assembly approaches employing controlled polymerization of TEOS and AP/F generate controlled cores. After continuous condensation (I), and calcination of the composites in air (II). SSNs with controlled spike length can be obtained by adjusting the process of competitive condensation kinetics. Representative TEM images of SSN-1 (b), SSN-2 (c), SSN-3 (d) and SSN-4 (e). f, Hydrodynamic size distribution as measured by dynamic light scattering (DLS). g, Statistical results of the length of surface spikes. h, Nitrogen adsorption-desorption isotherms. i, Zeta potential at neutral pH before and after amino modifications. j, FTIR spectrum of amino-modified SSNs. SSN-1 (a, black), SSN-2 (b, red), SSN-3 (c, green), and SSN-4 (d, blue).*

**2.3. siRNA delivery performance between various silica nanoparticles**

The ultimate goal of a robust siRNA gene vector is to deliver siRNA intracellularly in various types of cells, and release siRNA to initiate RNA interference.^[34]^ To highlight the critical influence of the surface spikes in siRNA delivery, conventional DMSNs with similar size were synthesized according to the literature and the characterizations of which were shown in Figure S2c-f. We first screened RNA delivery abilities and cellular cytotoxicity of various amino-modified SSNs (NH_2_-SSNs) and NH_2_-DMSN (Data not shown). It was found that NH_2_-SSN-2 had the best performance with the used amount of 200 μg/mL (Figure S3a). Cells maintained high viability even with a high amount of NH_2_-SSN-2 (1 mg/mL) in primary mouse embryonic fibroblast (MEF) cells (Figure S3b) and HEK-293T cells (Figure S3c), indicating its low cytotoxicity. As shown in Figure 2a&b, NH_2_-SSN-2/FAM-siRNA treated cells exhibited the strongest green fluorescence among all the formulations, indicating an efficient (~90.0% in HEK-293T) and successful delivery of siRNA inside the cells. Compared with Lipo3000/FAM-siRNA (~64.8%) and NH_2_-DMSN based formulations (Figure S3d), the surface spikes enable silica nanoparticles with strongly improved delivery performance. Importantly, higher FAM-siRNA delivery efficacy was observed for NH_2_-SSN-2 in BMDM cells, MEF cells, and HEK-293T cells (Figure S3e-g) when compared with other commercial reagents (Lipofectamine RNAiMAX).


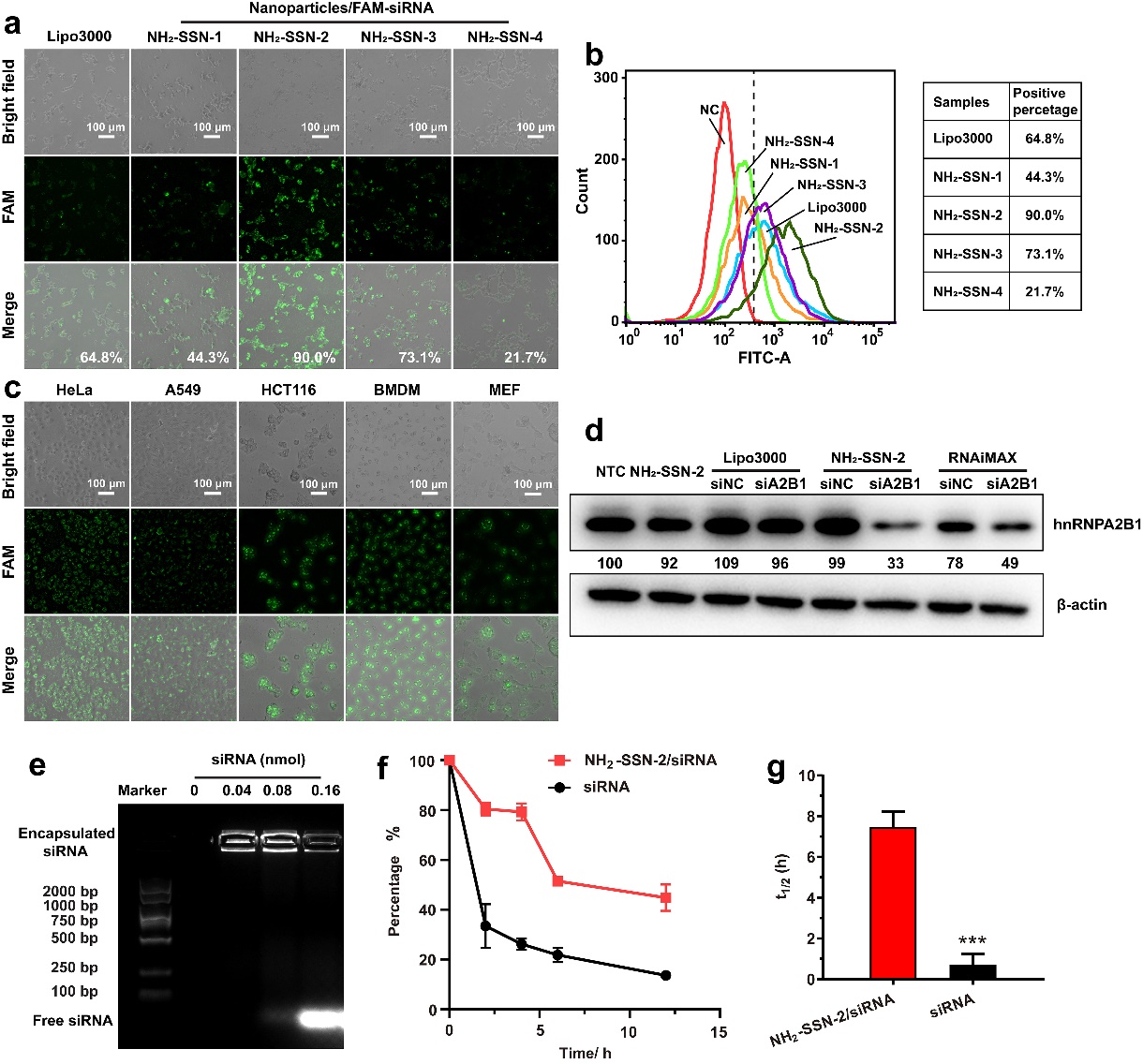


***Figure 2. Cellular internalization of siRNA-loaded nanoparticles.*** *SiRNA loading and protection performances. Green fluorescence-labeled siRNA (FAM-siRNA) was loaded on the amino-modified silica nanoparticles and directly added into the culture medium. a, Fluorescence images of RNA delivery by various silica nanoparticles loaded with FAM-siRNA in HEK-293T after incubation for 6 h. b, Flow cytometry evaluation of the delivery efficacy of NH_2_-SSNs in HEK-293T cells. c, Fluorescence images of RNA delivery by NH_2_-SSN-2 in various cell lines after incubation for 6 h. d, Gene knockdown by NH_2_-SSN-2 loaded siRNA. MEF cells were transfected with indicated formulations for 48 hours, and 20 μg of cellular lysates were assayed by Western-Blotting. Non-treatment control, NTC (lane 1), bare NH_2_-SSN-2 (lane 2), Lipo3000 loaded with scramble siRNA (siNC) (lane 3) and hnRNPA2B1 siRNA (lane 4), NH_2_-SSN-2 loaded with siNC (lane 5) and hnRNPA2B1 siRNA (lane 6), RNAiMAX loaded with siNC (lane 7) and hnRNPA2B1 siRNA (lane 8).* *The grey intensities of hnRNPA2B1 were estimated using Image J and normalized to β-actin. The relative gene expression was labeled. e, Gel-retardation assay of NH_2_-SSN-2 (100 µg) with various siRNA amounts. A constant amount of NH_2_-SSN-2 (100 µg) was mixed with various amounts (0-0.16 nmol) of siRNA, respectively. f, The protection ability of NH_2_-SSN-2 on siRNA in* *plasma.* *The siRNA or NH_2_-SSN-2 loaded siRNA was added to mouse plasma at room temperature, and the siRNA degradation results were* *determined by* *gel shift assay and analyzed by ImageJ. g, Calculated half-lives (t1/2) of the two formulations were analyzed through the siRNA degradation results and calculated by GraphPad Prism 8 software. All experiments were performed in triplicates. Results were analyzed by a two-tailed t-test and presented as mean ± SD, n = 3. ***, p < 0.001.*

**2.4. NH_2_**-**SSN-2** **targeted a broad range of cell types**

The length of surface spikes obviously influenced the delivery performance, since low FAM-siRNA delivery efficiency was observed for longer surface spikes (NH_2_-SSN-1, 44.3%) or shorter surface spikes (73.1% for NH_2_-SSN-3 & 21.7% for NH_2_-SSN-4) other than the moderate one (NH_2_-SSN-2) in HEK-293T cells. Fluorescence intensity analysis (Figure S4a-f) further confirmed the above observation where NH_2_-SSN-2 exhibited stronger fluorescence and higher delivery efficacy compared with other SSNs. To explore its universal nucleic acid delivery ability, NH_2_-SSN-2 was tested in all types of cells available in hand (Table S2), showing an excellent delivery capability. As shown in Figure 2c, NH_2_-SSN-2 exhibited an extraordinary delivery performance in cultured cell lines, such as HeLa, A549, as well as the hard-to-transfect HCT116. Gene delivery to primary cells is always a challenge for any reagent-based transfection or electroporation. Thus, it was interesting to notice that NH_2_-SSN-2 allowed highly efficient delivery in murine bone marrow-derived macrophages (BMDM, nearly 100%) and primary mouse embryonic fibroblast (MEF, more than 70%), which were otherwise particularly challenging for all nucleic acid delivery approaches in all labs (Figure 2c). It indicated that the SSN with appropriate spike length (NH_2_-SSN-2) could be employed as delivery vectors to target a broad range of cell types.

Western-Blotting assay was conducted to semi-quantitatively analyze the heterogeneous nuclear ribonucleoprotein A2/B1 (hnRNPA2B1) protein levels in the hard-to-transfected MEFs. As shown in Figure 2d and Figure S4g, it was not surprising to see a negligible reduction in lipofectamine transfected MEFs because of the poor transfection efficiency. Reduced hnRNPA2B1 protein level was detected in NH_2_-SSN-2 transfected groups when compared with the non-treatment control group (NTC), the bare nanoparticles group (NH_2_-SSN-2) and RNAiMAX treated group. It indicated that NH_2_-SSN-2 could deliver siRNA inside the cytosol and release siRNA to realize its function. Moreover, the expression of hnRNPA2B1 protein when scramble siRNA was used and results show that there is no intrinsic effect of the formulation on hnRNPA2B1 expression. Similar results were found in other cell lines (such as A549 cells, Figure S4h), revealing a broad target range of NH_2_-SSN-2.

**2.5. siRNA loading capability and protection performance of NH_2_-SSN-2**

To explore the delivery capability of NH_2_-SSN-2 in detail, its binding affinity toward siRNA was estimated by gel retardation assay.^19^ As shown in Figure 2e, a complete electrophoretic shift can be observed for free siRNA. No release of siRNA molecules was observed even at the high amount of siRNA (0.08 nmol), indicating that NH_2_-SSN-2 effectively encapsulated siRNA. The encapsulation rate is 1.3±0.1 μmol /g (siRNA per NH_2_-SSN-2). Furthermore, it demonstrated that the siRNA loading capability is highly related to the surface spike length, as shown in Figure S5, where siRNA loading capability decreased with the decrease of surface spike length. To evaluate whether the surface spikes of NH_2_-SSN-2 can protect the loaded siRNA, fresh murine plasma was used to mimic physiological environments. As shown in Figure 2f&g, it is interesting to observe that NH_2_-SSN-2 showed great protection of siRNA in plasma, and the elimination half-life (t_1/2_) of NH_2_-SSN-2 loaded siRNA (7.5 h) was significantly longer than naked siRNA (0.7 h). It is inferred that the pore size of NH_2_-SSN-2 (8.1 nm) is smaller than the size of RNase (~10 nm),^[35]^ and the siRNA molecules are hidden sterically behind the spikes, which eventually provide strong protection on the loaded siRNA molecules. It implied that NH_2_-SSN-2 could function as advanced siRNA delivery vectors with high loading capacity, robust delivery performance, and extraordinary protection performance.

**2.6. Repeated** **treatment of siRNA for long-term studies**

Compared to shRNA, siRNA transfection is convenient but limited for long-term studies, because transfected siRNA will be diluted into daughter cells with cell proliferation. It has been demonstrated that repeated treatment of gene vectors can ensure the therapeutic effect over several entire cell proliferation cycles, especially the mitotic stage where the highest delivery efficacy can be obtained. ^[36, 37, 38]^ However, most commercial reagents would influence cell growth and proliferation during repeated treatment, even though their cytotoxicity is almost negligible in a single usage. Therefore, the advanced delivery property of NH_2_-SSN-2 was shown in a repeated treatment assay for up to 8 days in MEF cells, which are very sensitive to cytotoxicity.

As a demonstration, hnRNPA2B1 in the nucleus, stimulator of interferon genes (STING) on the endoplasmic reticulum, and the house-keeping gene GAPDH in the cytoplasm were selected as the targeting gene (Figure 3a). Noticeably, GAPDH is an excellent candidate in Western-Blotting analyses as an internal loading control due to its stable expression in almost all tissues.^[39]^ Transfection complexes were added into the cell culture medium every 48 hours (Figure 3b). Significant cell viability reduction appeared in Lipo3000 incubated groups after 144 h treatment (Figure 3c), indicating the cytotoxicity of Lipo3000 during repeated treatment. As shown in Figure 3d, a reduction of GAPDH protein was detected in both groups 48 h post-transfection. With repeated treatment, it could be seen that a gradual knockdown of GAPDH protein was achieved. Only a limited amount of GAPDH protein can be detected after three repeated transfections. It demonstrated the feasibility of NH_2_-SSN-2 as a repeated treatment agent to realize sustained gene knockdown. Similar observations were found for hnRNPA2B1 and STING genes (Figure 3e&f). Moreover, the knockdown performance was statistically analyzed by measuring the grey values of the Western-Blotting results, which further supported the above observations (Figure S6).


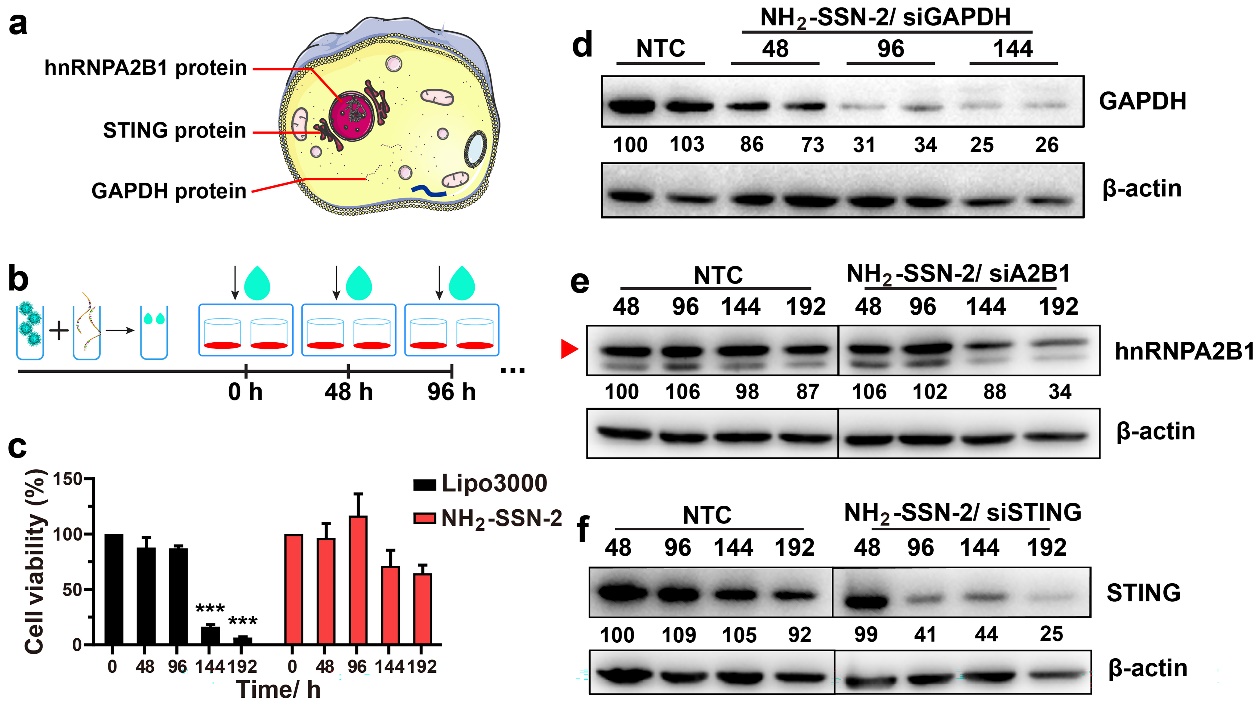


***Figure 3. Repeated treatment of siRNA.*** *a, Subcellular locations of three indicated genes. b, Schematic illustration of the repeated treatment. c, Cell viability of MEF cells after repeated treatment. Results were presented as mean ± SD and analyzed by one-way ANOVA. ***, p < 0.001, n = 3. d-f, Reduced protein levels in MEF cells after repeated treatment. 20 μg of cellular lysates were assayed by Western-Blotting using indicated antibodies. Experiments were repeated at least three times. The grey intensities of GAPDH, hnRNPA2B1, and STING were estimated using Image J and normalized to β-actin. The relative gene expression was labeled.*

**2.7. Cellular internalization of NH_2_-SSN-2**

To gain detailed cellular internalization information for NH_2_-SSN-2, a time-course analysis was performed to conduct the cellular uptake of nanoparticles. As shown in Figure 4a, cells started to internalize NH_2_-SSN-2/FAM-siRNA complexes as early as 2 hours post-incubation. In the meantime, the loaded FAM-siRNA started to release from NH_2_-SSN-2 as pointed out by arrows in the image. A large amount of FAM-siRNA could be successfully delivered inside the cytosol after ~4-6 h of incubation, demonstrating the efficient internalization performance of NH_2_-SSN-2 (other incubation time intervals in Figure S7). Nearly no green fluorescence was found in cells at 4 °C even after incubation for ~6 h, indicating that the cellular uptake was an energy-dependent process, e.g., endocytosis, rather than passive diffusion.^[40, 41, 42, 43]^

A consensus is currently developing for five major types of endocytosis: clathrin-coated pit-mediated endocytosis (CME, clathrin and dynamin-dependent), fast endophilin-mediated endocytosis (FEME, clathrin-independent but dynamin-dependent), clathrin-independent carrier (CLIC)/glycosylphosphatidylinositol-anchored protein enriched early endocytic compartment (GEEC) endocytosis (clathrin and dynamin independent), micropinocytosis, and phagocytosis.^44^ In the presence of various internalization inhibitors, representative fluorescence images (Figure S8a) and the quantitative measurement of intracellular amounts of FAM-siRNA (Figure S8b) demonstrated the cellular uptake of RITC labeled NH_2_-SSN-2, and the successful delivery of FAM-siRNA, with no significant toxic effects, were observed using the inhibitors under selected treated amounts (Figure S8c). The corresponding hnRNPA2B1 knockdown performance was shown in Figure 4b. It was noted that hnRNPA2B1 protein was significantly reduced under the treatment by siRNA-loaded NH_2_-SSN-2, whereas it was rescued by dynasore/EIPA but not by other internalization inhibitor treatments, indicating a dynamin-dependent pathway.^[44]^

To study how the amino-modified SSN-2 interacts with lipid membranes, we carried out MD simulations of four NH_2_-SSN-2-membrane systems using predefined single-lipid-component membranes, i.e., POPE, DMPC, POPC, and DPPC (Figure 4c-e). It was shown that even with POPE lipid bilayer there was no apparent fusion of the SSN-2 with membranes, suggesting that an active transport mechanism would be needed to facilitate the entry of the nanoparticle (more details could be found in Figure S9). Together with the unsuccessful internalization at 4 °C (Figure 5a) and the successful inhibition by internalization inhibitors, it clearly indicated that NH_2_-SSN-2 entered cells via energy- and dynamin-dependent manner.


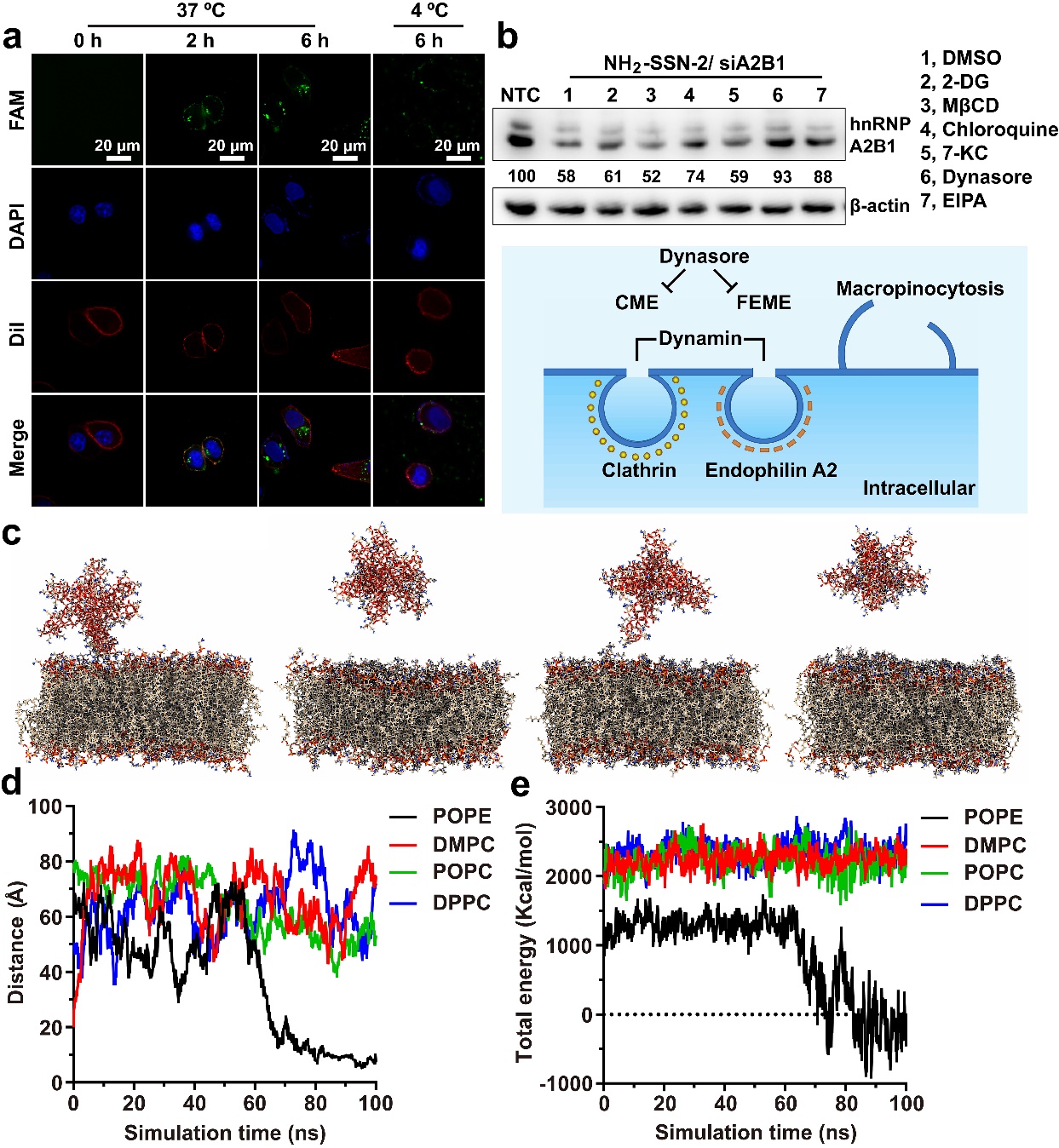


***Figure 4. Cellular internalization behavior.*** *a, Time-dependent fluorescence images of FAM-siRNA delivery by NH_2_-SSN-2 in MEF cells after incubation at 37 °C/4 °C for 0 to 6 h. The cell membrane was stained with DiI (red). b, In vitro determination of relative hnRNPA2B1 and β-actin protein levels in cells transfected with NH_2_-SSN-2 in the presence of various internalization inhibitors. 20 μg of cellular lysates were assayed by Western-Blotting. The grey intensities of hnRNPA2B were estimated using Image J and normalized to β-actin. The relative gene expression was labeled. Experiments were repeated at least three times. 2-DG (pan-inhibitor of energy-dependent endocytosis), MβCD (inhibitor of lipid rafts/cholesterol-enriched microdomains/caveolae pathway), Chloroquine (CME inhibitor), 7-KC (CLIC/GEEC inhibitor), Dynasore (CME & FEME inhibitor), EIPA (macropinocytosis inhibitor). c, Interaction of spike silica nanoparticles (SSN-2) with different lipid bilayers; (from left) SSN-2-POPE, SSN-2-DMPC, SSN-2-POPC, SSN-2-DPPC after 100-ns MD simulations (SSN-2 and lipids are shown in elemental ball-and-stick representation, Na^+^ and Cl^-^ ions, and water molecules are hidden for clarity). d, The distance between the silica atom (Si973) on the tip of the SSN-2 spike to the closest lipid C2 atom in the upper leaflet of the lipid bilayer during the 100-ns MD simulation. e, The total interaction energy was calculated as the sum of electrostatic and van der Waals interactions between the SSN-2 and the studied lipid membranes during the 100-ns MD simulation.*

**2.8. Cytosol release of NH_2_-SSN-2 loaded siRNA**

To further explain the high nucleic acid delivery performance of NH_2_-SSN-2 in cells, the subcellular localization of FITC-labeled nanoparticles and FAM-siRNA were examined in MEF cells (Figure 5a&b). After 6-hour incubation, some of the FAM-siRNA/nanoparticles formed a cycle around the cell surface, suggesting these formulations could first adhere to the cell surface, while some of the delivery vectors were observed internalized into the cytosol for SSNs-based delivery vectors. However, no green signals were found in NH_2_-DMSN treated cells, implying the failure of the cellular uptake of DMSN (Figure 5a lane 4). Merged peaks in the curve and colocalized green and red dots were found in NH_2_-SSN-1 treated cells, suggesting that FAM-siRNA failed to dissociate from NH_2_-SSN-1 (Figure 5a lane 1). Surprisingly, abundant green dots and diffused red fluorescence were found in NH_2_-SSN-2 treated cells, indicating a successful siRNA release from nanocarriers (Figure 5a lane 2). Although siRNAs could easily be released from NH_2_-SSN-4 (Figure 5a lane 3), the nucleic acid delivery efficiency was poor because it could not load enough siRNAs (Figure S5). It suggested that the surface spike length was related to siRNA loading abilities and siRNA release. It demonstrated that the high nucleic acid delivery performance of NH_2_-SSN-2 was related to its high cellular internalization capability and siRNA release. The separated subcellular localization of NH_2_-SSN-2 and late endosomes/lysosomes marker (CD63) indicated successful endosomal escape (Figure 5c), which avoided the rapid degradation of RNAs in cells. The release of siRNA from NH_2_-SSN-2 is further semi-quantitatively analyzed by measuring the percentage of co-localization/ separated red and green signals in over 20 cells (Figure S10). It is shown that FAM-siRNA starts to release from the late endosomes/lysosomes after 2 h of incubation, and has almost totally released after 6 h of incubation.


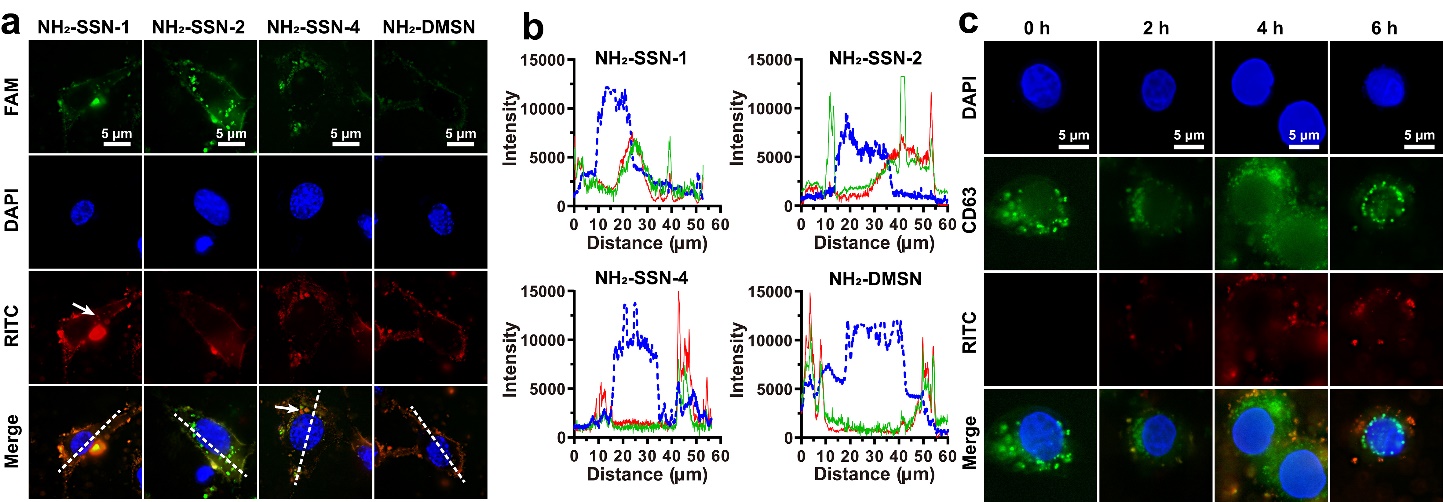


***Figure 5. Endosomal escape.*** *The nanoparticles and siRNA were labeled with RITC (red) and FAM (green), respectively. The nucleus was stained with DAPI (blue). a, Fluorescence images of FAM-siRNA delivery by NH_2_-SSN-1 (long surface spikes),* *NH_2_-SSN-2 (moderate surface spikes), NH_2_-SSN-4 (short surface spikes), and no surface spike NH_2_-DMSN in MEF cells. b, Distribution of the three fluorescence along the dashed lines in the merged images from a. Nanoparticle (red), RNA (green), and nucleus (blue). c, Cellular internalization of* *NH_2_-SSN-2 in MEFs.CD63 is a biomarker of late endosomes/lysosomes.*

**2.9. Gene delivery via NH_2_-SSN-2 *in vivo***

Given the excellent protection ability in murine plasma, we believed that NH_2_-SSN-2 could silence gene expression *in vivo*. We tested NH_2_-SSN-2/FAM-siRNA complexes in mice. As shown in Figure S11, a strong increase of the FAM green fluorescence was observed after the NH_2_-SSN-2/FAM-siRNA complexes injection. To further estimate the *in vivo* silencing efficiency of NH_2_-SSN-2 delivered siRNA, we evaluated the STING gene knockdown in mice. STING plays a vital role in immunity and inhibiting STING may be potentially therapeutic for inflammatory treatments.^[45, 46]^ As shown in Figure 6a-c, STING protein and mRNA were reduced in white blood cells. Afterwards, the STING mRNA in the various organs was assayed after perfusing by PBS. It is observed that the NH_2_-SSN-2/STING-siRNA complex exhibited a certain degree of accumulation in the liver. Fluorescence images (Figure 6d) further confirmed the liver accumulation of NH_2_-SSN-2. We also estimated the toxicity of NH_2_-SSN-2 by assessing acute toxicity *in vivo*. It demonstrated no significant difference between PBS and NH_2_-SSN-2 treatment groups within 2 weeks after injection. No mice died and no obvious weight loss was observed even though the NH_2_-SSN-2 dose was at 400 mg/kg (Figure S12), which was 20 times higher than its effective knock-down dose *in* *vivo*. The morphology and hematoxylin-eosin (H&E) staining showed that NH_2_-SSN-2 nanocarriers induced no necrosis or apoptosis in major organs under 200 mg/kg (Figure 6e). However, significant tissue edema and infiltration of inflammatory cells appeared in the lung at a dose of 400 mg/kg. It suggested that NH_2_-SSN-2 was an efficient delivery agent and promising for future delivery of siRNA *in vivo*.


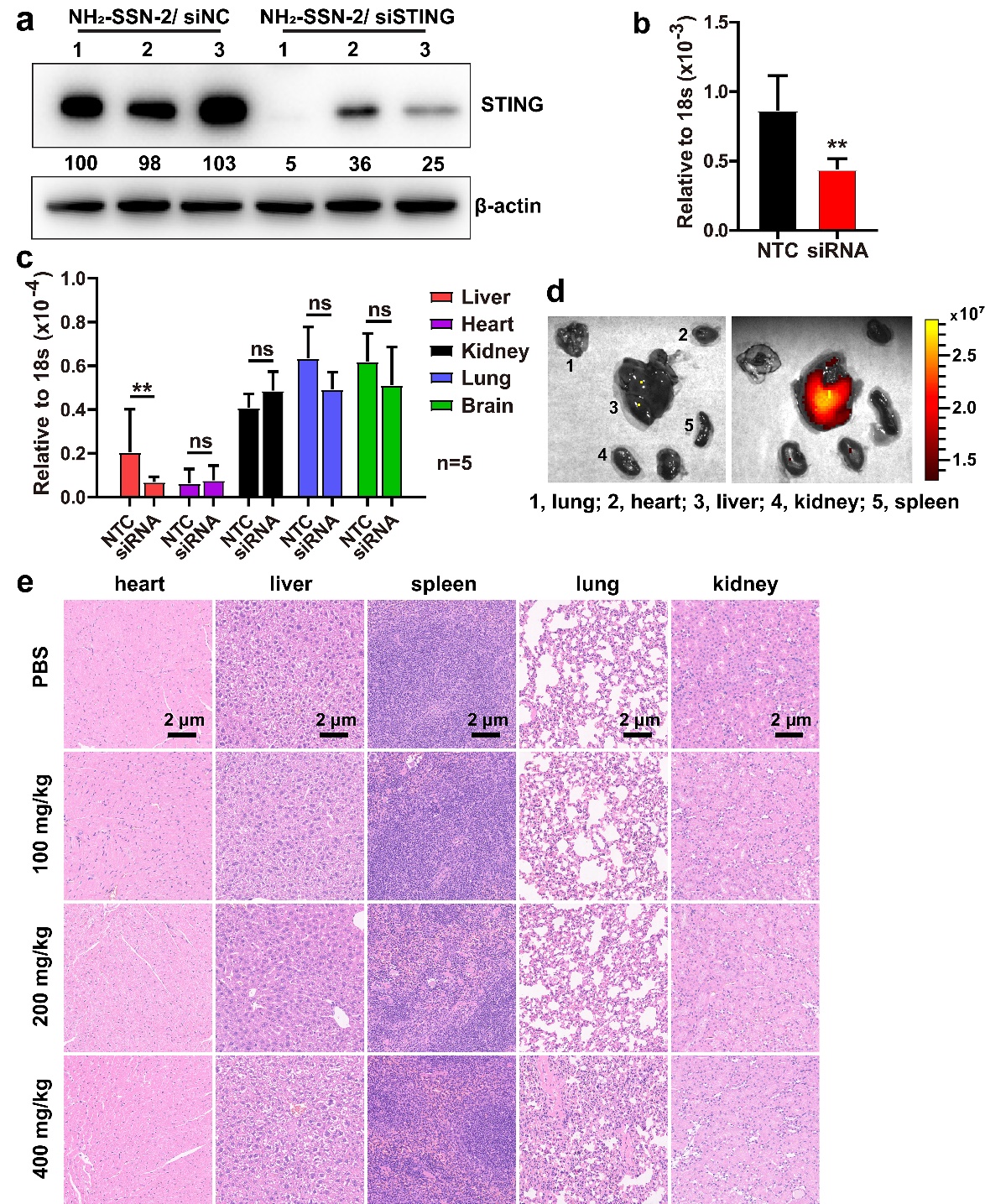


***Figure 6. In vivo siRNA delivery performances.*** *a, STING and β-actin protein levels in white blood cells from 3 individuals. NH_2_-SSN-2 loaded siRNA formulation was intravenously injected. White blood cells were collected from each mouse at 6 h post-injection. 40 μg of cellular lysates were assayed by Western-Blotting. siNC, non-target control siRNA. The grey intensities of GAPDH, hnRNPA2B1, and STING were estimated using Image J and normalized to β-actin. The relative gene expression was labeled. b, In vivo knockdown efficacy of STING gene by NH_2_-SSN-2 based formulation with comparison to NTC. Results were presented as mean ± SD and analyzed by a two-tailed t-test.**, p < 0. 01, n = 3. c, In vivo knockdown efficacy of* *STING gene in various organs. Results were presented as mean ± SD and analyzed by two-tailed t-test, **, p< 0.01; ns, not significant; n = 5. d, Fluorescence images showing the targeting property of NH_2_-SSN-2 towards major organs in mice. e, Representative results for hematoxylin and eosin staining in major organs from mice treated with various doses of NH_2_-SSN-2.*

1. **Conclusion** **and Discussion**

In summary, a series of spiky silica nanoparticles (SSNs) have been successfully fabricated through a one-pot surfactant-free competitive epitaxial growth approach. The obtained nanoparticles exhibit uniform particle size (~200 nm) and most importantly, easily tunable spike lengths. It is first demonstrated that the length of surface spikes can greatly influence siRNA loading and release performance, and longer surface spikes have higher loading and less release of siRNA. Taking advantage of the tunable spike lengths of SSNs, we have demonstrated that amino-modified SSNs with appropriate spike lengths (~40 nm, NH_2_-SSN-2) exhibit an extraordinary siRNA protection capability and siRNA delivery performance both *in vitro* and *in vivo*.

Our studies provided new evidence that the proposed synthetic strategy can efficiently modulate the surface spike structure of silica nanoparticles. Though previous research reported the synthesis of silica nanoparticles with spikes on the surface,^19, 20, 23^ fine control of the spike length remains a challenge due to the lack of efficient strategies. Our proposed fabrication strategy can easily control the relative condensation kinetics of the reactants by controlling the precursors’ adding sequence and delay addition time interval, which eventually controls the length of surface spikes. While for previous studies, the spikes are modulated by reaction time, where the spikes grow very fast at first and difficult to control their spike lengths. Moreover, benefiting from the developed new approaches, it is possible to prepare silica nanoparticles with the controlled length of surface spikes, and it is revealed that the length of surface spikes has a significant impact on RNA delivery efficacy and surface spikes with certain lengths have the best RNA delivery efficacy. Besides, understanding how these nanoparticles are internalized by cells and then processed within the cells is critical for a delivery system. We explored the cellular uptake pathway of NH_2_-SSN-2 via computer simulation and biological approaches. It was found that NH_2_-SSN-2 was internalized through dynamin-dependent endocytosis (Figure 5), which provides the theoretical basis for the subsequent development of new delivery systems. We believe that this study provides an important new step toward the application of siRNAs as therapeutic agents by providing a new delivery platform with enhanced delivery performance, especially *in vivo*. Our results indicate that NH_2_-SSN-2 loaded with siRNA has good clinical translation potential with good stability and biosafety.

1. **Materials and Methods**

4.1.Cell culture and Reagents.

HeLa, HepG2, and 293T cells were cultured in Dulbecco's Modified Eagle Medium(DMEM). B16F10 cells were cultured in RPMI-1640. All culture mediums were supplemented with 10% (v/v) fetal bovine serum (FBS), 5 μg/mL of penicillin and 10 μg/mL of streptomycin. Bone marrow-derived macrophages (BMDMs) were generated as described.^[1]^ Briefly, bone marrow from the tibia and femur was flushed out by PBS and cultured in 10 mL complete medium (DMEM supplemented with 20% heat-inactivated FBS and 20 ng/mL GM-CSF) at 37°C for 7 days. Mouse embryonic fibroblasts (MEFs) were harvested according to the standard protocol.^[2]^ Briefly, the mouse whole embryo (E13.5) was isolated. Heads and viscera were removed. The remaining bodies were washed in PBS, and transferred to 35 mm Petri-dishes, minced with scissors, and digested with 0.25% trypsin/EDTA 1 mM for 5-10 min at 37°C. Following digestion, 1-3 mL DMEM, supplemented with 10% FBS and 5 μg/mL of penicillin and 10 μg/mL of streptomycin, was added. The tissue was pipetted up and down to get a single-cell suspension. All cells were maintained in an incubator with a humidified atmosphere of 5% CO_2_ at 37°C.

Primary antibody hnRNPA2B1 (sc-374053) was purchased from Santa Cruz. The STING (#13647), CD63 (#55051), β-actin (#4970), and glyceraldehyde-3-phosphate dehydrogenase (GAPDH) (#5174) antibodies were purchased from Cell Signaling Technology (Denver, MA, USA). The secondary antibody goat antirabbit (111-035-003) and goat anti-mouse (115-035-003) were purchased from Jackson ImmunoResearch (West Grove, PA, USA). The fluorescence secondary antibody, Alexa Fluor plus 488 (A-11008), the Lipofectamine 3000, and RNAiMAX were purchased from Thermo Fisher Scientific (Carlsbad, CA, USA). The 2-deoxy-d-glucose (2DG), chloroquine, dynasore, Methyl-β-cyclodextrin (MβCD), cytochalasin dand amiloride (EIPA) were purchased from Titan Scientific Co.,Ltd (Shanghai, China). The 7-keto-cholesterol (7-KC) was purchased from Tsbiochem Scientific Co.,Ltd (Shanghai, China). The Resazurin cell viability kit was purchased from Thermo Fisher Scientific (Carlsbad, CA, USA). VECTASHIELD antifade mounting medium with DAPI was purchased from Vector Laboratories (Burlingame, CA, USA). The cell plasma membrane staining kit with DiI (1,1'-dioctadecyl-3,3,3',3'-tetramethylindocarbocyanine perchlorate) was purchased from Beyotime Biotechnology (Shanghai, China). All kits were used following the manufacturer's instructions.

The following primer pairs were used in real-time quantitative PCR as listed. For mouse STING gene, 5’-TCAGTGGTGCAGGGAGCCGA-3’ (F) and 5’-CGCCTGCTGGCTGTCCGTTC-3’ (R); For mouse 18S ribosomal RNA gene, 5’-ATTGACGGAAGGGCACCACCAG-3’ (F) and 5’-CAAATCGCTCCACCAACTAAGAACG-3’ (R). The siRNA sequences used in this study were listed as follows. 5’-CCACAGAAGAAAGTTTGAGTT-3’ for mouse hnRNPA2B1. 5’-CTTTGGTGGTAGCAGGAAC-3’ for human hnRNPA2B1. 5’-CCAACAGCGUCUACGATT-3’ for mouse STING. 5’-UGACCUCAACUACAUGGUUTT-3’ for GAPDH. The nonsense sequence 5’-TTCTCCGAACGTGTCACGT-3’ was used as a non-target siRNA. For FAM-labeled siRNA (FAM-siRNA), FAM was conjugated with nonsense RNA at the 3′-end. siRNA targeting EGFP is a kindly gift from Dr. Lin.

4.2.The synthesis of SSNs with various spike lengths

SSN-1 was synthesized via a facile one-pot self-assembly co-condensation process. In a typical synthesis, 2 mL tetraethyl orthosilicate (TEOS) was added to an aqueous solution composed of ethanol, distilled water, ammonium hydroxide, and ethylenediamine (EDA). Afterwards, 3-aminophenol (AP) and formaldehyde (F) solution were added to the above solution. After reaction for 5 h, the product was harvested by centrifugation. Finally, SSN-1 was obtained after calcination in air. For the synthesis of SSNs with various spike lengths, TEOS was firstly added to the aqueous solution. After various time intervals (5 min for SSN-2, 10 min for SSN-3, 20 min for SSN-4), 3-aminophenol and formaldehyde were then added to the reaction solution. After centrifugation, drying, and calcination in air, SSN-2, SSN-3, and SSN-4 could be prepared.

4.3.The synthesis of large pore dendritic mesoporous silica nanoparticles (DMSNs)

DMSNs were prepared according to the reported literature with minor modifications.^[3-4]^ In a typical synthesis, 0.14 g of triethanolamine was added to 50 mL water, which was stirred at 80°C in an oil bath for 15 min. Afterwards, 336 mg sodium salicylate (NaSal) and 760 mg cetyltrimethylammonium bromide (CTAB) was added to the above solution. The solution was continuously stirred for another hour. Then, 5 mL of TEOS was added to the above solution and DMSNs were harvested by centrifugation after 2 h of reaction. Finally, the DMSNs were calcined at 550°C in air to remove the surfactants.

4.4.Grafting of amino group on silica nanoparticles

Four milligrams of silica nanoparticles were dissolved in 1 mL ethanol solution and sonicated for 15 min. Afterwards, (3-aminopropyl) triethoxysilane (APTES) was added to the above ethanol solution and stirred for 24 h. The amino-modified silica nanoparticles were collected by centrifugation and washed with ethanol several times. The amino-modified silica nanoparticles were named as NH_2_-SSN-1, NH_2_-SSN-2, NH_2_-SSN-3, NH_2_-SSN-4, NH_2_-DMSN for SSN-1, SSN-2, SSN-3, SSN-4, and DMSNs, respectively.

4.5.Theoretical calculation of the surface spike length of SSNs

The relationship between the surface spike length and the delay addition time interval of 3-aminophenol and formaldehyde solution were theoretically calculated for the synthesis. During the theoretical calculation, we made the following assumptions: (1) TEOS were added to the reaction solution at the time point of “0”, 3-aminophenol and formaldehyde solution was added to the reaction solution at the time point of “t”. (2) In total, the number of nanoparticles yielded in the reaction solution was “N” regardless of the delay addition time interval and it kept consistent during the synthesis. (3) During the reaction, the silica spikes grow vertically on the surface of the *in situ* generated silica cores and the structure of the spikes was simplified as uniform cylinder structures. Based on the above assumptions, the relationship between the total amount of Si (M) and the consumed amount (m (t)) to generate silica cores are calculated by rate reaction equation, which can be written as:

$\left\{\begin{matrix} \frac{dm(t)}{dt}=k(M-m(t)) \\ m(0)=0 \end{matrix}\right.$ (1)

m (t) can be derived from Eq. 1 as:

$m(t)=M(1-e^{-kt})$ (2)

where k is a constant.

The radius of the silica core particles are “R” with the material density of “ρ”, therefore, $m(t)=N\cdot\frac{4\pi}{3}R^{3}\rho$ (3)

This yields for the average radius of the nanoparticles:

$R=\left(\frac{3m(t)}{4\pi\rho N}\right)^{\frac{1}{3}}$ (4)

Since the total Si amount in all the spikes is:

$M(s)=M-m(t)$ (5)

The number of spikes in each nanoparticle is calculated as:

$N(s)=N\cdot\frac{4\pi R^{2}}{\mu}$ (6)

Where µ is the distribution density of spikes on the core surface.

Furthermore, the mass of each spike is calculated as:

$m(s)=\frac{M(s)}{N(s)}=\frac{M-m(t)}{\frac{N\cdot4\pi R^{2}}{\mu}}$ (7)

Afterwards, the length of spikes can be calculated from Eq. 7 as:

$l(t)=\gamma\cdot\frac{M-m(t)}{\frac{N\cdot4\pi R^{2}}{\mu}}=\frac{\gamma\mu(M-m(t))}{N\cdot4\pi R^{2}}$ (8)

Where γ is the ratio between the mass and length of a spike.

Furthermore, the length of the surface spikes can be calculated as:

$l(t)=C\cdot\frac{e^{-kt}}{(1-e^{-kt})^{\frac{2}{3}}}$ (9)

Where C is a constant, and $C=\frac{\gamma\mu}{4\pi N\left(\frac{3M}{4\pi\rho N}\right)^{\frac{2}{3}}}$,

With input the measured data from Figure S1, *C* is calculated to be 1.522, and *k* is calculated to be 0.003157. Therefore, the length of the surface spikes can be calculated as:

$l(t)=1.522\cdot\frac{e^{-0.003157t}}{(1-e^{-0.003157t})^{\frac{2}{3}}}\text{，0}\leq\text{t}\leq\text{60 min}$ (10)

4.6. Characterizations of SSNs

TEM measurements were conducted on a JEOL-1010 (Tokyo, Japan) microscope. Micromeritics Tristar 3000 system was used to characterize the nitrogen adsorption/desorption experiments at 77 K. Before the nitrogen adsorption/desorption measurements, samples were pre-treated under a vacuum line at 120 °C for 12 h. Specific surface areas were calculated by the Brunauer-Emmett-Teller (BET) method through the adsorption data at relative pressure (P/P_0_) range of 0.05-0.35. Pore-size distribution curves and the total pore volumes of the samples were obtained by the Barrett-Joyner-Halenda (BJH) method through the adsorption branches of the isotherms. Furthermore, the total pore volume was calculated from the amount of nitrogen adsorbed at the relative pressure (P/P_0_) of 0.99.

4.7.Cell transfection

NH_2_-SSNs were dissolved in diethylpyrocarbonate (DEPC)-treated water at the final concentration of 10 mg/mL. In separate tubes, 20 µL of dissolved NH_2_-SSNs and 100 pmol of siRNA were diluted in 100 µL serum-free medium, respectively. The diluted siRNA was added to the diluted NH_2_-SSNs and incubated at room temperature for 15 minutes. Then 200 µL mixture was added to the culture medium, respectively. The commercial siRNA transfection reagent was used following the manufacturer’s instructions.

For repeated treatment, MEF and 293T cells were transfected with NH_2_-SSN-2/siRNA, Lipo3000/siRNA, and RNAiMAX/siRNA, respectively. Afterwards, transfected cells were cultured and divided into 3 equal parts every 48 h. One part was repeatedly transfected. The left two parts were used to evaluate gene knockdown efficiency and cell viability. For cell viability assay, cells were incubated for 4 h with 1 mg/mL resazurin solution (10 µL), and then the fluorescence intensity was measured using a SpectraMax M3 microplate reader (Molecular Devices, San Jose, CA, USA) at an excitation wavelength of 544 nm and emission wavelength of 595 nm.^[5]^

To estimate gene delivery efficiency, transfected cells were evaluated with flow cytometry analysis performed by a BD FASC Aria II flow cytometer (BD Biosciences, San Diego, CA, USA). A total of 10^4^ gated events were acquired per sample. Data were analyzed with FlowJo_V10 software (10.5.4).

4.8.Microscopy assay

MEF, HeLa, HepG2, 293T, and BMDM cells were seeded in 6-well plates (1×10^5^ cells per well) for 12 h. A concentration of 100 μg/mL NH_2_-SSNs/FAM-siRNA was added and incubated with the cells for 0 h, 2 h, and 6 h, respectively. After washing 3 times to remove nonbinding particles, cells were fixed with 4% paraformaldehyde and visualized on ZEISS Vert.A1 fluorescence microscope (Carl Zeiss, Heidenheim, Germany).

4.9.Immunofluorometric assay

MEF and HepG2 cells were grown on coverslips for 12 h. After treatment with NH_2_-SSNs, cells were washed with PBS and fixed with 4% paraformaldehyde. The cells were permeabilized in 0.5% v/v Triton X-100 in PBS and blocked by 5% bovine serum in PBS. Then, the cells were incubated with the primary antibodies (1:100) overnight and incubated with secondary antibodies (1:2000) for 60 min. After being sealed with the VECTASHIELD mounting medium with DAPI cells were visualized on the Leica TCS SP8 STED confocal microscope (Leica Microsystems, Weztlar, Germany). Images were analyzed by ImageJ and ZEN Imaging Software.

4.10.RT-PCR and quantitative PCR analysis

Total RNA was isolated using TRIzol reagent (Takara, Japan) according to the manufacturer’s instructions. One microgram of total RNA was converted into cDNA with random primers and Superscript III reverse transcriptase (Takara, Japan). PCR was performed with gene-specific primer sets. Quantitative real-time PCR was performed with SYBR green (Roche, Switzerland) incorporation on the LightCycler® 96 System (Roche, Switzerland). 18S rRNA was used as internal control, and the data were presented as accumulation index (2^-△△Ct^).^[6]^

4.11.Gel shift assay

NH_2_-SSNs (100 μg) were mixed with the indicated amount of siRNA. The mixture was incubated for 30 min at RT, mixed with loading dye, and submitted to gel shift assay. The siRNA binding ability of NH_2_-SSNs was measured by gel shift assay as previously described. The polymer/siRNA ratios were electrophoresed in a 2% agarose gel containing GeneGreen at 35 V in the TAE solution.^[7]^ For the protection ability analysis, peripheral blood was isolated from healthy C57BL/6 mice and centrifuged at 12000×g for 5 min to collect plasma. The naked siRNA or siRNA/NH_2_-SSN-2 mixture was incubated with plasma at 37°C and then submitted for gel shift assay. Data quantification was performed using ImageJ.

4.12.Western-Blotting

Tissues or cells were homogenized in lysis buffer (BioRad, Hercules, CA, USA) with proteinase and phosphorylase inhibitor cocktail (Thermo Fisher Scientific, Carlsbad, CA, USA), centrifuged for 15 min (12,000 rpm, 4°C). The protein concentration was determined using the BCA Protein Assay Kit (SolarBio, Beijing, China). Twenty micrograms of total protein were subjected to SDS-PAGE and transferred onto PVDF membranes. The primary antibodies were diluted in TBST buffer following the manufacturer's instructions and incubated with membranes overnight at 4°C. The HRP-conjugated secondary antibodies were diluted at 1:1000. Membranes were visualized by using the ECL Western-Blotting reagent (Tanon, Shanghai, China). The gel was normalized by ImageJ software (National Institutes of Health, USA )

4.13.Animal assay

All animal experiments were undertaken in accordance with the National Institute of Health Guide for the Care and Use of Laboratory Animals, and all procedures were approved by the Committee of Experimental Animals of the Ocean University of China. C57BL/6 mice were purchased from Beijing Vital River Laboratory Animal Technology Co (Beijing, China). To generate xenograft models, B16F10 cells (1×10^5^ cells/injection) were inoculated via subcutaneous injection into 6-week-old C57BL/6 male mice. On day 11 after injection, mice were given an intra-tumoral injection of PBS (control), FAM-siRNA, and FAM-siRNA-loaded NH_2_-SSNs, respectively. After 6 h, the mice were euthanized. Tumors were dissected, frozen sliced, and observed under a ZEISS Vert.A1 fluorescence microscope (Carl Zeiss, Heidenheim, Germany).

For STING knockdown *in vivo*, the 6-week-old C57BL/6 male mice were randomly divided into two groups and intravenously injected with 200 μL PBS (control), and STING siRNA (4 μg) loaded NH_2_-SSNs, respectively. After 24 h, mice were euthanized and the blood was collected. Subsequently, the red blood cell was removed by Red Blood Cell Lysis Buffer (R1010, SolarBio, Beijing, China) for 15 min. After centrifugation at 2,000 rpm, the white blood cell was collected. The heart, liver, lung, kidney, and brain were collected after perfusing by PBS. Total RNA was isolated using TRIzol reagent and the STING RNA level was evaluated by qRT-PCR.

For biodistribution of NH_2_-SSN-2, Cy5-siRNA(100 pmol) loaded NH_2_-SSN-2 (200 μg) in 200 μL of PBS was administrated intravenously via the tail vein. After 1 hour, the mice were sacrificed. The major organs including heart, liver, spleen, lung and kidney were collected and visualized by using the Lumina IVIS III Imaging System (PerkinElmer, Waltham, MA, USA) at excitation = 620 nm and emission = 670 nm.

For cytotoxicity and biocompatibility of NH_2_-SSN-2 *in vivo*, the 6-week-old C57BL/6 male mice were intravenously injected with 200 μL PBS (control) and NH_2_-SSN-2 (final concentration is 100 mg/kg, 200 mg/kg and 400 mg/kg). The body weight was measured every day. After 14 days, the mice were euthanized and the major organs were collected in 4% Paraformaldehyde. The H&E staining was supported by Serivebio (Wuhan, China).

4.14.Modeling the spiky silica nanoparticle structure

To prepare an atomistic miniature model of a spiky silica nanoparticle (diameter 4-5 nm), the structures of the initiator and terminator end groups and the monomer unit were sketched using the structure of orthosilicic acid. The polymer chains of 1-5 nm were grown using the Polymer Builder tool of Schrödinger’s Materials Science Suite (Schrödinger, LLC, New York, NY, 2021). The repeating units of this polymer structure were created along the X, Y and Z axis using the 3D Builder Panel. Using the Nanoparticle Builder Panel, a 1-2-nm spherical nanoparticle was created. The silica atoms of the orthosilicic acid molecules were bridged with each other via the oxygen atoms of the hydroxyl groups (Si-O-Si). The terminal hydroxyl groups (Si-OH, silanol groups) were left on the surface of the nanoparticles. At specified distances (1.1-1.2 nm), 8 spike-like projections of orthosilicic acid were created by sketching. To create the amino-functionalized SSN-2, 2-aminoethyl silane groups were grown over the surface of the non-functionalized SSN-2 by covalent linking to silanol groups on the outer surface. The clashes between Si-O-Si and Si-OH atoms were removed by energy minimization of the structure using the 3D Builder Panel at each step.

4.15.Molecular Dynamics Simulations

To prepare a simulation system of an SSN-2 with our different types of lipid bilayers, i.e., 1-Palmitoyl-2-oleoyl-sn-glycero-3-phosphatidylethanolamine (POPE), 1-Palmitoyl-2-oleoyl-sn-glycero-3-phosphatidylcholine (POPC), 1,2-Dioleoyl-sn-glycero-3-phosphatidylcholine (DMPC), 1,2-dipalmitoyl-sn-glycero-3-phosphocholine (DPPC), the System Builder panel of the Desmond module was used (Schrödinger Release 2021-4: Desmond Molecular Dynamics System, D. E. Shaw Research, New York, NY, USA, 2021. Maestro-Desmond Interoperability Tools, Schrödinger, New York, NY, USA, 2021).^[8]^ The position of the membrane was adjusted, keeping a distance of 20 Å from the apex of the SSN-2 to the C2 atoms of the membrane. For each system, an orthorhombic unit cell of 20x20x20 Å^3^ box size and periodic boundary conditions (PBC) were used.

Each SSN-2-membrane containing system was submitted to a 100-ns molecular dynamics (MD) simulation. The simulations were performed in the OPLS4e force field using the Molecular Dynamics Panel of Desmond.^[9]^ The simulation systems were relaxed using a 6-stage membrane relaxation protocol. Briefly, the relaxation protocol involved 100 ps of Brownian dynamics (BD) at 10 K to remove steric clashes, followed by a short 20-ps BD simulation at 100 K and 1000 bar pressure in the NPT ensemble with a water barrier and the membrane restrained in the Z direction. In the third stage, a 100-ns MD simulation at 100 K was carried out using a water barrier and the NPγT ensemble with restrains on the membrane. In the fourth stage, the system was heated from 100 K to 300 K in the NPγT ensemble with a gradual release of restraints for 150 ps. In the fifth stage, a 50-ns MD simulation in the NVT ensemble was performed with restrained heavy atoms at 300 K, followed by another 50-ps simulation in the final stage without any restraints. The production simulations were then performed for 100 ns at 300 K and 1.01325 bar with the Nosé-Hoover chain thermostat^[10-12]^ and barostat using the Martyna-Tobias-Klein method with isotropic coupling in the NPγT ensemble.^[13]^ The Coulombic method used for long-range interactions was U-series while the cut-off radius for short-range interactions was set to 9.0 Å.^[14]^ Lipid density analysis was carried out using the trajectory density analysis tool, and the orientation and alignment of lipid molecules in the bilayer were determined by calculating the tilt angle with the surfactant tilt angle calculation tool of Schrödinger’s Materials Science Suite (release 2021-4). The total energy and distance calculations were carried out by using Schrödinger’s Materials Science workspace tools. The results were further analyzed using Microsoft Office 365 tools.

4.16.Statistical Analysis

All experiments were performed in triplicates and results were presented as means ± SD, and the p-value was calculated using GraphPad Prism 8.0 software (GraphPad Software Inc., San Diego, CA, USA) by the Student’s unpaired two-tailed t-test and one-way ANOVA analysis. A p-value less than 0.05 was considered statistically significant.

**Acknowledgments**

**General**: Authors acknowledge the support from Instrumental Analysis Center of Shenzhen University (Xili Campus). Biocenter Finland Bioinformatics network, CSC IT Center for Science, Tor, Joe and Pentti Borg Memorial Fund and Prof. Mark Johnson and Dr. Jukka Lehtonen are gratefully acknowledged for the excellent computational infrastructure at the Åbo Akademi University.

**Author Contributions:** J. Fu, W. Han, M. Qiu, X. Wang and H. B. Zhang conceived the experiments. J. Fu, W. Han, Y. Sun, X. Zhang, L. Li, L. Xia and B. Wei performed the experiments and conducted the characterizations. R. Bhadane, J. Rosenholm and O. Salo-Ahen performed the molecular dynamics simulations. L. Xia, T. Fan, B. Zhang, S. Wageh, A. Al-Ghamdi and H. B. Zhang, H. Zhang contributed to the discussion of the results. J. Fu, M. Qiu, X. Wang, H. B. Zhang, H. Zhang, and L. Yu provide financial support.

**Funding:** This work was supported by National Natural Science Foundation of China (U22A20582, X.W.; 22005197, J.F.; 61435010 and 61575089, H.Z.; U1803128, M.Q.; 81991525, X.W.; 81871472, H.Z.), Natural Science Foundation of Shandong Province (ZR202110150015, ZR2021LSW013, ZR202110290057), China Postdoctoral Science Foundation (Grant No. 2021M692197, J.F.), State Key Research Development Program of China (2019YFB2203503, H.Z.), the Science and Technology Innovation Commission of Shenzhen (KQTD2015032416270385, JCYJ20150625103619275, H.Z.; JCYJ20180305124854790, M.Q.), the Natural Science Foundation of Guangdong Province (2018A030310500) and the Taishan Scholar Project (tsqn201909054, tsqn201909170), the Fundamental Research Funds for the Central Universities to X.W. and M. Q., and Tor, Joe and Pentti Borg Memorial Fund (R.B.). This project was funded by the Deanship of Scientific Research (DSR) at King Abdulaziz University, Jeddah, under grant no. (KEP-MSc-70-130-42). The authors, therefore, acknowledge with thanks DSR for technical and financial support. The Research Fellow (Grant No. 328933), Solutions for Health Profile (336355), and InFLAMES Flagship (337531) projects from Academy of Finland, as well as the Finland-China Food and Health (FCFH) International Pilot Project funded by the Finnish Ministry of Education and Culture are acknowledged.

**Conflicts of Interest**

The authors declare that there is no conflict of interest regarding the publication of this article.

**Data Availability**

Data supporting the findings of this study are available in the main text or the supplementary information.

Supplementary Materials


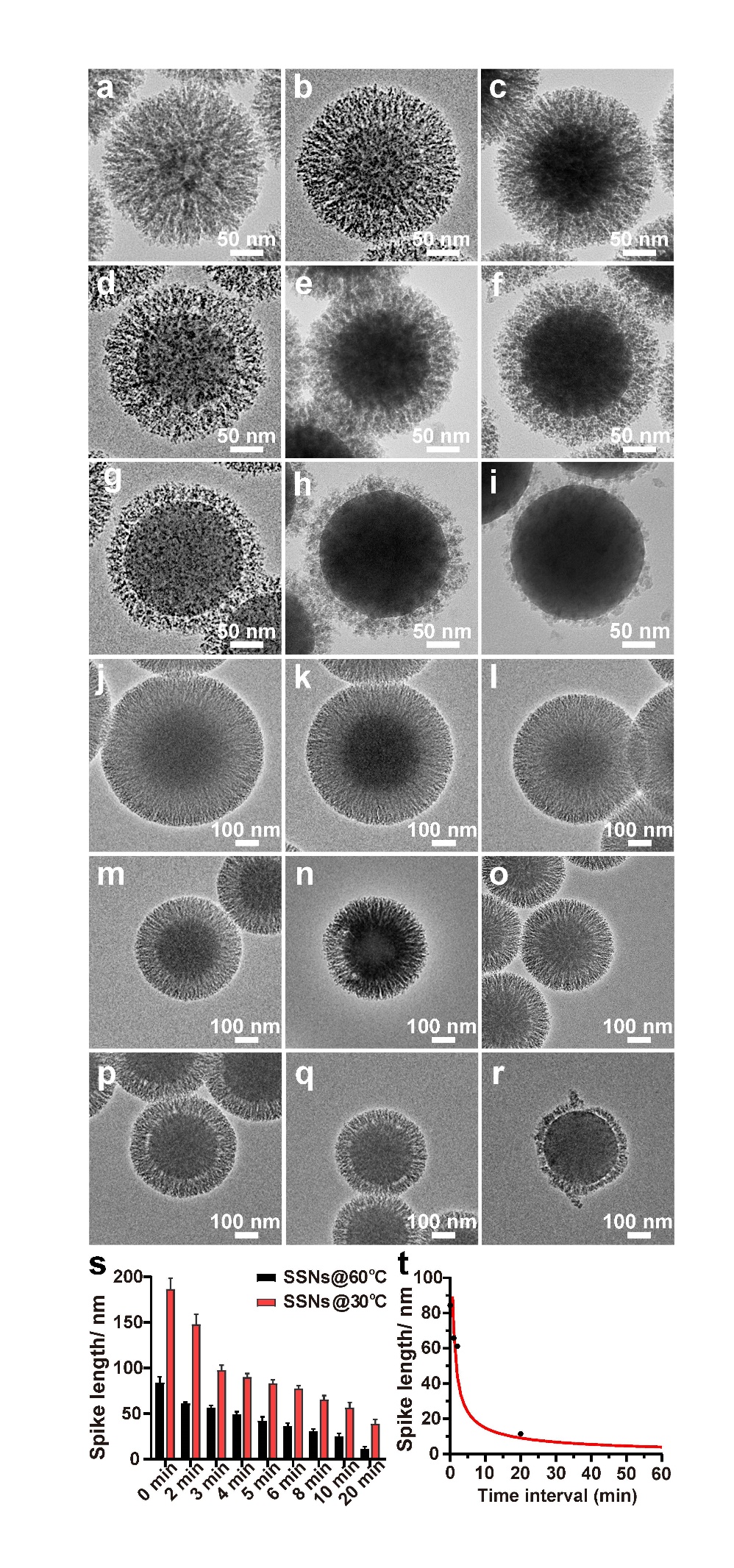


***Figure S1.*** *Fine control of the surface spike length of SSNs. a-i,* *TEM images of SSNs synthesized at 60°C under various time intervals. 0 min (a, SSN-1), 2 min (b), 3 min (c), 4 min (d), 5 min (e, SSN-2), 6 min (f), 8 min (g), 10 min (h, SSN-3) and 20 min (i, SSN-4). j-r, TEM images of SSNs synthesized at 30°C under various time intervals. 0 min (j), 2 min (k), 3 min (l), 4 min (m), 5 min (n), 6 min (o), 8 min (p), 10 min (q) and 20 min (r). Statistical results of the surface spikes length of SSNs synthesized under 30°C and 60°C with various time intervals (s). Results were presented as mean ± SD, n = 10. Theoretical calculation of the surface spike lengths with respect to the delay addition time intervals (t) (equation 10 in the experimental section), red curve, and experimental statistical results of the surface spikes length of SSNs synthesized under 60°C with various time intervals of o min, 1 min, 2 min and 20 min (black dots).*


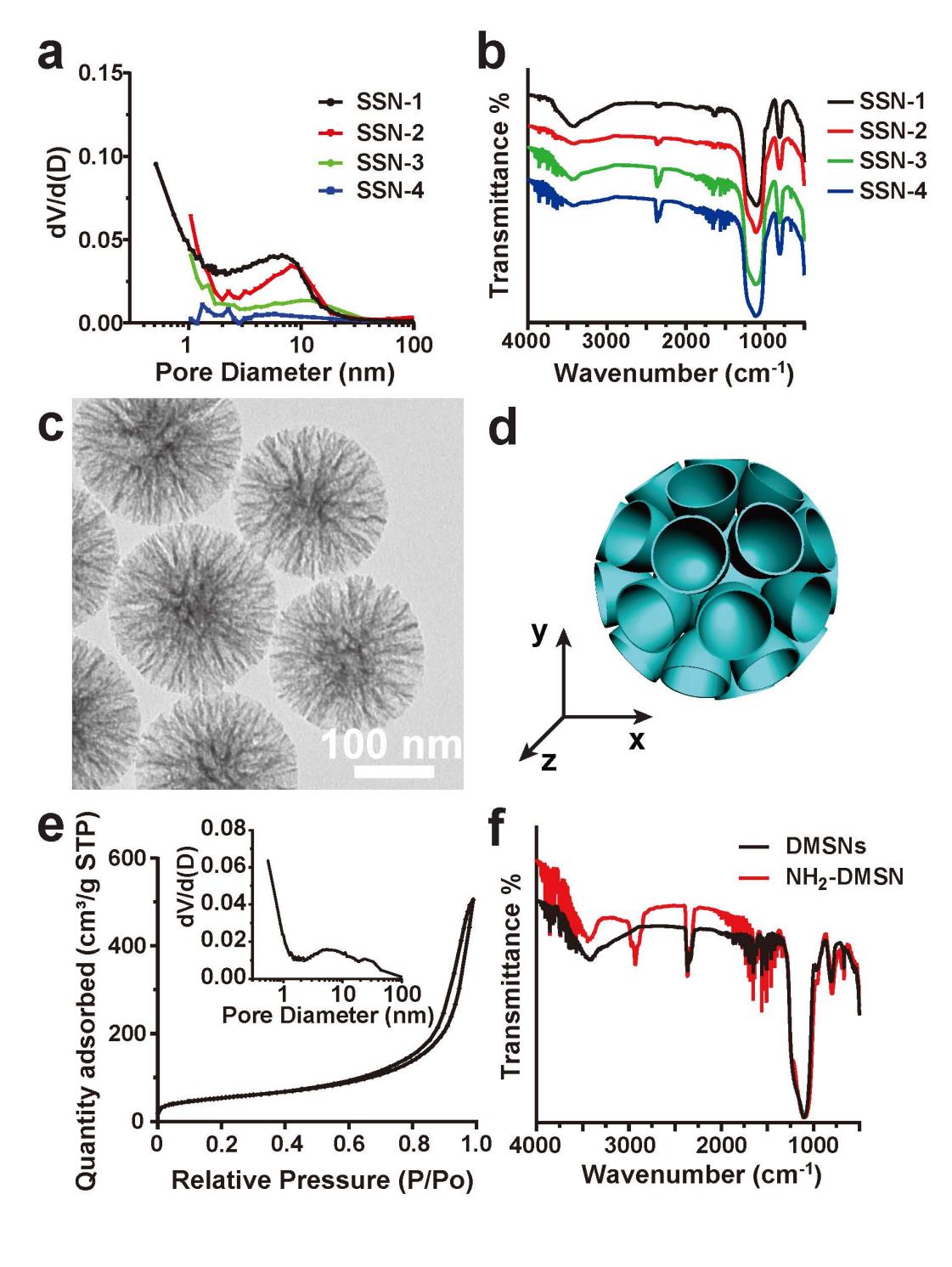


***Figure S2.*** *FTIR spectrum (a) and pore size distribution curves (b) of SSNs. Structural characterizations of the DMSNs (c-f). TEM image (c), 3D model (d), Nitrogen adsorption-desorption isotherms and pore size distribution curves (inset) (e), FTIR spectrum (f).*


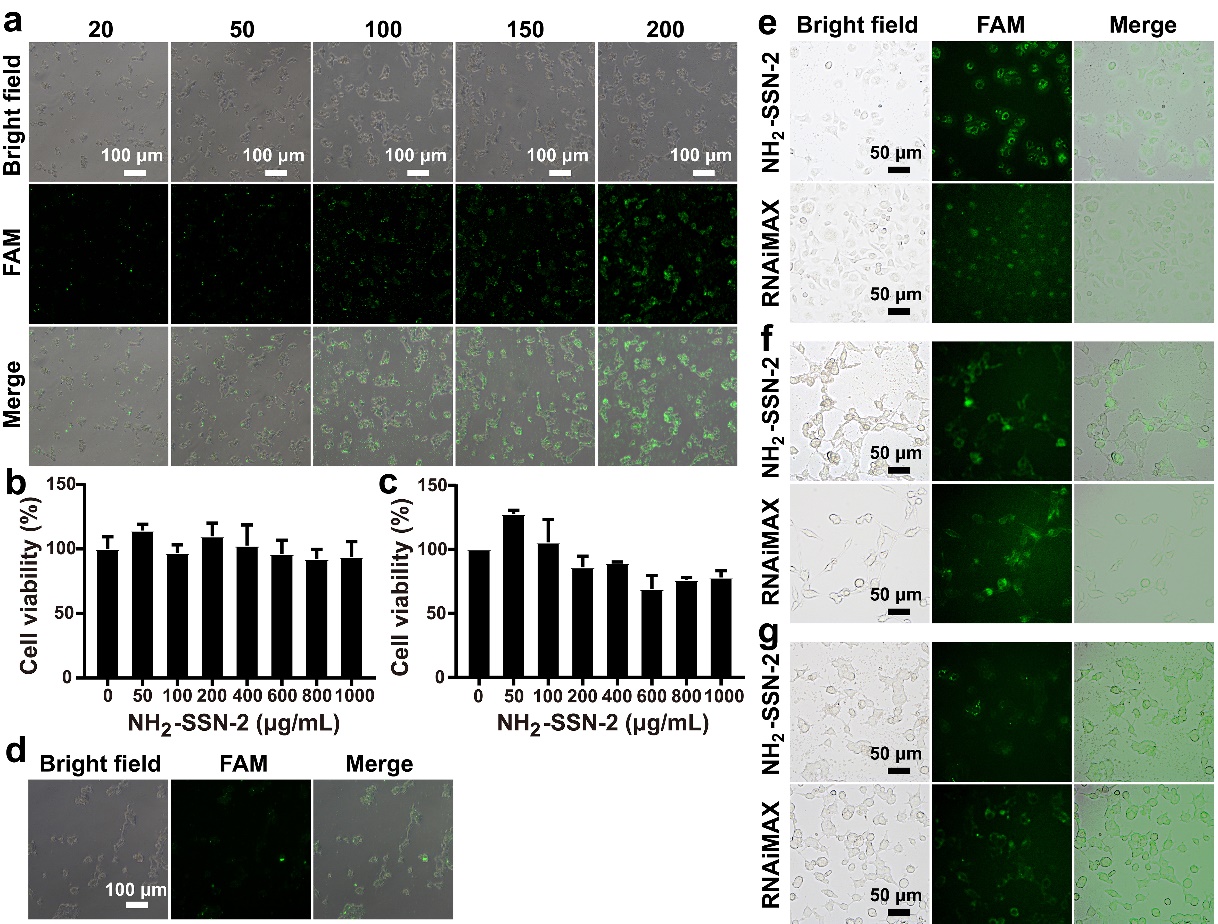


***Figure S3.*** *Fluorescence images of cellular uptake of SSNs. Various amounts (20 to 200 µg/mL) of NH_2_-SSN-2/FAM-siRNA complexes treated HepG2 cells after 6-hour incubation (a). Cytotoxicity assay for NH_2_-SSN-2 treated MEF cells (b) and HEK-293T cells (c). NH_2_-DMSN/FAM-siRNA transfected HEK-293T cells after 6-hour incubation (d). FAM-siRNA transfection using NH_2_-SSN-2 or RNAiMAX in BMDMs (e), MEFs (f), and HEK-293T cells (g) after 6-hour incubation. Results were presented as mean ± SD, n = 3.*


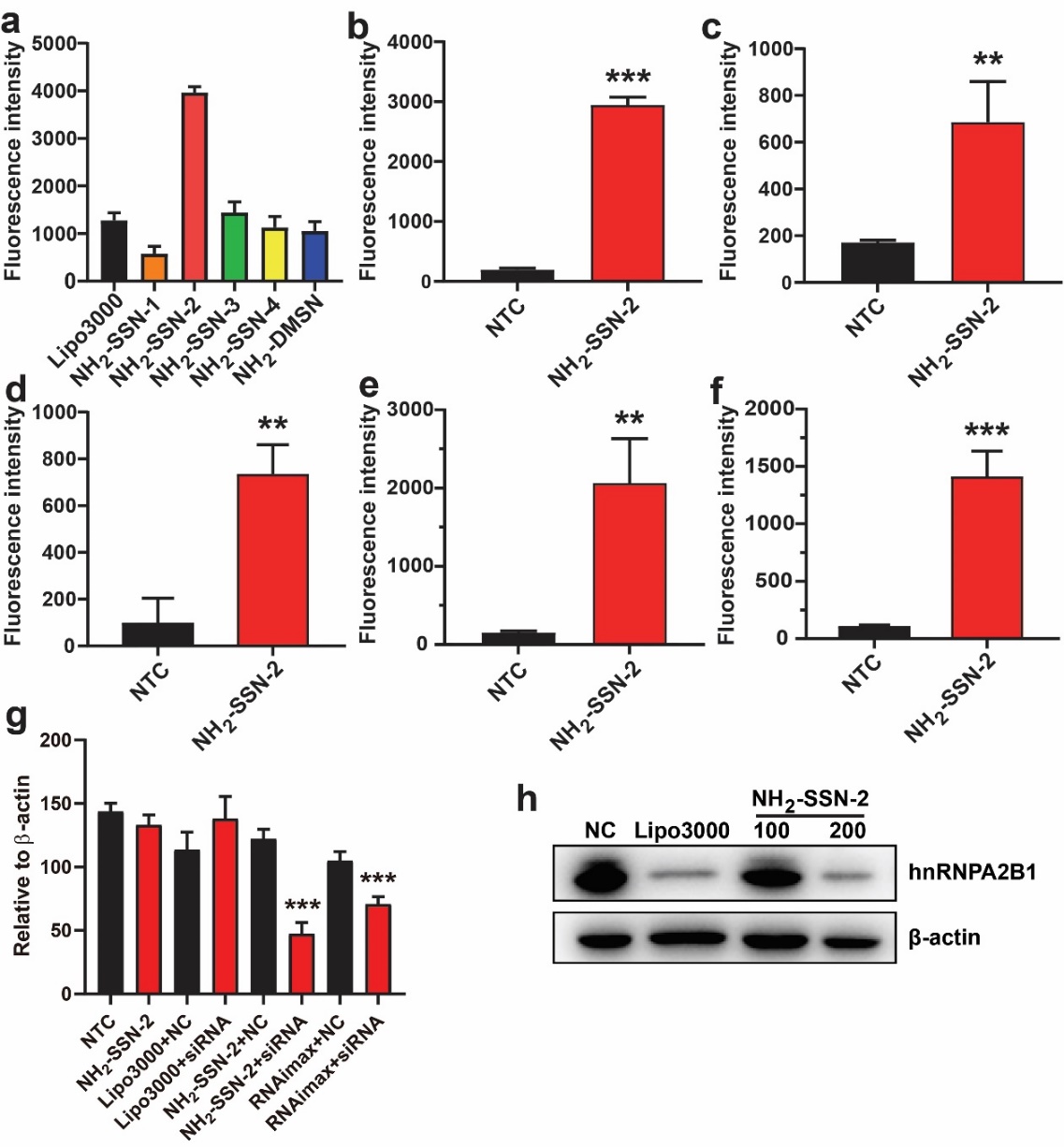


***Figure S4.*** *Evaluation of the fluorescence intensity of various formulations in HEK-293T cells (a). Nucleic acid delivery performance of NH_2_-SSN-2/FAM-siRNA complex in HeLa (b), A549 (c), HCT116 (d), BMDM (e), and MEF cells (f). Quantified measurement of the grey values of the Western-Blotting results in Figure 2d (g). Relative hnRNPA2B1 and β-actin protein levels in A549 cells (h). Cellular lysates were assayed by Western-Blotting using indicated antibodies. Non-treatment control (NC, lane 1), Lipo3000 combination with hnRNPA2B1-siRNA (lane 2), various amount of NH_2_-SSN-2 loaded with hnRNPA2B1-siRNA, 100 µg/well (lane 3) and 200 µg/well (lane 4). Results were presented as mean ± SD, n=3, and analyzed by one-way ANOVA. *p < 0.05,**p < 0.01，***p < 0.001.*


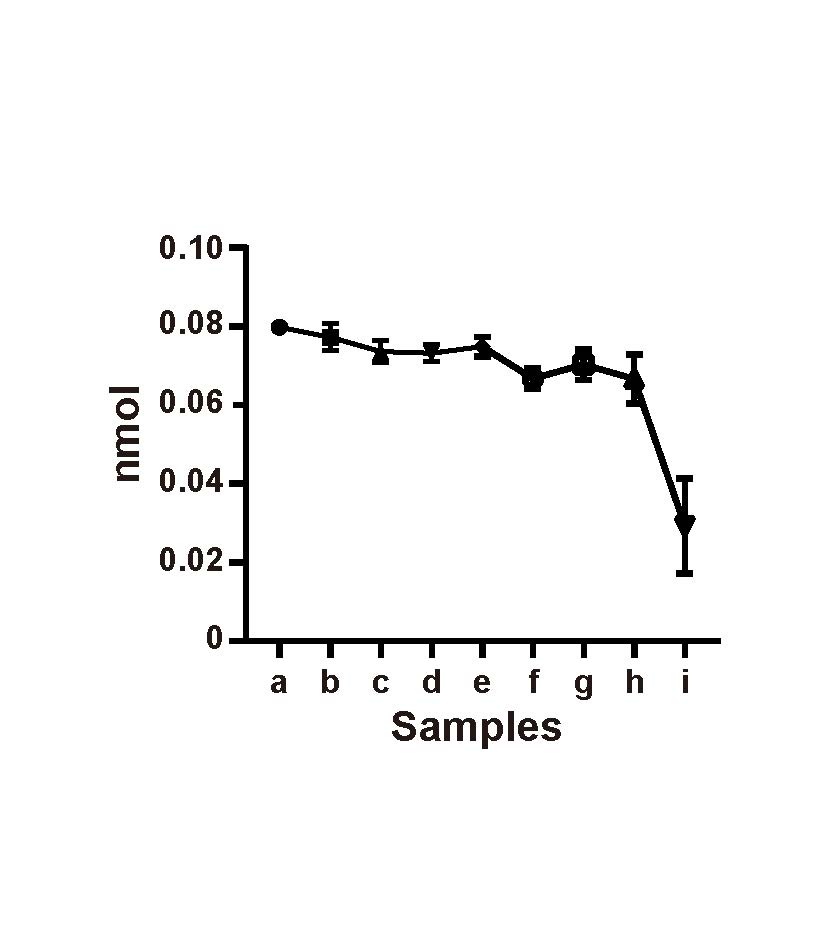


***Figure S5.*** *siRNA binding performances of NH_2_-SSNs with different spike lengths. Quantitative measurement of the siRNA loading capability of SSNs in Figure S1a-i. Results were presented as mean ± SD, n = 3.*


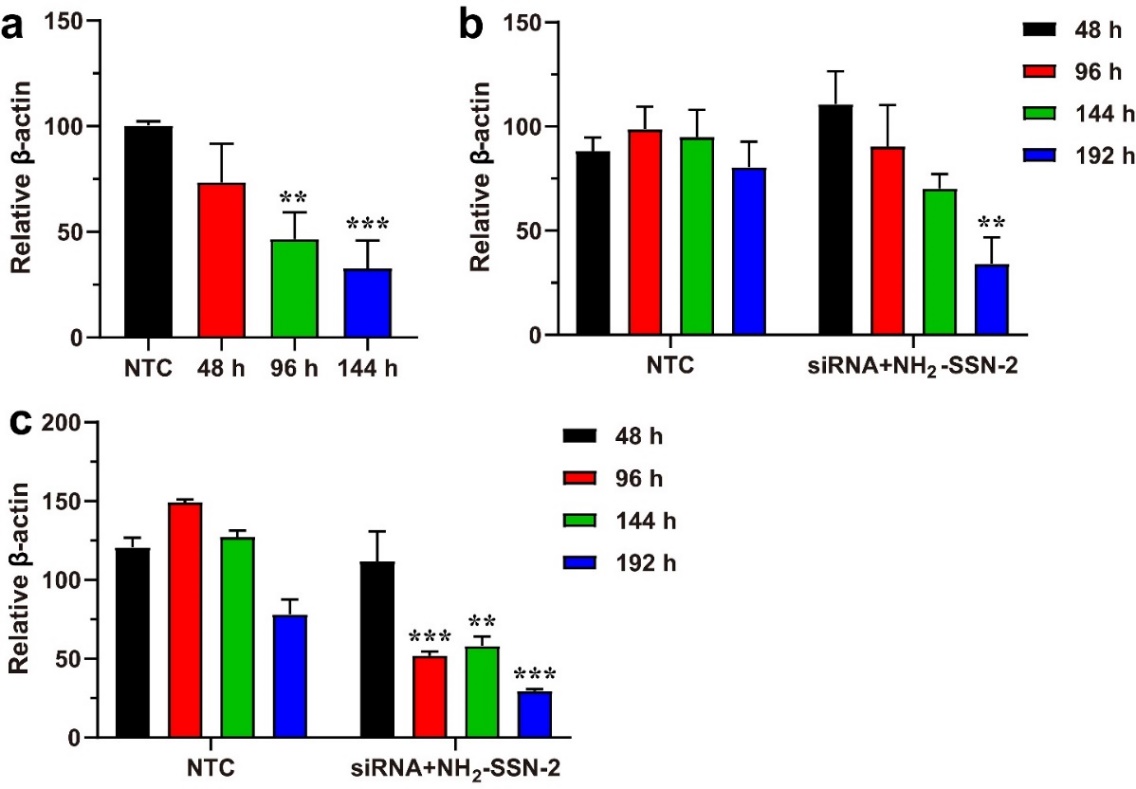


***Figure S6.*** *Quantified measurement of the grey values of the Western-Blotting results in Figure 3d-f. Relative target protein to β-actin protein levels in MEF cells after repeated treatment, GAPDH (a), hnRNPA2B1 (b) and STING (c).* *Results were presented as mean ± SD and analyzed by one-way ANOVA. *p < 0.05,**p < 0.01，***p < 0.001.*


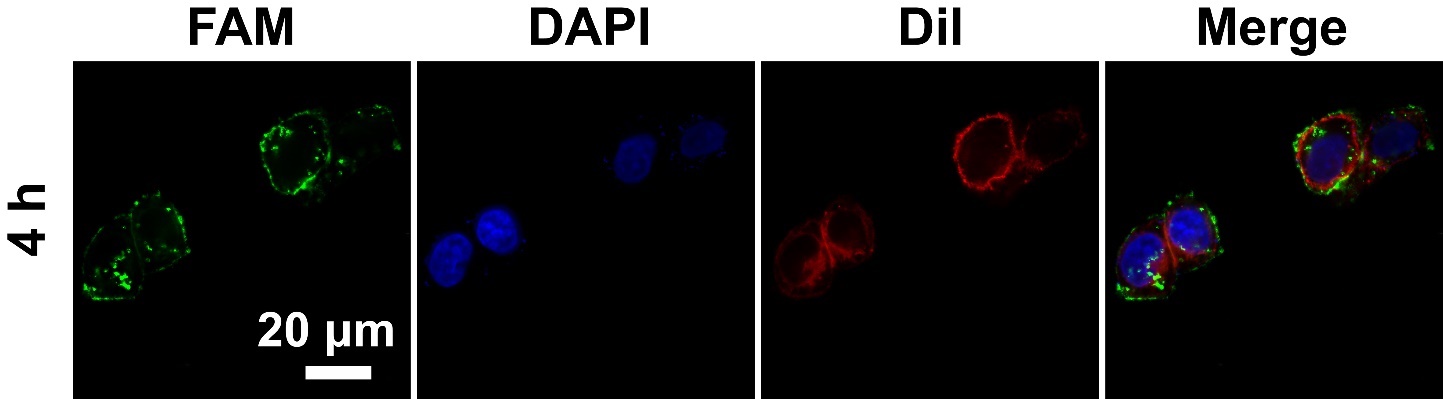


***Figure S7.*** *Fluorescence images of MEFs treated by NH_2_-SSN-2 loaded FAM-siRNA at indicated time points post-transfection.*


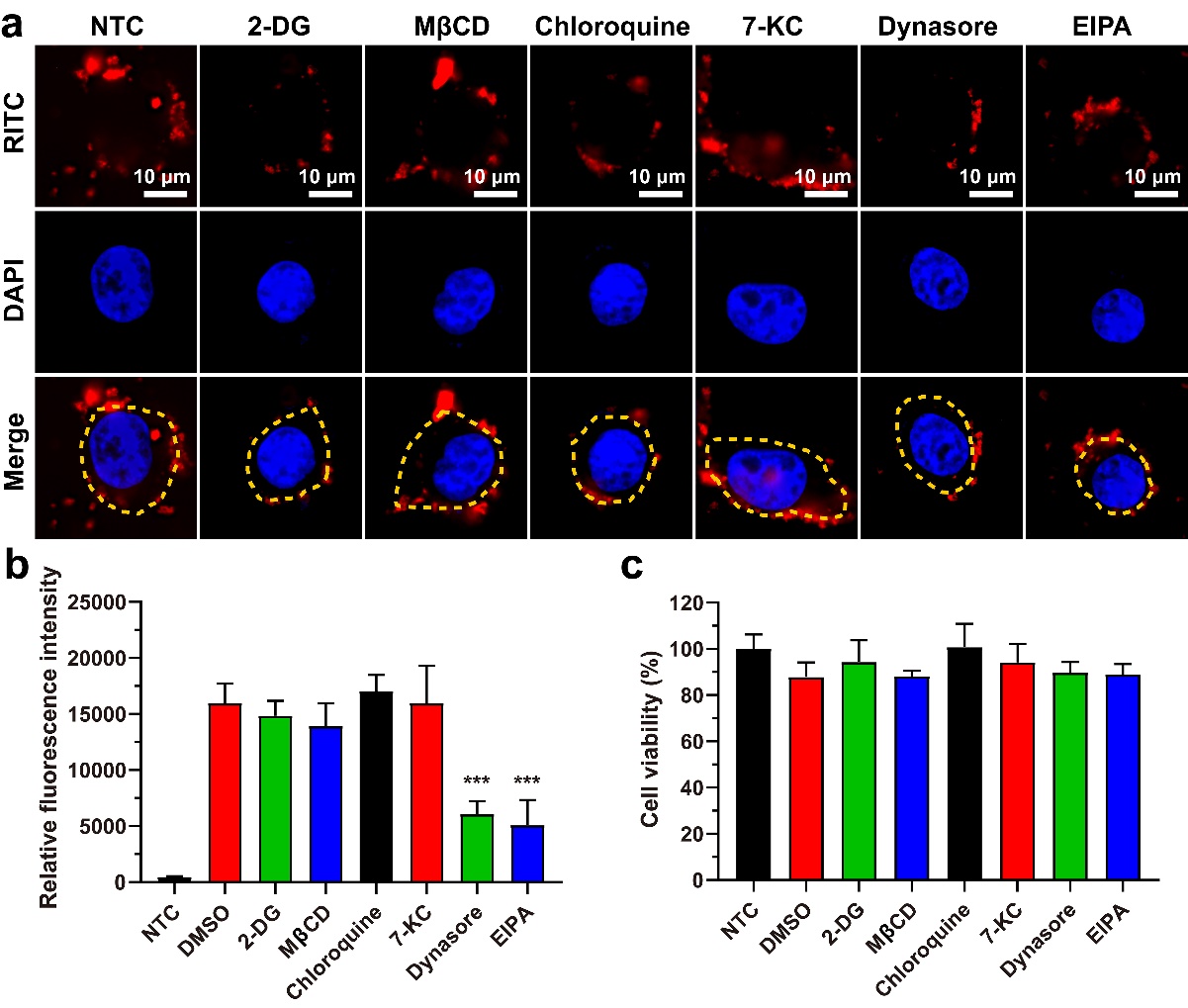


***Figure S8.*** *Fluorescence images showed the cellular internalization of RITC labeled NH_2_-SSN-2 when the internalization inhibitor was added (a), 2-DG inhibits all energy-dependent internalization(5 μM). MβCD inhibits the lipid rafts/cholesterol-enriched microdomains/caveolae pathway (1 μM). Chloroquine inhibits the CME pathway (1.13 μM). 7-KC inhibits the CLIC/GEEC pathway (5 μM). Dynasore inhibits the CME & FEME pathway (5 μM). EIPA inhibits macropinocytosis (1 μM). Quantitative measurement of intracellular amounts of FAM-siRNA (b). The relative fluorescence intensity of FAM was analyzed by ImageJ software. The cell viability of different inhibitors treated cells (c). Results were presented as mean ± SD and analyzed by one-way ANOVA. *p < 0.05,**p < 0.01，***p < 0.001.*


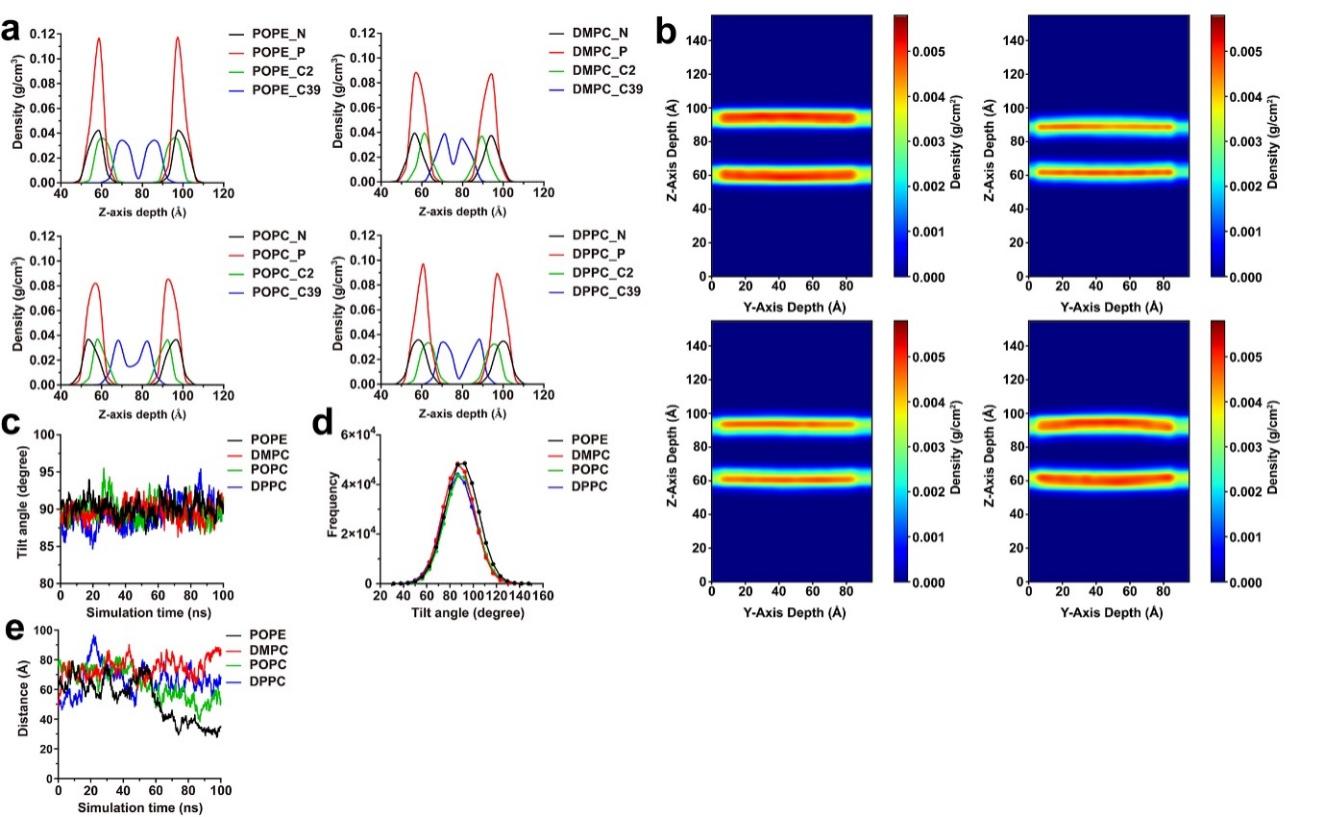


***Figure S9.*** *Mass density profile for N, P, C2, and C39 atoms along the Z-axis of four different lipid bilayers averaged over 100-ns MD simulations (a), Mass density of the lipid head group C2 atoms plotted along the Y and Z axis in POPE, DMPC, POPC and DPPC lipid bilayers (from left), simulated and averaged over 100-ns MD simulations (b), Stability of the lipid tilt angle during the 100-ns MD simulation (c), The lipid tilt angle calculated from the MD trajectory, averaged over the simulation time for POPE, DMPC, POPC and DPPC (d), The distance between the silica atom (Si973) on the tip of the SSN-2 spike to the closest lipid C2 atom in the upper leaflet of the lipid bilayer during the 100-ns MD simulation (e).*


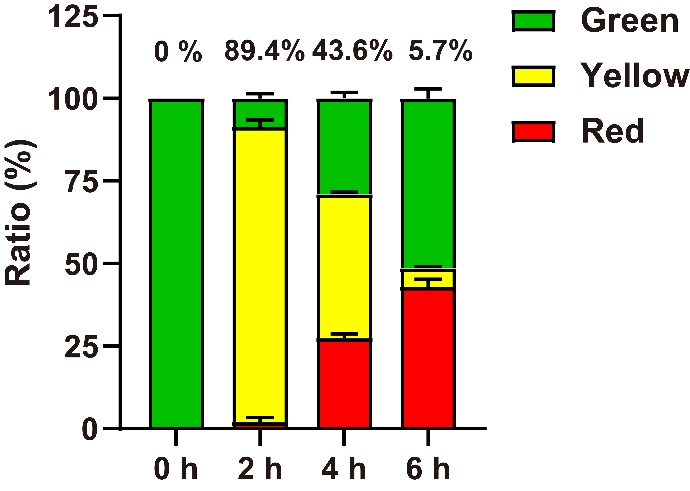


***Figure S10.*** *Percentage of co-localization/ separated red and green signals from Figure 5c. Cellular internalization of NH2-SSN-2 in MEFs.* *Green, Red and Yellow represent late endosome, NH2-SSN-2 and co-localization events, which were quantified by ImageJ software.*


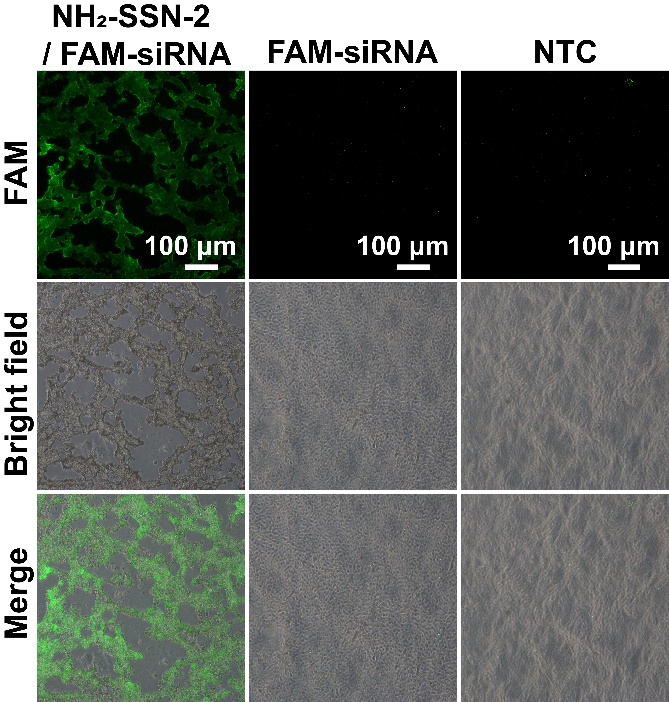


***Figure S11.*** *Fluorescence analysis of tumors 6-hour post-injection of NH_2_-SSN-2/FAM-siRNA formulation*

*
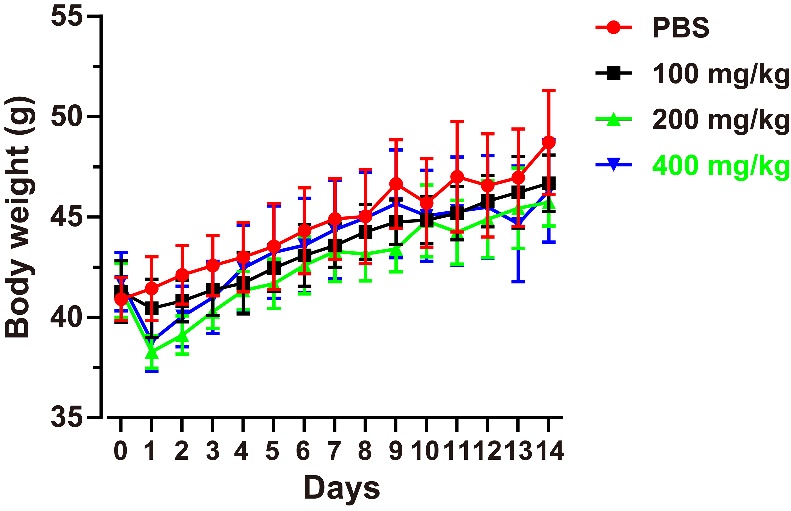
*

***Figure S12.*** *Assessment of mice body weight after a single-dose NH_2_-SSN-2. Results were presented as mean ± SD, n=7.*

***Table S1.*** *Textural and structural characteristics of SSNs and DMSNs*

| Samples | SBET (m2 g-1) | Vp (cm3 g-1) | Dp (nm) |
| --- | --- | --- | --- |
| SSN-1 | 346.7 | 0.76 | 6.8 |
| SSN-2 | 222.7 | 0.84 | 8.1 |
| SSN-3 | 294.5 | 1.38 | 12.7 |
| SSN-4 | 52.8 | 0.32 | 5.8 |
| DMSNs | 196.1 | 0.78 | 6.8 |

***Table S2.*** *Transfection efficiency of FAM-siRNA loaded NH_2_-SSN-2 in various cells.*

| Species | Cell lines | Delivery efficiency(%) |
| --- | --- | --- |
| Homo sapiens | HepG2 | 93.1±1.4 |
| Homo sapiens | A549 | 94.3±2.1 |
| Homo sapiens | 293T | 90.0±0.3 |
| Homo sapiens | HeLa | 93.2±0.5 |
| Homo sapiens | HCT116 | 94.0±3.4 |
| Homo sapiens | HGC27 | 90.1±0.9 |
| Homo sapiens | HT1080 | 98.1±0.3 |
| Mus musculus | B16F10 | 91.9±0.7 |
| Mus musculus | RAW264.7 | 95.0±2.6 |
| Mus musculus | MEF | 93.2±1.8 |
| Mus musculus | BMDM | 95.0±5.1 |
| Mus musculus | Normal Ovary Granule Cells | 90.3±0.5 |
| Trichoplax adhaerens Schulze | Primary cells | 97.0±1.3 |
| Chlorocebus sabaeus | VERO | 94.7±3.3 |

References

1. Robbins P. D., Ghivizzani S. C. Viral vectors for gene therapy. Pharmacol. Ther. 80, 35-47 (1998).

2. Angel J., Franco M. A., Greenberg H. B. Rotaviruses. In: Mahy B. W. J., Van Regenmortel M. H. V. (eds). Encyclopedia of virology (third edition). Academic Press: Oxford, 2008, pp 507-513.

3. Kay M. A., Glorioso J. C., Naldini L. Viral vectors for gene therapy: The art of turning infectious agents into vehicles of therapeutics. Nat. Med. 7, 33-40 (2001).

4. Schiedner G., Morral N., Parks R. J., Wu Y., Koopmans S. C., Langston C., et al. Genomic DNA transfer with a high-capacity adenovirus vector results in improved in vivo gene expression and decreased toxicity. Nat. Genet, 18, 180-183 (1998).

5. Shen C., Bradford Scott A., Imperiale Michael J. Why are viruses spiked? MSphere 6, e01339-01320 (2021).

6. Yang J., Zhang X., Liu C., Wang Z., Deng L., Feng C., et al. Biologically modified nanoparticles as theranostic bionanomaterials. Prog. Mater. Sci. 118, 100768 (2021).

7. Frouco G., Freitas F. B., Coelho J., Leitão A., Martins C., Ferreira F. DNA-binding properties of african swine fever virus pA104R, a histone-like protein involved in viral replication and transcription. J. Virol. 91, e02498 (2017).

8. Cubuk J., Alston J. J., Incicco J. J., Singh S., Stuchell-Brereton M. D., Ward M. D., et al. The sars-cov-2 nucleocapsid protein is dynamic, disordered, and phase separates with RNA. Nat. Commun. 12, 1936 (2021).

9. Nayak S., Herzog R. W. Progress and prospects: Immune responses to viral vectors. Gene Ther. 17, 295-304 (2010).

10. Whitehead K. A., Langer R., Anderson D. G. Knocking down barriers: Advances in siRNA delivery. Nat. Rev. Drug Discov. 8, 129-138 (2009).

11. Tomari Y. Perspective: Machines for RNAi. Genes. Dev. 19, 517-529 (2005).

12. Tang Z., Kong N., Zhang X., Liu Y., Hu P., Mou S., et al. A materials-science perspective on tackling COVID-19. Nat. Rev. Mater. 5, 847-860 (2020).

13. Tang Z., Zhang X., Shu Y., Guo M., Zhang H., Tao W. Insights from nanotechnology in COVID-19 treatment. Nano Today 36, 101019 (2021).

14. Akinc A., Maier M. A., Manoharan M., Fitzgerald K., Jayaraman M., Barros S., et al. The onpattro story and the clinical translation of nanomedicines containing nucleic acid-based drugs. Nat. Nanotechnol. 14, 1084-1087 (2019).

15. Mulligan M. J., Lyke K. E., Kitchin N., Absalon J., Gurtman A., Lockhart S., et al. Phase I/II study of COVID-19 RNA vaccine BNT162b1 in adults. Nature, 586, 589-593 (2020).

16. Zhu Y., Xu P., Zhang X., Wu D. Emerging porous organic polymers for biomedical applications. Chem. Soc. Rev. 51, 1377-1414 (2022).

17. Croissant J. G., Fatieiev Y., Khashab N. M. Degradability and clearance of silicon, organosilica, silsesquioxane, silica mixed oxide, and mesoporous silica nanoparticles. Adv. Mater. 29, 1604634 (2017).

18. Bukara K., Schueller L., Rosier J., Martens M. A., Daems T., Verheyden L., et al. Ordered mesoporous silica to enhance the bioavailability of poorly water-soluble drugs: Proof of concept in man. Eur. J. Pharm. Biopharm. 108, 220-225 (2016).

19. Song H., Yu M., Lu Y., Gu Z., Yang Y., Zhang M., et al. Plasmid DNA delivery: Nanotopography matters. J. Am. Chem. Soc. 139, 18247-18254 (2017).

20. Wang W., Wang P., Tang X., Elzatahry A. A., Wang S., Al-Dahyan D., et al. Facile synthesis of uniform virus-like mesoporous silica nanoparticles for enhanced cellular internalization. ACS Cent. Sci. 3, 839-846 (2017).

21. Niu Y., Yu M., Hartono S. B., Yang J., Xu H., Zhang H., et al. Nanoparticles mimicking viral surface topography for enhanced cellular delivery. Adv. Mater. 25, 6233-6237 (2013).

22. Lee C., Hwang H. S., Lee S., Kim B., Kim J. O., Oh K. T., et al. Rabies virus-inspired silica-coated gold nanorods as a photothermal therapeutic platform for treating brain tumors. Adv. Mater. 29, 1605563 (2017).

23. Häffner S. M., Parra-Ortiz E., Browning K. L., Jørgensen E., Skoda M. W. A., Montis C., et al. Membrane interactions of virus-like mesoporous silica nanoparticles. ACS Nano 15, 6787-6800 (2021).

24. Chen H. J., Hang T., Yang C., Liu D., Su C., Xiao S., et al. Functionalized spiky particles for intracellular biomolecular delivery. ACS Cent. Sci. 5, 960-969 (2019).

25. Kuo C. W., Lai J. J., Wei K. H., Chen P. Studies of surface-modified gold nanowires inside living cells. Adv. Funct. Mater. 17, 3707-3714 (2007).

26. Gratton S. E. A., Ropp P. A., Pohlhaus P. D., Luft J. C., Madden V. J., Napier M. E., et al. The effect of particle design on cellular internalization pathways. Proc. Natl. Acad. Sci. U S A 105, 11613-11618 (2008).

27. Wang Y., Tang J., Yang Y., Song H., Fu J., Gu Z., et al. Functional nanoparticles with a reducible tetrasulfide motif to upregulate mRNA translation and enhance transfection in hard-to-transfect cells. Angew. Chem. Int. Ed. 59, 2695-2699 (2020).

28. Wang Y., Du X., Liu Z., Shi S., Lv H. Dendritic fibrous nano-particles (DFNPs): Rising stars of mesoporous materials. J. Mater. Chem. A 7, 5111-5152 (2019).

29. Stöber W., Fink A., Bohn E. Controlled growth of monodisperse silica spheres in the micron size range. J. Colloid Interface Sci. 26, 62-69 (1968).

30. Carcouët C. C., Van De Put M. W., Mezari B., Magusin P. C., Laven J., Bomans P. H., et al. Nucleation and growth of monodisperse silica nanoparticles. Nano Lett. 14, 1433-1438 (2014).

31. Li S., Wan Q., Qin Z., Fu Y., Gu Y. Understanding Stӧber silica's pore characteristics measured by gas adsorption. Langmuir 31, 824-832 (2015).

32. Patwardhan S. V., Emami F. S., Berry R. J., Jones S. E., Naik R. R., Deschaume O., et al. Chemistry of aqueous silica nanoparticle surfaces and the mechanism of selective peptide adsorption. J. Am. Chem. Soc. 134, 6244-6256 (2012).

33. Mousavi M., Fini E. Silanization mechanism of silica nanoparticles in bitumen using 3-aminopropyl triethoxysilane (APTES) and 3-glycidyloxypropyl trimethoxysilane (GPTMS). ACS Sustain. Chem. Eng. 8, 3231-3240 (2020).

34. Babaei M., Eshghi H., Abnous K., Rahimizadeh M., Ramezani M. Promising gene delivery system based on polyethylenimine-modified silica nanoparticles. Cancer Gene. Ther. 24, 156-164 (2017).

35. Nguyen T. N., Angkawidjaja C., Kanaya E., Koga Y., Takano K., Kanaya S. Activity, stability, and structure of metagenome-derived LC11-RNase H1, a homolog of Sulfolobus tokodaii RNase H1. Protein Sci. 21, 553-561 (2012).

36. Mallapragada S., Agarwal A. Synthetic sustained gene delivery systems. Curr. Top. Med. Chem. 8, 311-330 (2008).

37. Brunner S., Sauer T., Carotta S., Cotten M., Saltik M., Wagner E. Cell cycle dependence of gene transfer by lipoplex, polyplex and recombinant adenovirus. Gene Ther. 7, 401-407 (2000).

38. Lim S. H., Liao I. C., Leong K. W. Nonviral gene delivery from nonwoven fibrous scaffolds fabricated by interfacial complexation of polyelectrolytes. Mol. Ther. 13, 1163-1172 (2006).

39. Bhatia P., Taylor W. R., Greenberg A. H., Wright J. A. Comparison of glyceraldehyde-3-phosphate dehydrogenase and 28S-ribosomal RNA gene expression as RNA loading controls for northern blot analysis of cell lines of varying malignant potential. Anal. Biochem. 216, 223-226 (1994).

40. Behzadi S., Serpooshan V., Tao W., Hamaly M. A., Alkawareek M. Y., Dreaden E. C., et al. Cellular uptake of nanoparticles: Journey inside the cell. Chem. Soc. Rev. 46, 4218-4244 (2017).

41. Kapara A., Brunton V., Graham D., Faulds K. Investigation of cellular uptake mechanism of functionalised gold nanoparticles into breast cancer using SERS. Chem. Sci. 11, 5819-5829 (2020).

42. Sun H., Wong E. H. H., Yan Y., Cui J., Dai Q., Guo J., et al. The role of capsule stiffness on cellular processing. Chem. Sci. 6, 3505-3514 (2015).

43. Teng Z., Wang C., Tang Y., Li W., Bao L., Zhang X., et al. Deformable hollow periodic mesoporous organosilica nanocapsules for significantly improved cellular uptake. J. Am. Chem. Soc. 140, 1385-1393 (2018).

44. Rennick J. J., Johnston A. P. R., Parton R. G. Key principles and methods for studying the endocytosis of biological and nanoparticle therapeutics. Nat. Nanotechnol. 16, 266-276 (2021).

45. Hernández N. E., Hansen W. A., Zhu D., Shea M. E., Khalid M., Manichev V., et al. Stimulus-responsive self-assembly of protein-based fractals by computational design. Nat. Chem. 11, 605-614 (2019).

46. Haag S. M., Gulen M. F., Reymond L., Gibelin A., Abrami L., Decout A., et al. Targeting sting with covalent small-molecule inhibitors. Nature, 559, 269-273 (2018).
